# circEIF3I facilitates the recruitment of SMAD3 to early endosomes to promote TGF-β signalling pathway-mediated activation of MMPs in pancreatic cancer

**DOI:** 10.1186/s12943-023-01847-2

**Published:** 2023-09-09

**Authors:** Zhongjie Zhao, Wenbo Yang, Rui Kong, Yangyang Zhang, Le Li, Zengfu Song, Hongze Chen, Yan Luo, Tao Zhang, Chundong Cheng, Guanqun Li, Danxi Liu, Xinglong Geng, Hua Chen, Yongwei Wang, Shangha Pan, Jisheng Hu, Bei Sun

**Affiliations:** 1 Department of Pancreatic and Biliary Surgery, The First Affiliated Hospital of Harbin Medical University, Harbin, 150001 Heilongjiang China; 2 Key Laboratory of Hepatosplenic Surgery, Ministry of Education, The First Affiliated Hospital of Harbin Medical University, Harbin, 150001 Heilongjiang China; 3 Department of Hepatobiliary and Pancreatic Surgery, Harbin Medical University Cancer Hospital, HarbinHeilongjiang, 150001 China; 4 Department of Hepatobiliary and Pancreaticosplenic Surgery, Beijing Chaoyang Hospital, Capital Medical University, Beijing, 100020 China

**Keywords:** Pancreatic cancer, circRNA, TGF-β, Early endosomes, Matrix metalloprotease

## Abstract

**Background:**

Among digestive tract tumours, pancreatic ductal adenocarcinoma (PDAC) shows the highest mortality trend. Moreover, although PDAC metastasis remains a leading cause of cancer-related deaths, the biological mechanism is poorly understood. Recent evidence demonstrates that circular RNAs (circRNAs) play important roles in PDAC progression.

**Methods:**

Differentially expressed circRNAs in normal and PDAC tissues were screened via bioinformatics analysis. Sanger sequencing, RNase R and actinomycin D assays were performed to confirm the loop structure of circEIF3I. In vitro and in vivo functional experiments were conducted to assess the role of circEIF3I in PDAC. MS2-tagged RNA affinity purification, mass spectrometry, RNA immunoprecipitation, RNA pull-down assay, fluorescence in situ hybridization, immunofluorescence and RNA–protein interaction simulation and analysis were performed to identify circEIF3I-interacting proteins. The effects of circEIF3I on the interactions of SMAD3 with TGFβRI or AP2A1 were measured through co-immunoprecipitation and western blotting.

**Results:**

A microarray data analysis showed that circEIF3I was highly expressed in PDAC cells and correlated with TNM stage and poor prognosis. Functional experiments in vitro and in vivo revealed that circEIF3I accelerated PDAC cells migration, invasion and metastasis by increasing MMPs expression and activity. Mechanistic research indicated that circEIF3I binds to the MH2 domain of SMAD3 and increases SMAD3 phosphorylation by strengthening the interactions between SMAD3 and TGFβRI on early endosomes. Moreover, AP2A1 binds with circEIF3I directly and promotes circEIF3I-bound SMAD3 recruitment to TGFβRI on early endosomes. Finally, we found that circEif3i exerts biological functions in mice similar to those of circEIF3I in humans PDAC.

**Conclusions:**

Our study reveals that circEIF3I promotes pancreatic cancer progression. circEIF3I is a molecular scaffold that interacts with SMAD3 and AP2A1 to form a ternary complex, that facilitates the recruitment of SMAD3 to early endosomes and then activates the TGF-β signalling pathway. Hence, circEIF3I is a potential prognostic biomarker and therapeutic target in PDAC.

**Supplementary Information:**

The online version contains supplementary material available at 10.1186/s12943-023-01847-2.

## Background

Pancreatic ductal adenocarcinoma (PDAC) is one of the most lethal digestive malignancies, with a 5-year overall survival rate of only 10% and a median survival of less than 12 months after diagnosis [1, 2]. Due to the lack of specific symptoms in the early stage, approximately 82% of patients with PDAC present with regional or distant metastasis at diagnosis [2], thus losing the opportunity of radical surgery. Furthermore, because of the extremely aggressive biological nature of PDAC, 75% of patients die from recurrence and metastasis within 5 years, even after radical resection [2]. Therefore, efficient early diagnostic biomarkers and potent therapeutic strategies for PDAC are urgently needed.

Circular RNAs (circRNAs) are a class of single-stranded closed RNA molecules that are formed by precursor mRNA back-splicing or skipping events of thousands of genes in eukaryotes [3]. Their stability, abundance, and evolutionary conservation among species indicate that circRNAs play important roles in cellular functions [3, 4]. Increasing evidence suggests that circRNAs affect the progression of cancers in various ways, such as functioning as microRNAs (miRNAs) sponges, decoys for proteins, scaffolds of circRNA–protein complexes and translation templates [5–10]. However, the precise function of most circRNAs remains ambiguous and needs to be determined.

The transforming growth factor-β (TGF-β) signalling pathway is highly activated in advanced PDAC and is associated with poor prognosis by promoting the epithelial-to-mesenchymal transition (EMT) and angiogenesis, maintaining tumour stem cell characteristics, and matrix metalloprotease (MMP) activity [11–16]. Canonical TGF-β signalling is mediated by downstream mothers against decapentaplegic homologue (SMAD) family proteins. SMAD2/3 are phosphorylated by activated TGF-β receptor I (TGFβRI) on the cell membrane and then combine with SMAD4 and translocate into the nucleus. In addition to the cell surface, SMAD2/3 are phosphorylated on early endosomes (EEs) derived from the endocytosis of activated receptors, where the cytoplasmic tail of the internalized receptor promotes SMAD2/3 activation [17–19].

In this study, we identified circEIF3I (circBase ID: hsa_circ_0011385) derived from the EIF3I gene by analysing the microarray data of circRNAs in PDAC tissues. circEIF3I was upregulated in PDAC tissues and was associated with poor prognosis. In addition, the downregulation of circEIF3I significantly inhibited pancreatic cancer cell invasion and metastasis in functional experiments. Mechanistically, we verified that circEIF3I functions as a molecular scaffold by binding with the MH2 domain of SMAD3 and AP2A1, which facilitates SMAD3 recruitment to TGFβRI on EEs and subsequent phosphorylation, thereby enhancing MMP activation mediated by the TGF-β signalling pathway. Moreover, we confirmed that circEif3i (circBase ID: mmu_circ_0001266), the homologous circRNA in mice, exerted similar physiological functions. Thus, our research indicates that circEIF3I is a potential biomarker that can be used to predict metastasis and an adverse prognosis, and targeting circEIF3I may prevent PDAC progression.

## Methods

All methods and materials are described in the Supplementary Methods.

## Results

### circEIF3I is highly expressed in PDAC and correlated with poor prognosis

**Fig. 1:**
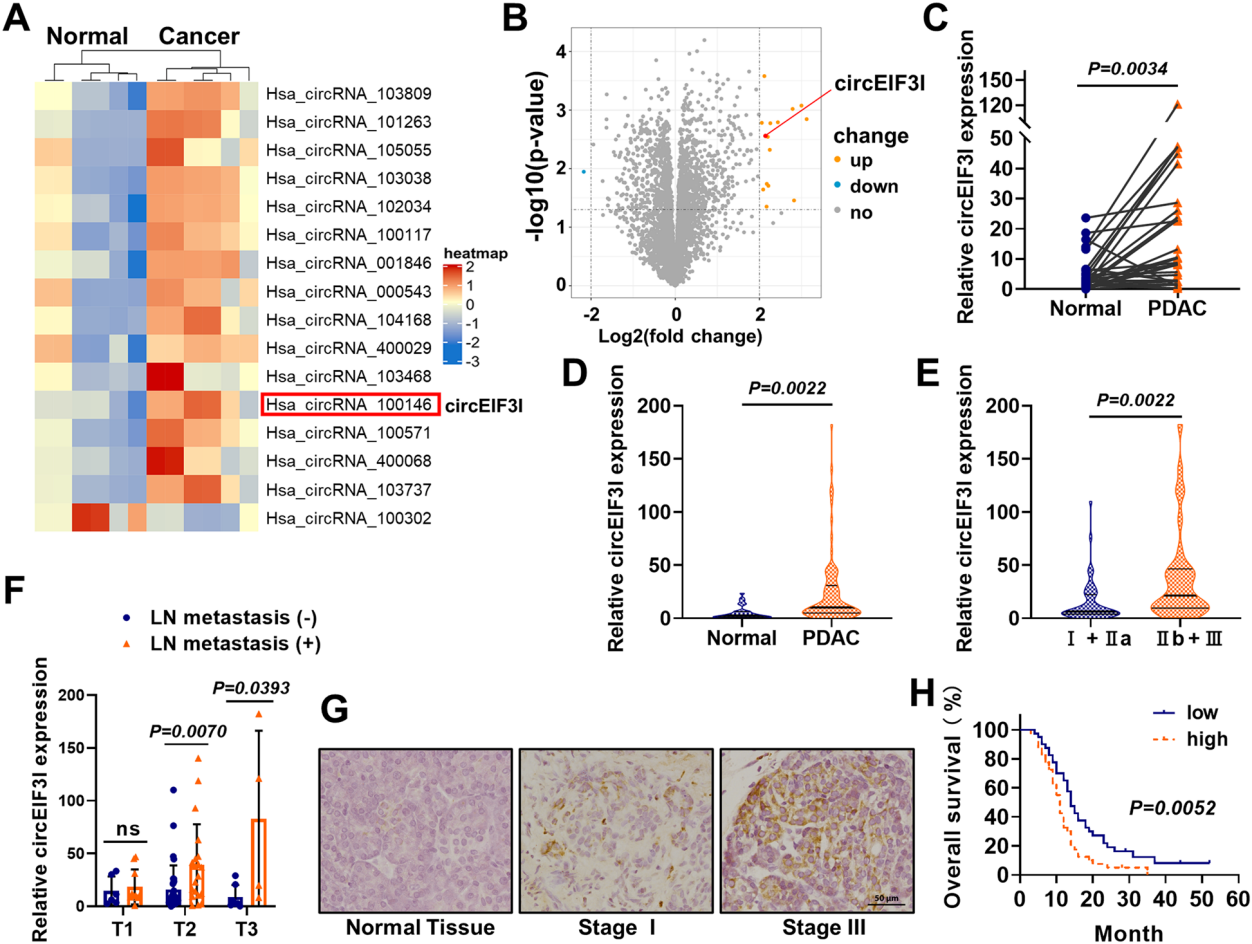
circEIF3I is highly expressed in PDAC and correlated with poor prognosis. **A** and **B** The heatmap (**A**) and volcano plot (**B**) showing circRNAs expression in 6 PDAC vs. 6 paired adjacent normal tissues (|log2(fold change)|> 2, *p* < 0.05). **C** circEIF3I expression in 30 pairs of PDAC tissues and adjacent normal tissues. **D** circEIF3I expression in 80 PDAC tissues and 30 adjacent normal tissues. **E** Association analyses of circEIF3I expression and TNM stage. **F** circEIF3I expression in pathologic T1, T2 and T3 stage of PDAC, with or without lymph node (LN) metastasis. **G** Representative images of the ISH staining analyses of different stage PDAC tissues using circEIF3I specific probe (Scale bar = 50 μm). **H** Kaplan–Meier analysis of postoperative overall survival (OS) for 80 PDAC patients with different circEIF3I expression. The low and high levels of circEIF3I expression are separated according to the median value. **P < 0.01; *P < 0.05; ns, not significant

To investigate the differential expression of circRNAs in PDAC, we acquired microarray data of 6 paired PDAC and adjacent normal tissues from the Gene Expression Omnibus (GEO, https://www.ncbi.nlm.nih.gov/geo/) dataset (GSE69362) and reperformed a bioinformatic analysis of differentially expressed circRNAs. The results of our bioinformatic analysis showed that the expression of 16 circRNAs was significantly altered (|log2(fold change)|> 2, *p* < 0.05), including 15 upregulated and 1 downregulated circRNAs. A heatmap and volcano plot revealed the distribution patterns of circRNAs (Fig. 1A, B). According to the Cancer-Specific CircRNA Database (CSCD, http://gb.whu.edu.cn/CSCD/), 10 of the upregulated circRNAs had been annotated as “cancer-related”. We then measured the expression of 10 circRNAs in 30 pairs of PDAC and adjacent normal tissues via qRT-PCR, and the results indicated that the level of circEIF3I (hsa_circ_100146) was the only circRNA that was significantly increased in PDAC tissues (Fig. 1C, Fig. S1).

We tested circEIF3I expression in an additional 50 pancreatic cancer tissues, and the results showed high expression of circEIF3I in all 80 cancer tissues compared to that in the 30 adjacent normal tissues, which further confirmed the upregulation of circEIF3I in PDAC (Fig. 1D, Supplementary Table S1). Furthermore, higher circEIF3I expression was associated with advanced clinical stage (Fig. 1E). For patients with tumours staged in T2 and T3, circEIF3I was upregulated in PDAC tissues with lymph node metastasis compared to those without lymphatic metastasis (Fig. 1F), which suggested that circEIF3I might correlate with tumour cell invasive capacity. In situ hybridization (ISH) staining demonstrated that circEIF3I expression was significantly and mildly increased in staged III and I PDAC tissues, respectively, compared to adjacent normal tissues (Fig. 1G). The survival curves indicated a correlation between high circEIF3I expression in PDAC and poor prognosis (Fig. 1H).

### Identification and characteristics of circEIF3I

**Fig. 2:**
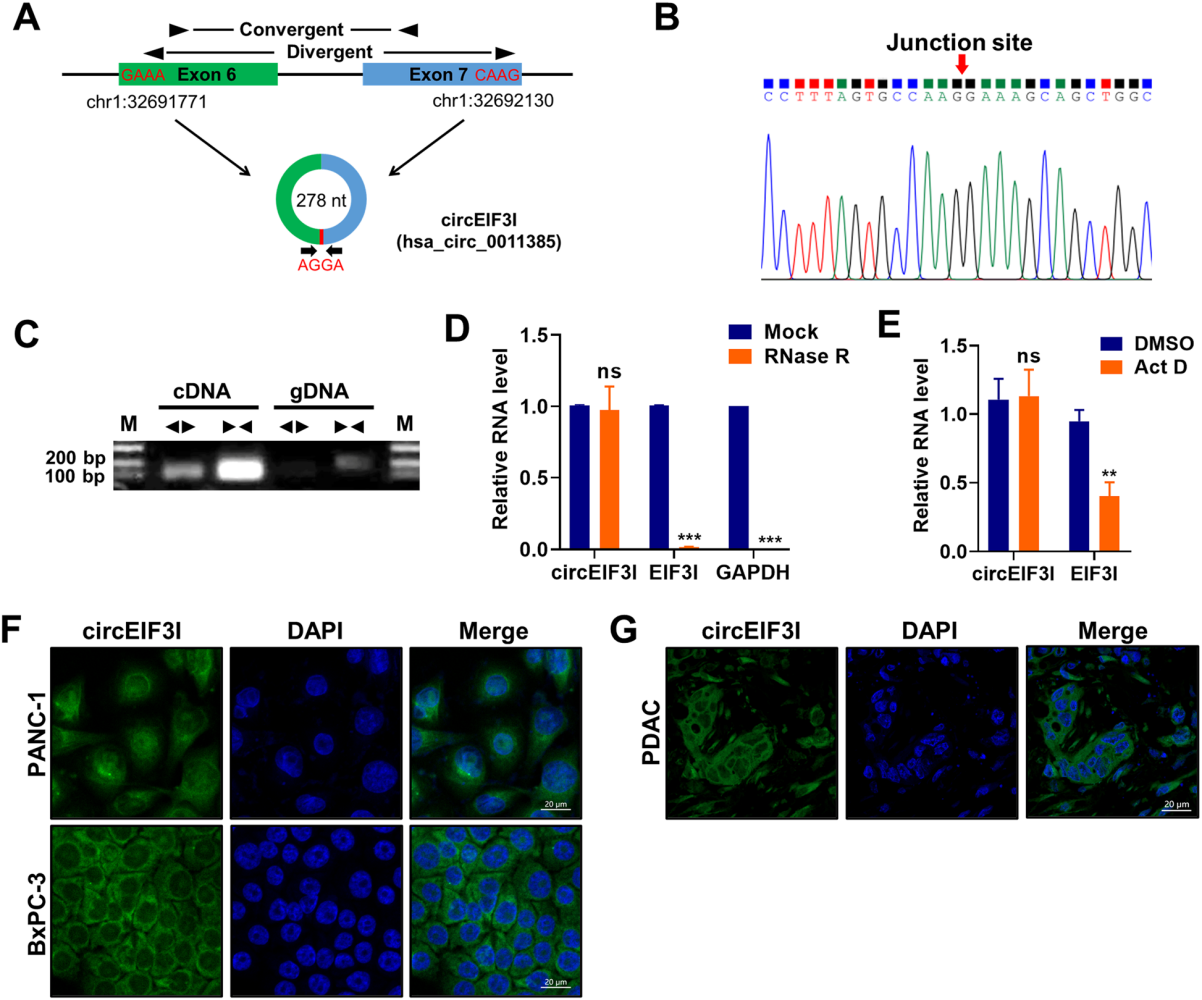
Identification and characteristics of circEIF3I. **A** Schematic diagram of circEIF3I generated from the Exon 6 and Exon 7 of EIF3I gene. **B** Sanger sequencing of head-to-tail splice junction in circEIF3I. **C** The agarose gel electrophoresis was performed using the divergent primers and convergent primers to identify the existence of circEIF3I. **D** qRT-PCR assay to determine circEIF3I, EIF3I mRNA, and GAPDH mRNA levels in samples after RNase R treatment. **E** qRT-PCR showed the stability of circEIF3I and EIF3I mRNA in pancreatic cancer cell treated with 5 μg/ml actinomycin D (Act D) for 24 h. **F** and **G** Representative FISH images identified the subcellular distribution of circEIF3I in pancreatic cancer cell (**F**) and PDAC tissues (**G**) using circEIF3I specific probe. The green (FAM-labelled probe) indicated the circEIF3I, while the blue (DAPI) indicated the nucleus (Scale bar = 10 μm). Data are shown as the mean ± SD of three replicates; **P < 0.01; ***P < 0.001; ns, not significant

circEIF3I (hsa_circ_0011385) is derived from the EIF3I gene located on human chromosome (chr) 1 and generated by Exon 6 and 7 back-splicing (278 nt) (Fig. 2A). Divergent primers were used to amplify the circEIF3I head-to-tail splice junction, which was confirmed via Sanger sequencing (Fig. 2B). The cDNA and genomic DNA were amplified using divergent primers and convergent primers, respectively, and then analysed by agarose gel electrophoresis. The results showed that only the cDNA produced an amplified PCR product with divergent primers; genomic DNA was not amplified, which was consistent with the closed loop construction (Fig. 2C). An RNase R treatment assay indicated that circEIF3I was relatively more stable, while EIF3I and GAPDH mRNA transcripts were degraded (Fig. 2D). The half-life of circEIF3I was much longer than that of the linear transcript after actinomycin D treatment, exhibiting the stability of circEIF3I (Fig. 2E). These results demonstrate that circEIF3I is bona fide cyclic structure. Fluorescence in situ hybridization (FISH) with PANC-1, BxPC-3 and PDAC tissues indicated that circEIF3I was predominantly located in the cytoplasm (Fig. 2F, G).

### circEIF3I promotes pancreatic cancer cell migration and invasion in vitro, and metastasis in vivo

**Fig. 3:**
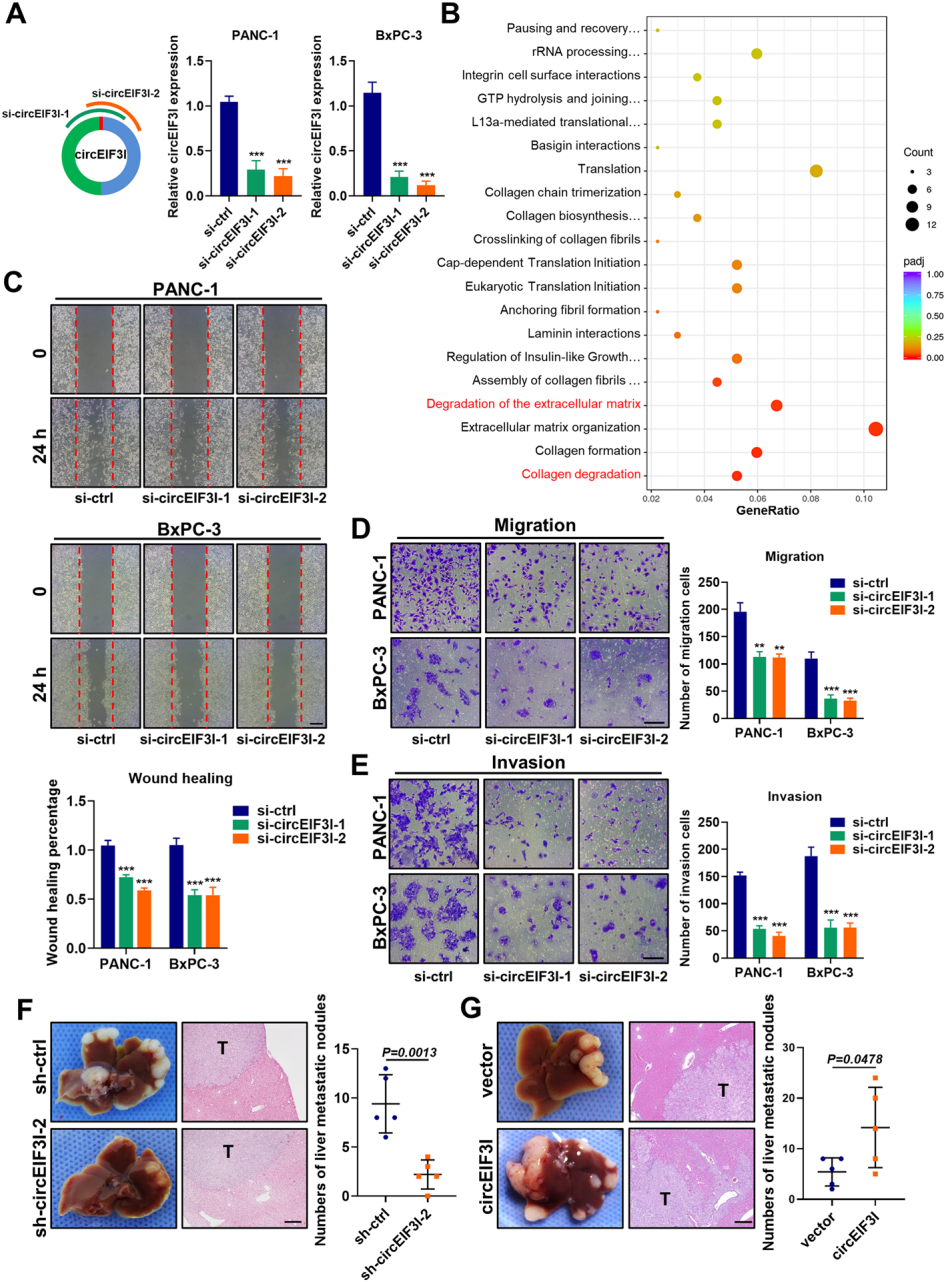
circEIF3I promotes pancreatic cancer cells migration and invasion in vitro, and metastasis in vivo*.* **A** Two circEIF3I siRNA were designed and qRT-PCR for circEIF3I expression after silencing of circEIF3I in PANC-1 and BxPC-3 cells. **B** The dot plots showing the enrichment analysis for representative pathway in circEIF3I target genes. **C**-**E** Wound healing assay (**C**), migration (**D**) and invasion assay (**E**) verified the effect of circEIF3I knockdown (KD) on PDAC cell migration and invasion ability via si-circEIF3I (Scale bar = 100 μm). **F** and **G** Representative photographs of the liver metastasis model established using cells with knocking down (**F**) and overexpressing (**G**) of circEIF3I, and HE staining of liver metastatic nodules with original magnification (Scale bar = 200 μm). The number of macroscopic metastases were calculated (right panel). Data are shown as the mean ± SD of three replicates; **P < 0.01; ***P < 0.001

Because circEIF3I expression was significantly correlated with TNM stage and lymphatic metastasis, we sought to explore whether circEIF3I regulates the biological behaviour of PDAC. We designed two small-interfering RNAs (siRNAs) specifically targeting the back-spliced junction in circEIF3I (Supplementary Table S2). A qRT-PCR analysis showed that siRNA transfection significantly downregulated the endogenous circEIF3I level (Fig. 3A), but exerted no effect on the expression of EIF3I mRNA (Fig. S2A). Based on the siRNAs sequence, we constructed hairpin RNA (shRNA) for circEIF3I using a lentiviral vector (GV112) and confirmed its knockdown efficiency and specificity (Fig. S2B, C). After establishing stably transfected PANC-1 cell lines (sh-ctrl, sh-circEIF3I-1 and sh-circEIF3I-2 cell lines), we performed a whole transcriptome analysis of PANC-1 cells with circEIF3I knockdown (sh-circEIF3I-2) group and control group via RNA sequencing. Pathway analysis (https://reactome.org/) revealed that circEIF3I knockdown (KD) inhibited the expression of genes enriched in the pathways of “collagen degradation” and “degradation of the extracellular matrix” (Fig. 3B), indicating that circEIF3I might be involved in the regulation of the pancreatic cancer metastatic phenotype, which was consistent with our clinical sample data.

Therefore, our hypothesis was primarily supported in vitro. Wound healing and Transwell assays demonstrated that circEIF3I knockdown using siRNAs significantly impaired the migratory and invasive capabilities of PANC-1 and BxPC-3 cells (Fig. 3C-E), and the same results were obtained in stably transfected cells (Fig. S2D, E). To further examine the biological functions of circEIF3I in vivo, we established orthotopic and liver metastasis mouse models using stably transfected PANC-1 cell lines. In the orthotopic tumour model, few metastatic nodes were observed in the intestinal wall and mesentery compared with those in the negative group (Fig. S2F). Similarly, the number of liver metastatic nodules in the sh-circEIF3I group was much lower than that in the sh-ctrl group (Fig. 3F, S2G), which was consistent with the in vitro phenotypes. However, the orthotopic tumour size showed no significant differences between the circEIF3I KD and control groups (Fig. S2H). Moreover, we constructed cell lines stably overexpressing circEIF3I (Fig. S2I), and demonstrated that circEIF3I overexpression in PANC-1 and BxPC-3 cells strengthened migration and invasion in vitro, and formed more metastatic nodes in vivo (Fig. 3G and Fig. S2J-M). Together, these results indicate that circEIF3I promotes pancreatic cancer cell migration and invasion in vitro, and metastasis in vivo.

### circEIF3I promotes invasion and metastasis by increasing MMP expression and activity

**Fig. 4:**
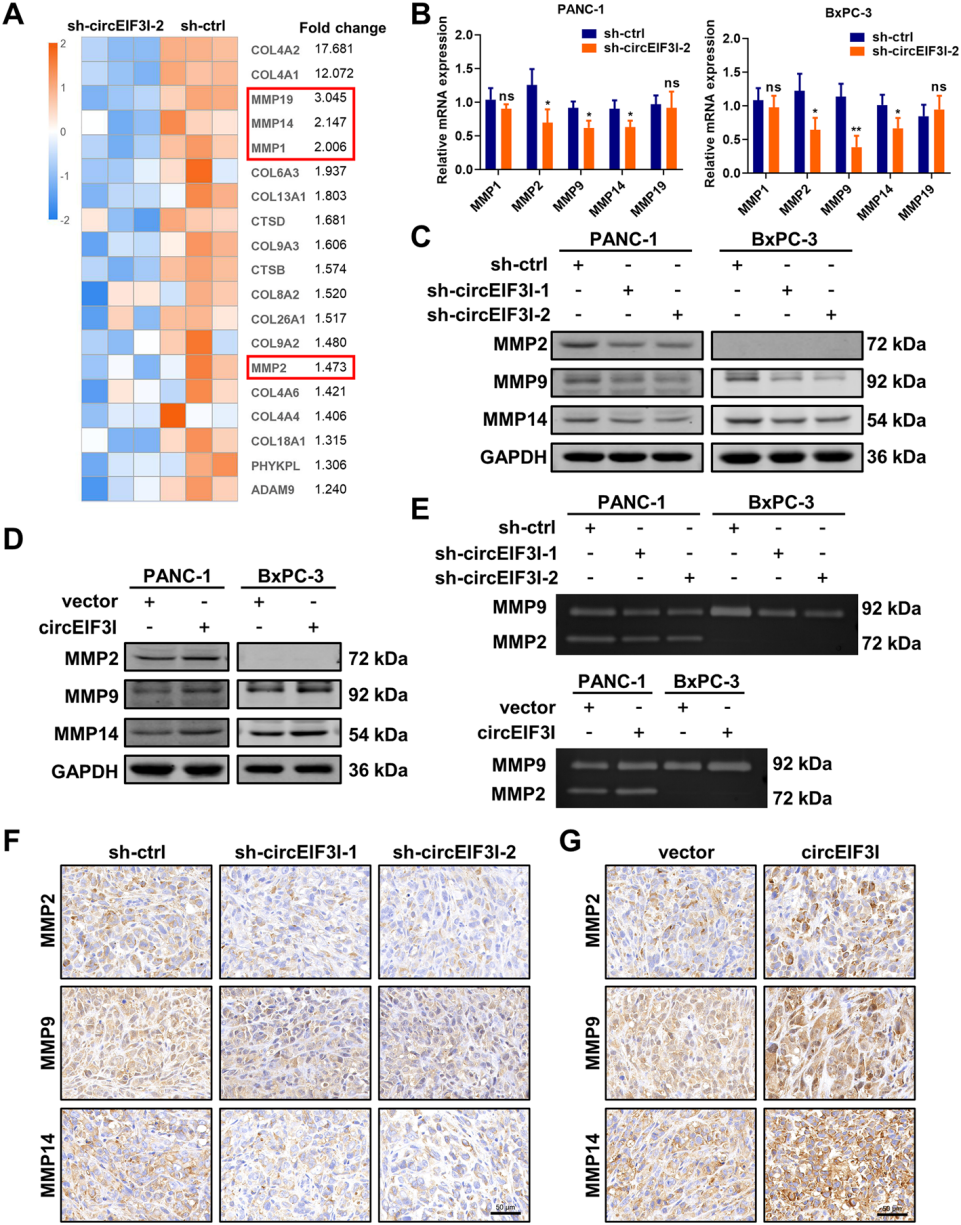
circEIF3I promotes invasion and metastasis by increasing MMPs expression and activity. **A** The heatmap showing the expression profiles of genes related to collagen degradation pathway. **B** qRT-PCR analyses of relative mRNA expression of MMP2, MMP9 and MMP14 in PANC-1 and BxPC-3 cells transfected with sh-ctrl or sh-circEIF3I-2. **C** and **D** The expression of MMP2, MMP9 (10 ng/ml PMA) and MMP14 was determined by western blot in PANC-1 and BxPC-3 cells with circEIF3I KD (**C**) and overexpression (**D**). **E** Gelatine zymography for conditioned media (10 ng/ml PMA, 24 h) of PANC-1 and BxPC-3 cells with circEIF3I KD and overexpression. **F** and **G** Representative images of immunohistochemistry analysis to detect the expression of MMP2, MMP9 and MMP14 in orthotopic tumours (Scale bar = 50 μm). Data are shown as the mean ± SD of three replicates; *P < 0.05; **P < 0.01; ns, not significant

Next, we sought to explore the potential mechanism by which circEIF3I promotes cancer cell migration and invasion. The expression profiles of enriched genes related to “collagen degradation” pathway were shown in the heatmap (Fig. 4A), in which the transcription levels of MMPs were decreased. qRT-PCR analysis with PANC-1 and BxPC-3 cells confirmed that circEIF3I KD inhibited the mRNA expression of MMP2, MMP9 and MMP14 (Fig. 4B), which are closely related to tumour invasion and metastasis. These results were verified by western blot analysis, which showed that circEIF3I KD caused a significant reduction in the protein levels of MMP2 (undetected in BxPC-3), MMP9 and MMP14 (Fig. 4C). In contrast, overexpression of circEIF3I resulted in an increase in the expression of MMP2, MMP9 and MMP14 (Fig. 4D). Meanwhile, gelatine zymography was performed to evaluate the activity of MMPs, and the results demonstrated that circEIF3I KD clearly decreased the activity of MMP2 and MMP9, while overexpression of circEIF3I led to an increase in MMP activity (Fig. 4E). In addition, we performed immunohistochemistry (IHC) with mouse xenograft tumour tissues to measure changes in MMP expression, and the results were consistent with those of western blotting (Fig. 4F, G). Importantly, the migratory and invasive capabilities of pancreatic cancer cells induced by circEIF3I were reduced by treatment with marimastat (10 μM), a broad-spectrum MMP inhibitors (Fig. S3A, B). Therefore, the aforementioned data suggest that circEIF3I promotes invasion and metastasis by increasing the expression and activities of MMP2, MMP9 and MMP14 in pancreatic cancer cells.

### circEIF3I directly binds to SMAD3 and increases SMAD3 phosphorylation

Then, we investigated the molecular mechanism underlying the regulation of MMP expression by circEIF3I. CircRNAs usually function by sponging miRNA; modulating gene expression in the nucleus; binding with proteins; and encoding proteins [4]. We first identified putative microRNA binding sites in circEIF3I with CircInteractome (https://circinteractome.nia.nih.gov/) and circBank (http://www.circbank.cn/index.html) data. However, only a single binding site was identified for all the miRNAs that may potentially bind circEIF3I (Fig. S4A), which signified that circEIF3I does not show a theoretical functional advantage as a ceRNA. In addition, qRT-PCR revealed that circEIF3I KD exerted no influence on the expression level of the potential binding miRNAs (Fig. S4B), indicating that circEIF3I may not function as a microRNA sponge [4, 20, 21]. Moreover, no appreciable changes were observed in mRNA or protein expression of the host gene (EIF3I) after circEIF3I KD (Fig. S2C, S4C), Therefore, together with the circEIF3I predominant cytosolic localization, direct modulation of gene transcripts in the nucleus was excluded as the circEIF3I mechanism of action. Moreover, after an analysis of circBank data, we did not find an open reading frame (ORF), internal ribosome entry site (IRES), or m6A motif in circEIF3I, excluding the possibility that it is translated into a peptide or protein. Therefore, we speculated that circEIF3I likely functions by interacting with proteins.

**Fig. 5:**
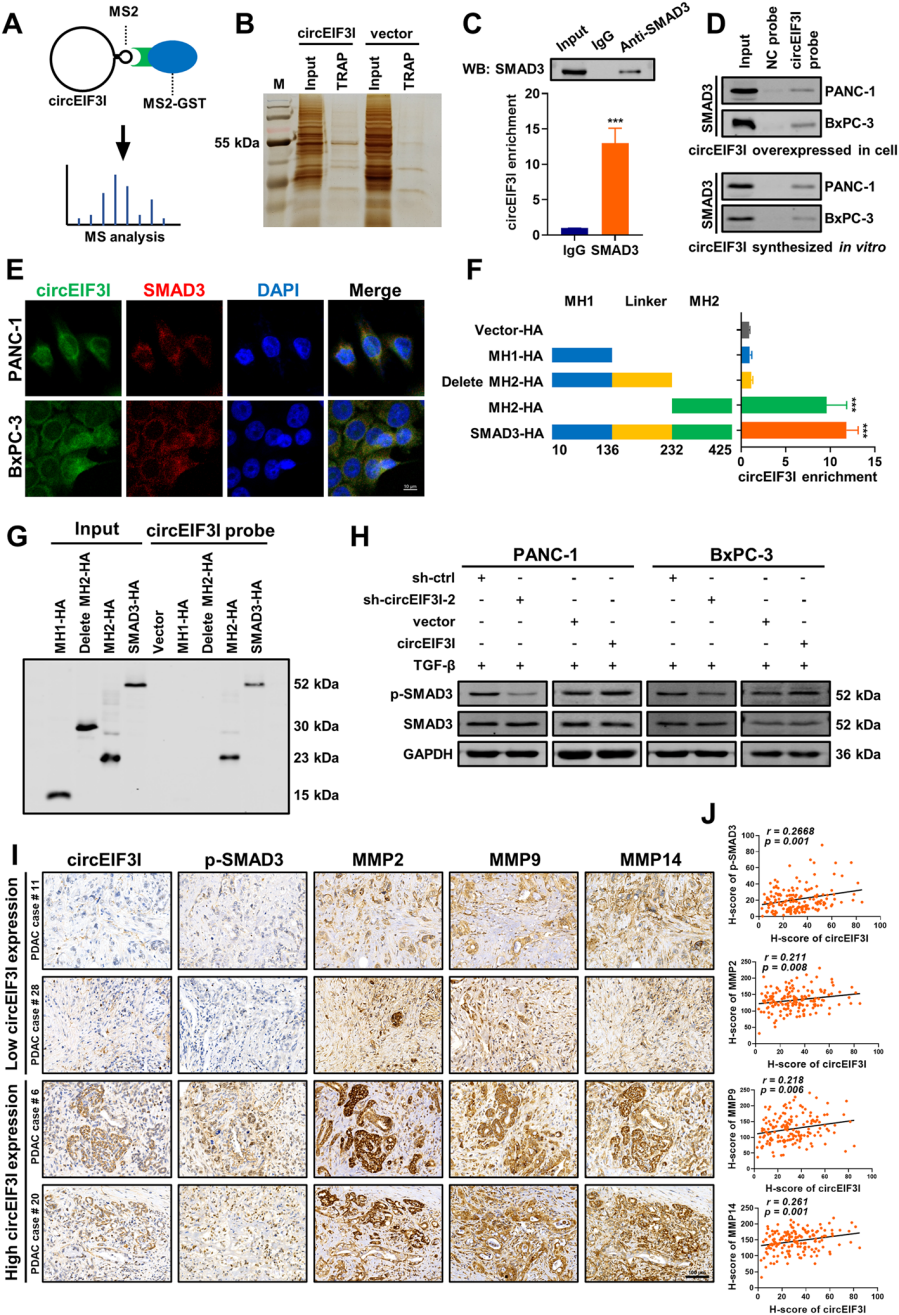
circEIF3I directly binds to SMAD3 and increases SMAD3 phosphorylation. **A** Schematic of MS2-GST protein-mediated capture of circEIF3I-MS2 from cellular lysates of overexpressing PANC-1 cell lines. **B** MS2-TRAP assay precipitated proteins were detected by silver staining. **C** The RNA immunoprecipitation (RIP) and qRT-PCR assays demonstrated specific binding of circEIF3I and SMAD3 (lower panel). IP efficiency of SMAD3 antibody shown by western blot (upper panel). **D** RNA pull-down assay showed the interaction between circEIF3I and SMAD3 in PANC-1 and BxPC-3 cells. **E** Representative FISH-IF images identified the colocalization of circEIF3I and SMAD3 in cytoplasm. The green (FAM-labelled probe) indicated the circEIF3I, the red indicated SMAD3, the blue (DAPI) indicated the nucleus (Scale bar = 10 μm). **F** RIP demonstrated the specific interaction between circEIF3I and MH2 domain of SMAD3. **G** Western blot analysis showed that circEIF3I retrieved HA-tagged full-length and SMAD3 MH2 domain using RNA pull-down assays. **H** Western blot showed the expression of p-SMAD3 and SMAD3 in cells with circEIF3I KD and overexpression, with the TGF-β treatment (5 ng/ml, 15 min). **I** Representative ISH and IHC images identified the expression of circEIF3I, p-SMAD3, MMP2, MMP9 and MMP14 derived from human PDAC tissue microarray (Scale bar = 100 μm). **J** Correlation analysis of histochemistry score (H-score) of circEIF3I with p-SMAD3, MMP2, MMP9 and MMP14, respectively. Data are shown as the mean ± SD of three replicates; ***P < 0.001

To validate our hypothesis, an MS2-tagged RNA affinity purification (MS2-TRAP) assay was performed to examine whether circEIF3I binds proteins (Fig. 5A). We generated PANC-1 cells overexpressing MS2 tagged circEIF3I, which allowed capture of circEIF3I-binding proteins in cellular lysates via high affinity interaction of the MS2 tag with the fusion protein MS2-GST. We first verified that the MS2-circEIF3I plasmid was overexpressed and properly cyclized in cells via qRT-PCR assay and by Sanger sequencing (Fig. S4D, E), and then validated that circEIF3I was highly enriched through bead-mediated capture (Fig. S4F). The precipitated proteins were analysed using silver staining and mass spectrometry (MS) (Fig. 5B, Supplementary Table S3). Among the proteins that potentially bound circEIF3I, SMAD3 has been shown to be required in MMP expression and activation [16, 22, 23]. Therefore, we identified SMAD3 as a potential target. RNA immunoprecipitation (RIP) demonstrated that circEIF3I was specifically precipitated by SMAD3 (Fig. 5C). Moreover, we further confirmed the direct binding between circEIF3I and SMAD3 through RNA pull-down experiments using circEIF3I overexpressed in HEK-293 T cells (up panel) and synthesized in vitro (down panel) (Fig. 5D, S4H). qRT-PCR analysis verified that the biotin-labelled probe for circEIF3I enriched circEIF3I efficiently and specifically (Fig. S4I). We also conducted FISH-immunofluorescence (FISH-IF) analysis and found that circEIF3I colocalized with SMAD3 in the cytoplasm (Fig. 5E). The SMAD3 protein consists of two globular domains (termed MH1 and MH2) that are coupled by a linker region [24]. Therefore, we established four HA-tagged vectors to identify which domain interacts with circEIF3I. The RIP assay showed that circEIF3I precipitated mostly via its attachments to the SMAD3 MH2 domain in HEK-293 T cells (Fig. 5F), suggesting that the MH2 domain was critical for SMAD3 binding to circEIF3I. RNA pull-down assay with full-length and truncated SMAD3 also indicated that circEIF3I interacted with the MH2 domain of SMAD3 (Fig. 5G).

After confirming that SMAD3 directly binds to circEIF3I, we investigated whether circEIF3I regulates SMAD3 expression or its modification. A western blot analysis showed that inhibition or overexpression of circEIF3I led to a decrease or increase in phosphorylated-SMAD3 (p-SMAD3 Ser423/425) after TGF-β (5 ng/ml) treatment, without affecting the total expression of SMAD3, indicating that circEIF3I might regulate SMAD3 phosphorylation, not SMAD3 expression (Fig. 5H). An IHC analysis of xenograft tumour sections showed results consistent with the western blot analysis (Fig. S4J). Furthermore, the upregulation of MMP2, MMP9 and MMP14 induced by circEIF3I was inhibited by treatment with SIS3 (15 μM), a selective inhibitor of SMAD3 phosphorylation (Fig. S4K, S7B). These results strongly suggest that circEIF3I directly binds to SMAD3 and promotes its phosphorylation, leading to the upregulated expression of MMPs.

To confirm that the circEIF3I/TGF-β/MMP axis functions in PDAC patients, we detected the expression of circEIF3I, p-SMAD3, MMP2, MMP9 and MMP14 in human PDAC tissue microarrays via ISH and IHC experiments. The p-SMAD3 and MMPs were consistently found to be downregulated or upregulated in PDAC patients with low circEIF3I expression *vs.* those with high circEIF3I expression (Fig. 5I). A correlation analysis of the histochemistry score (H-score) revealed that circEIF3I expression was positively correlated with pSMAD3 and MMPs expression (Fig. 5J).

### circEIF3I facilitates recruitment of SMAD3 to TGFβRI on early endosomes

**Fig. 6:**
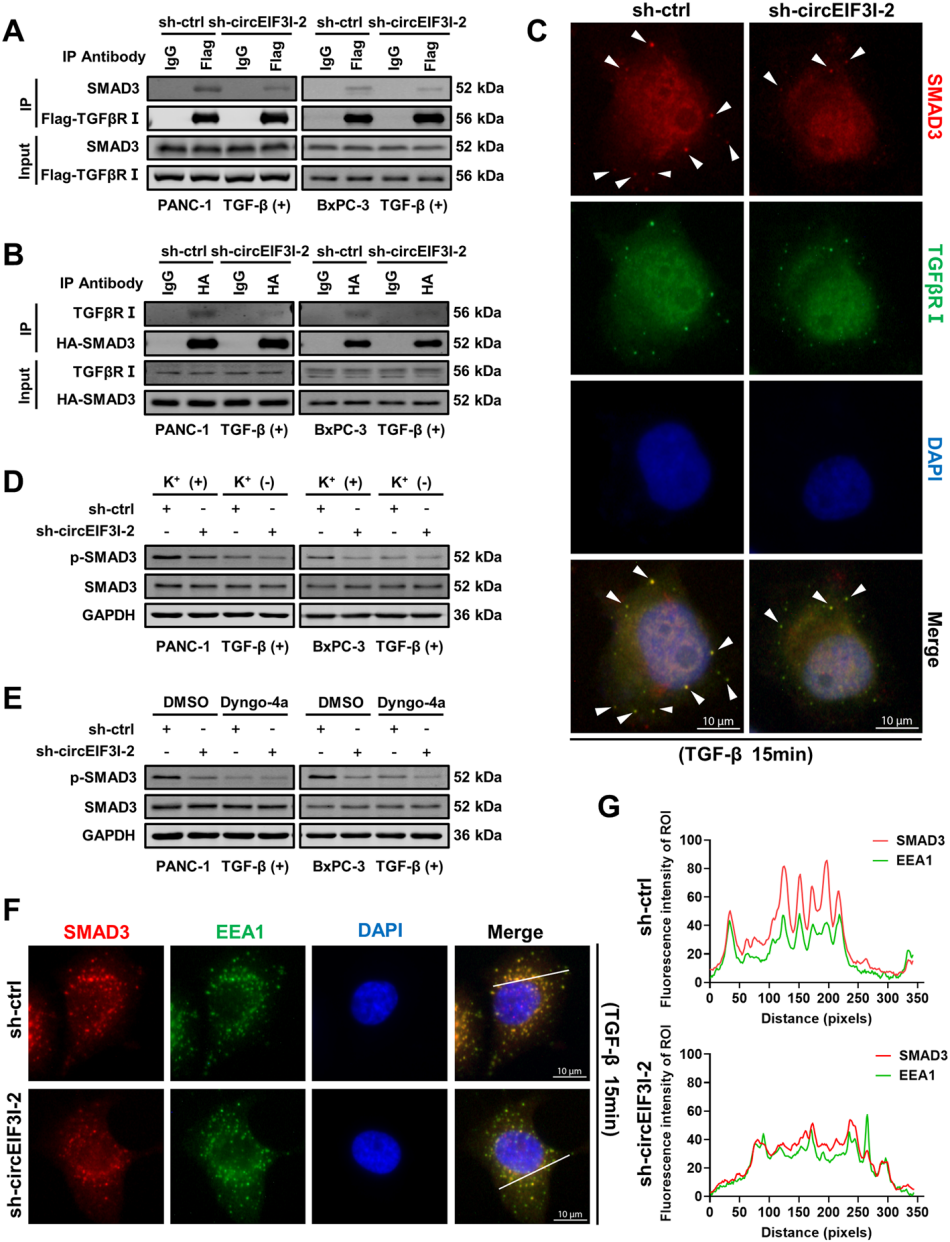
circEIF3I facilitates recruitment of SMAD3 to TGFβRI on early endosomes. **A** and **B** Western blot analysis of indicated proteins in Flag-TGFβRI immunoprecipitated complexes or in HA-SMAD3 immunoprecipitated complexes from lysates of PANC-1 and BxPC-3 with circEIF3I KD (5 ng/ml TGF-β, 15 min). **C** Representative IF images identified the colocalization of SMAD3 (white arrows) and TGFβRI in PANC-1 cells transfected with sh-ctrl or sh-circEIF3I-2 (5 ng/ml TGF-β, 15 min). The red indicated SMAD3, the green indicated the TGFβRI, the blue (DAPI) indicated the nucleus (Scale bar = 10 μm). **D** and **E** Western blot showed the p-SMAD3 expression (5 ng/ml TGF-β, 15 min) in cells transfected with sh-ctrl or sh-circEIF3I-2, with or without the endocytosis blocked by potassium depletion or Dyngo-4a (30 μM). **F** Representative IF images identified the colocalization of SMAD3 and EEA1 in PANC-1 cells transfected with sh-ctrl or sh-circEIF3I-2 (5 ng/ml TGF-β, 15 min). The red indicated SMAD3, the green indicated the EEA1, the blue (DAPI) indicated the nucleus (Scale bar = 10 μm). **G** The fluorescence intensity of regions of interest (ROI) was quantified along with the indicated white dashed using ImageJ software

We next sought to further investigate the molecular mechanism by which circEIF3I facilitates the increase in SMAD3 phosphorylation. SMAD3 is phosphorylated by interacting with activated TGFβRI immediately after TGF-β successively activates the TGF-β receptor II (TGFβRII) and TGFβRI on cytomembrane [17, 25, 26]. As the initiation element, membrane receptors are critical for regulating the activation of the TGF-β pathway [27]; therefore, we first performed western blotting to measure the expression of TGFβRI and TGFβRII after circEIF3I KD. The results showed that circEIF3I exerted no effect on the expression of TGFβRI or TGFβRII (Fig. S5A). Hence, we speculated that SMAD3 binding of circEIF3I enhances the interaction between SMAD3 and TGFβRI. Co-immunoprecipitation (co-IP) experiments showed that circEIF3I KD impaired the binding between SMAD3 and TGFβRI (Fig. 6A, B). An IF assay also confirmed the decreased colocalization of SMAD3 and TGFβRI in sh-circEIF3I-2 group compared to the sh-ctrl group (Fig. 6C). These data indicate that circEIF3I accelerates SMAD3 recruitment to TGFβRI.

In addition to activating SMAD3 on the cell surface, activated TGFβRI and TGFβRII on the cell membrane can trigger the internalization of both TGF-β and tetrameric TGF-β receptor complexes and fuse with early endosomes (EEs), which have also been confirmed to induce SMAD3 activation directly [17–19, 28–30]. To explore whether SMAD3 phosphorylation mediated by circEIF3I takes place on the cytomembrane or EEs, potassium depletion and a dynamin inhibitor (Dyngo-4a, 30 μM) were applied to block TGF-β receptor endocytosis [31, 32]. A Western blot analysis showed that p-SMAD3 levels that had been increased by circEIF3I were significantly reduced after endocytosis was supressed (Fig. 6D, E; Fig. S5B, C). Furthermore, IF results showed that the level of SMAD3 located on early endosomes (EEA1) was reduced after circEIF3I was knocked down (Fig. 6F, G); however, more SMAD3 was localized to EEs in cells overexpressing circEIF3I (Fig. S5D, E). These results suggest that circEIF3I-mediated recruitment of SMAD3 to TGFβRI mainly on EEs, rather than on the plasma membrane.

### circEIF3I promotes the recruitment of SMAD3 to early endosomes by binding with AP2A1

**Fig. 7:**
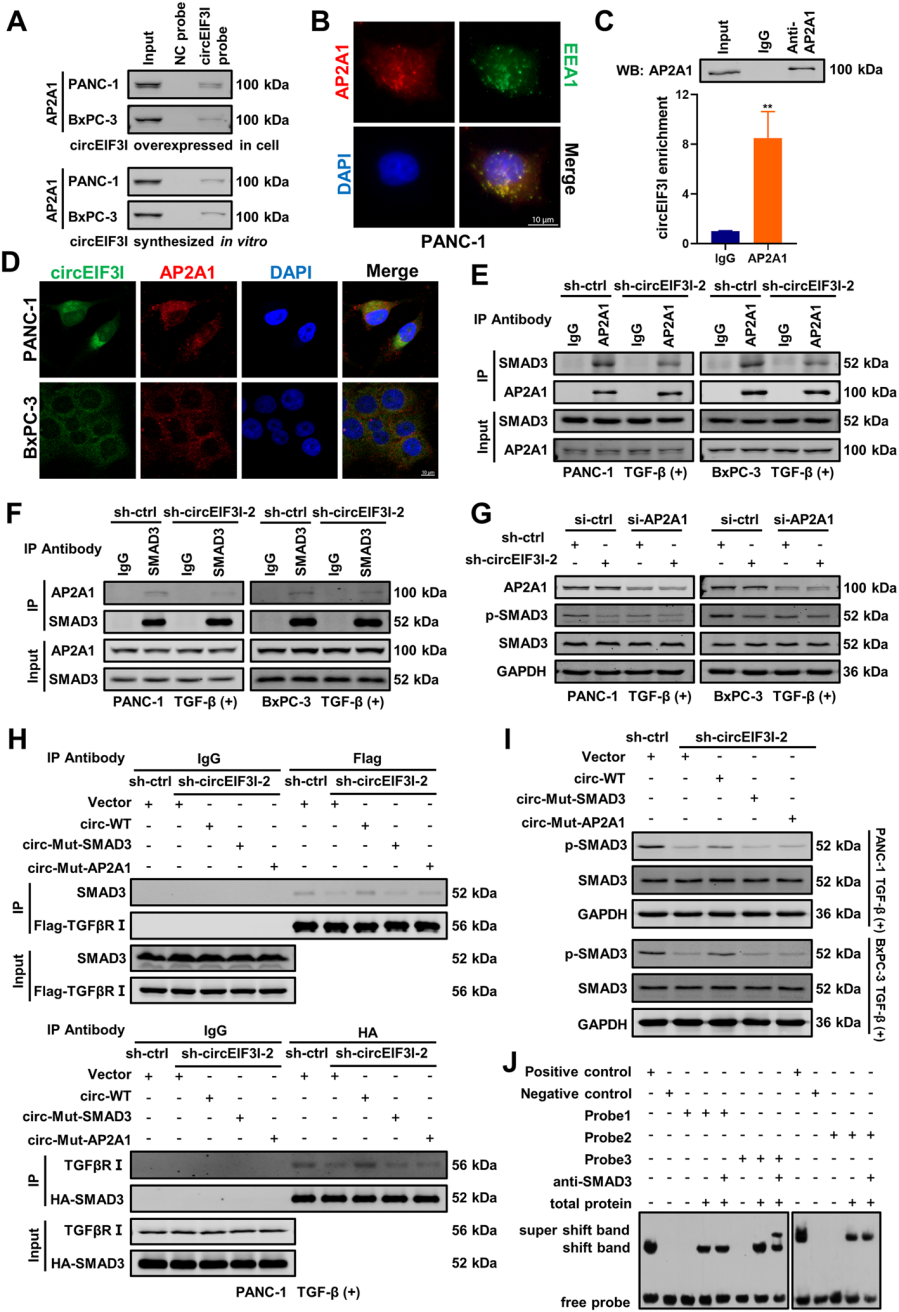
circEIF3I promotes the recruitment of SMAD3 to early endosomes via binding with AP2A1. **A** RNA pull-down and western blot assays showed the interaction between circEIF3I and AP2A1 in PANC-1 and BxPC-3 cells. **B** Representative IF images identified the enrichment of AP2A1 on early endosomes (EEs) in PANC-1 cells. The red indicated AP2A1, the green indicated the EEA1 (marker of EEs), the blue (DAPI) indicated the nucleus (Scale bar = 10 μm). **C** The RNA immunoprecipitation (RIP) and qRT-PCR assays demonstrated specific binding of circEIF3I and AP2A1 (lower panel). IP efficiency of AP2A1 antibody shown by western blot (upper panel). **D** Representative FISH-IF images identified the colocalization of circEIF3I and AP2A1 in cytoplasm. The green (FAM-labelled probe) indicated the circEIF3I, the red indicated AP2A1, the blue (DAPI) indicated the nucleus (Scale bar = 10 μm). **E** and **F** Western blot analysis of indicated proteins in AP2A1 immunoprecipitated complexes or SMAD3 immunoprecipitated complexes from lysates of PANC-1 and BxPC-3 with circEIF3I KD, which were treated with 5 ng/ml TGF-β for 15 min. **G** Western blot showed the p-SMAD3 expression in PANC-1 and BxPC-3 cells transfected with sh-ctrl or sh-circEIF3I-2, with or without AP2A1 KD (5 ng/ml TGF-β, 15 min). **H** The binding of SMAD3 and TGFβR I in PANC-1 transfected with sh-ctrl or sh-circEIF3I-2, with the ectopic expression of circEIF3I-WT, circ-mut-SMAD3 and circ-mut-AP2A1 respectively in circEIF3I KD groups (5 ng/ml TGF-β, 15 min). **I** The p-SMAD3 expression in PANC-1 and BxPC-3 cells transfected with sh-ctrl or sh-circEIF3I-2, with the ectopic expression of circEIF3I-WT, circ-mut-SMAD3 and circ-mut-AP2A1 respectively in circEIF3I KD groups (5 ng/ml TGF-β, 15 min). **J** RNA-EMSA determined the specific binding between SMAD3 and biotin-labelled Probe3 of circEIF3I, with SMAD3 antibody incubation (super shift band). Data are shown as the mean ± SD of three replicates; **P < 0.01

We previously demonstrated that circEIF3I directly bound to SMAD3 and boosted its recruitment to TGFβRI on EEs. Then, we assessed whether TGFβRI directly binds with circEIF3I to recruit SMAD3 to EEs. However, the RNA pull-down results proved that circEIF3I did not directly bind to TGFβRI (Fig. S6A). Therefore, we speculated that another protein binds circEIF3I and facilitates the recruitment of SMAD3 to TGFβRI on EEs. In the aforementioned MS datas (Supplementary Table S3), we found that 4 proteins (ANXA2, TRIP10, AP2A1 and PACSIN2) were closely associated with endocytosis and were enriched on EEs. RNA pull-down experiments verified the specific binding between AP2A1 and circEIF3I (Fig. 7A, S6B). AP2A1 is a subunit of the AP2 complex, which plays a crucial role in clathrin-mediated endocytosis [33–36], and through an IF assay, we confirmed that AP2A1 was highly enriched on the endosomal membrane (Fig. 7B, S6C). We performed a RIP experiment to verify that circEIF3I was specifically precipitated by AP2A1 (Fig. 7C). Similar to SMAD3, AP2A1 colocalized with circEIF3I in the cytoplasm (Fig. 7D). Moreover, reciprocal co-IP assays showed that SMAD3 physically interacted with AP2A1, and the knockdown of circEIF3I impaired the binding of SMAD3 and AP2A1 (Fig. 7E, F). We designed siRNA (Supplementary Table S2) to suppress the expression of AP2A1, and western blotting indicated that circEIF3I-mediated phosphorylation of SMAD3 was significantly inhibited after AP2A1 knockdown (Fig. 7G). Together, these results verify that AP2A1 combines with circEIF3I and SMAD3 and enhance circEIF3I-induecd SMAD3 phosphorylation.

We next explored the probable binding sites of SMAD3 and AP2A1 on circEIF3I via molecular docking analyses. Considering the secondary structure of circEIF3I with the lowest free energy (ΔG = -78.50 kcal/mol), we established three-dimensional (3D) models of circEIF3I in complex with SMAD3 and AP2A1 (Fig. S6D, E). The structural simulation showed that SMAD3 and AP2A1 interacted with different regions of circEIF3I, which suggested that these three molecules form a complex without a spatial conformation conflict (Fig. S6F). The molecular simulation also revealed the crucial bases of circEIF3I binding with SMAD3 and AP2A1. Two atoms in RNA and amino acid residues were thought to interact with each other when they were within 3.5 Å proximity (Supplementary Table S4). These key bases in circEIF3I were mutated and two mutation plasmids (circ-mut-SMAD3 and circ-mut-AP2A1) were constructed (Fig. S6G, H). We performed RNA pull-down assay to evaluate the protein precipitated through wild-type circEIF3I and mutant circEIF3I from circEIF3I KD cells. The results suggested that circ-mut-SMAD3 completely abolished the circEIF3I-SMAD3 interaction, while the binding of circEIF3I and AP2A1 was retained. In contrast, circ-mut-AP2A1 maintained the SMAD3 interaction but was not competent to precipitate AP2A1 (Fig. S6I). Moreover, we ectopically expressed circEIF3I-WT, circ-mut-SMAD3 and circ-mut-AP2A1 in circEIF3I KD cells. Co-IP assays indicated that the interaction between SMAD3 and TGFβRI was rescued in the circEIF3I-WT group but not in the circ-mut-SMAD3 or circ-mut-AP2A1 group (Fig. 7H, S6J). In addition, the p-SMAD3 level was partially recovered in the circEIF3I-WT group, consistent with the co-IP results (Fig. 7I). Moreover, cells in the circEIF3I-WT group regained greater motility and invasive capacities than those in the circ-mut-SMAD3 and circ-mut-AP2A1 groups, as indicated by Transwell assays (Fig. S6K, L). We ectopically expressed circEIF3I-WT by simultaneously knocking down AP2A1 or treating circEIF3I KD cells with SIS3, and p-SMAD3 and MMP protein expression was again suppressed (Fig. S7A, B).

Additionally, we performed RNA electrophoretic mobility shift assay (RNA-EMSA), in which probes were designed according to molecular docking outcomes (Fig. S7C), and the results validated that probe3 specifically bound to SMAD3, while AP2A1 specifically interacted with both probe2 and probe3 (Fig. 7J, D). On the other side, we identified the RNA motif required for binding with SMAD3 and AP2A1 via RIP-seq (Supplementary Table S5). By browsing the sequence of RNA-EMSA probes, we recognized the SMAD3 binding motif UUUAAAA was located in probe3 and the AP2A1 binding motif CAACWWC (W = A or U) was in probe2 (Fig. S7E). Furthermore, the motifs in probe3 and probe2 carried the key bases in docking analysis, which further increased the confidence that these two motifs were responsible for binding to SMAD3 and AP2A1. Besides, we also constructed mutant plasmids of the MH2 domain (HA-MH2-Mut) in SMAD3, which led to key amino acids (proximity < 3.5 Å) replacement with alanine [37] (Fig. S7F). Then, we performed RIP and RNA pull-down assays, which revealed a noticeable decline in the binding ability of HA-MH2-Mut compared to that of HA-MH2-WT with circEIF3I (Fig. S7G, H), confirming our previous finding that the MH2 domain of SMAD3 is critical for binding with circEIF3I. In summary, these results strongly suggest that circEIF3I functions as a molecular scaffold with SMAD3 and AP2A1 to form a ternary complex which facilitates the recruitment of SMAD3 to EEs and then activates TGF-β signalling pathway.

### Homologous circEif3i in mouse performs similar physiological functions

**Fig. 8:**
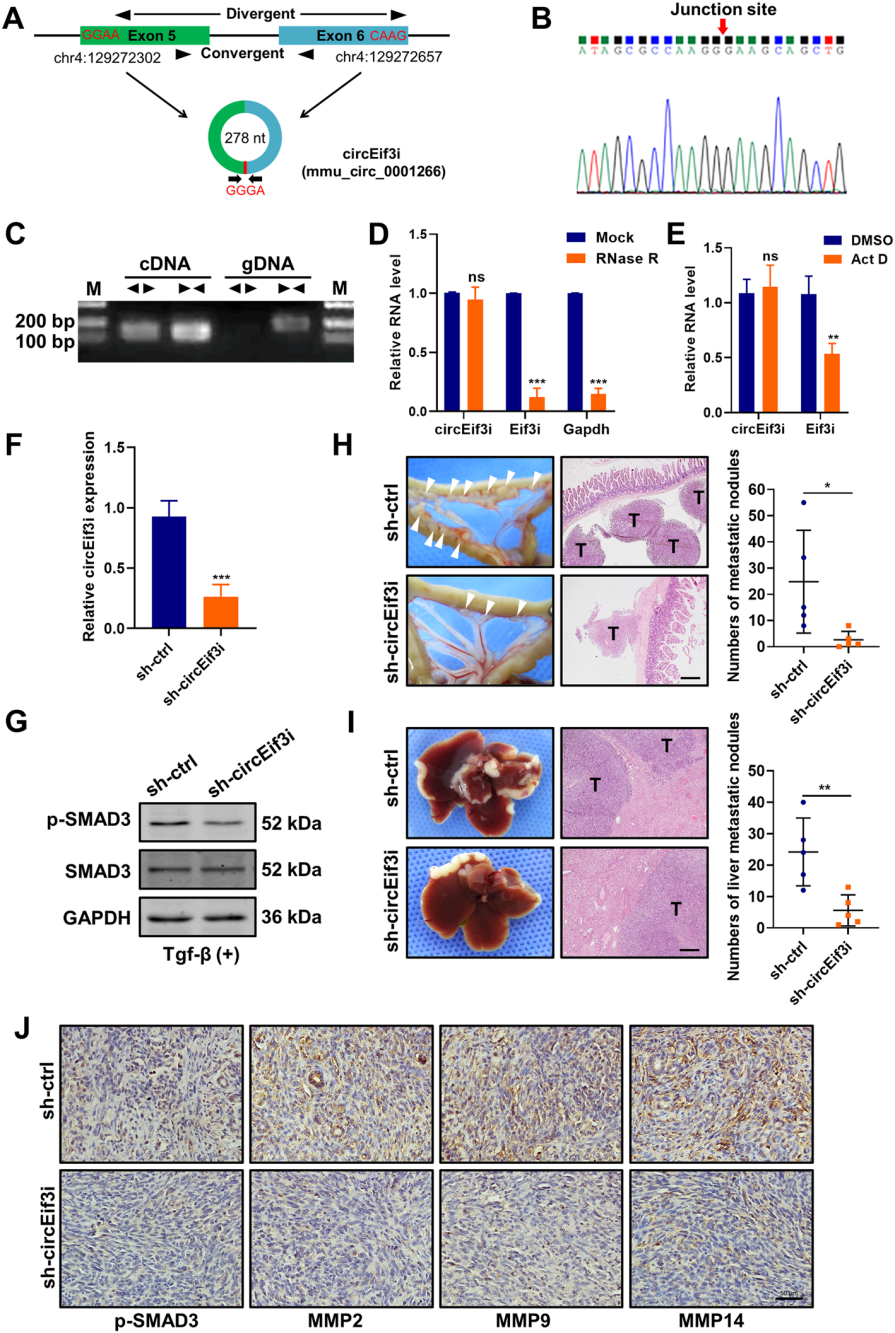
Homologous circEif3i in mouse performs similar physiological functions. **A** Schematic diagram of circEif3i generated from the Exon 5 and Exon 6 of Eif3i gene. **B** Sanger sequencing of head-to-tail splice junction in circEif3i. **C** The agarose gel electrophoresis was performed using the divergent primers and convergent primers to identify the existence of circEif3i. **D** qRT-PCR assay to determine circEif3i, Eif3i mRNA and Gapdh mRNA levels in samples after RNase R treatment. **E** qRT-PCR showed the stabilities of circEif3i and Eif3i mRNA in pancreatic cancer cell treated with 5 μg/ml actinomycin D (Act D) for 24 h. **F** qRT-PCR analyses of relative circEif3i in Panc02 cells transfected with sh-ctrl or sh-circEif3i. **G** Western blot showed the protein expression of p-SMAD3 (Ser423/425) and SMAD3 in Panc02 cells transfected with sh-ctrl or sh-circEif3i, with Tgf-β treatment (5 ng/ml, 15 min). **H** Representative images of the metastases (white arrows) of orthotopic models and HE staining of intestinal and mesentery metastatic nodules (Scale bar = 200 μm), and visible metastatic nodes were calculated (right panel). **I** Representative photographs of the liver metastasis model and HE staining of liver metastatic nodules with original magnification (Scale bar = 200 μm). The number of macroscopic metastases were calculated (right panel). **J** Representative images of immunohistochemistry analysis to measure the expression of p-SMAD3, MMP2, MMP9, and MMP14 in orthotopic tumours from negative control and circEif3i KD group (Scale bar = 50 μm). Data are shown as the mean ± SD of three replicates; *P < 0.05; **P < 0.01; ***P < 0.001; ns, not significant

Through the circBank database, we found a conserved circEIF3I equivalent in mice, mmu_circ_0001266, and we named it circEif3i (Fig. 8A). The sequences of circEif3i are highly similar to circEIF3I (Supplementary Table S6), indicating that their physiological function may be conserved. The head-to-tail splicing of circEif3i extracted from Panc02 was confirmed by Sanger sequencing (Fig. 8B). Divergent and convergent primers were designed, and agarose gel electrophoresis analysis of the PCR products from Panc02 showed that circEif3i was amplified only in cDNA by divergent primers, not in genomic DNA (Fig. 8C). Moreover, we verified that circEif3i was more stable and had a longer half-life than its linear form, as indicated via treatment with RNase R and actinomycin D (Fig. 8D, E). The shRNA targeting the back-spliced junction site of circEif3i appreciably downregulated circEif3i expression (Fig. 8F). Consistently, the protein levels of p-SMAD3, but not SMAD3, were decreased in Panc02 cells after circEif3 KD (sh-circEif3i) with Tgf-β treatment (5 ng/ml) (Fig. 8G). To further explore the biological functions of circEif3i in vivo, we established orthotopic and liver metastatic mouse models using stably transfected Panc02 cell lines. The results showed that fewer visible metastatic nodes in the intestine, mesentery and liver in the sh-circEif3i group than in the sh-ctrl group (Fig. 8H, I), while the orthotopic tumour volume was not significantly different (Fig. S8). In addition, an IHC assay with the transplanted tumours indicated high expression of p-SMAD3, MMP2, MMP9 and MMP14 in the control group (Fig. 8J). Taken together, these results indicate that circEif3i in mice promotes pancreatic cancer cell invasion and metastasis by regulating TGF-β signalling, similar to circEIF3I in humans.

## Discussion

Abundant evidence has revealed that circRNAs play a critical role in tumour progression [3, 5]. In this study, we first identified that circEIF3I, derived from the EIF3I gene, is highly expressed in PDAC tissues, and its expression is associated with metastasis and poor prognosis. Then, we confirmed that circEIF3I promoted tumour invasion and metastasis capacity. Mechanistically, circEIF3I directly bound to SMAD3 and promoted SMAD3 phosphorylation by facilitating the recruitment of SMAD3 to EEs. This recruitment was due to the function of circEIF3I as a protein scaffold, that simultaneously binds to SMAD3 and AP2A1, an endocytic-related protein enriched on EEs. The ternary complex formation of circEIF3I, SMAD3 and AP2A1 is crucial for circEIF3I/TGF-β signalling/MMP activation. Furthermore, we found that the suppression of circEif3i, a homologous circRNA of circEIF3I in mouse, also inhibited tumour metastasis by impairing SMAD3 phosphorylation. These results provide new insights into the function of circEIF3I in the progression of PDAC, and contribute to a better understanding of the oncogenic process.

Several circRNAs are involved in PDAC, including hsa_circ_0000977 [38], circPDE8A [39], circNEIL3 [40], and circNFIB1 [41], which play distinct roles in PDAC progression but all function as competing endogenous RNAs (ceRNAs). Notably, endogenous miRNA-target ratios determine the susceptibility to potential ceRNA competition, and a high number of additional targets are required to obtain measurable consequences [42], but most reported circRNAs carry only one binding site (such as hsa_circ_0000977, circNEIL3, and circNFIB1, as mentioned above); thus, the miRNA sponging effect of circRNAs has always been debated [43]. Lacking enough miRNA binding sites makes circEIF3I less likely to function as a miRNA sponge, and the predominantly cytosolic localization excludes its function in modulating gene transcription in the nucleus. On the other hand, increasing evidence demonstrates that circRNAs are scaffolds that facilitate interactions between RNA binding proteins or proteins that are routinely recognized without nucleotide-binding activity [3–6, 44, 45]. These circRNAs enable binding and biochemical reactions between proteins, or alter the subcellular localization of proteins, as we have proven by shown that circEIF3I activates SMAD3.

TGF-β signalling plays oncogenic roles during advanced stages of cancer by promoting the EMT, angiogenesis, immune evasion and metastasis [13]. The abundant evidence showing the multiple mechanisms that mediate EMT after TGF-β stimulation has led to efforts to identify chemical modifiers of this process, such as TGF-β ligand traps or TGF-β receptor kinase inhibitors, which might be transformed into clinically valid anti-metastatic drugs [46]. Unravelling the underlying mechanism by which circRNAs regulate the TGF-β pathway may provide a new direction for developing therapeutic agents. A recent study reported that circPTEN1 bound the MH2 domain of SMAD4 to impair the physical interaction of the circRNA with SMAD2/3, which inhibited the formation and nuclear translocation of SMAD complexes and subsequently inhibited the transcription of TGF-β induced EMT genes in colorectal cancer [47]. In contrast to the findings in colorectal cancer, our data revealed a novel mechanism by which circEIF3I promoted SMAD3 delivery to EEs by directly binding to the SMAD3 MH2 domain, eventually enhancing the phosphorylation of SMAD3 by facilitating the recruitment of SMAD3 to internalized TGFβRI. Although circEIF3I and circPTEN1 function in different ways, targeting circRNAs may be a novel weapon against aberrant TGF-β signalling activation in cancer.

We observed that circEIF3I-induced SMAD3 phosphorylation primarily on the endosomal membrane; however, circEIF3I was unable to interact with TGFβRI directly. These data indicate that there might be an "anchor" distributed on EEs that accelerates circEIF3I-bound SMAD3 delivery to TGFβRI. Using mass spectrometry and RNA pull-down assays, we identified AP2A1, a subunit of the AP2 complex, which plays an important role in clathrin-mediated endocytosis and EE formation [35, 36]. As we verified in our study, AP2A1 was specifically enriched on EEs and constituted the RNA–protein complex in combination with circEIF3I and SMAD3. In addition, knocking down AP2A1 severely impaired circEIF3I-induced SMAD3 phosphorylation, which further confirmed that AP2A1 functions as an anchor site to facilitate interactions between SMAD3 and TGFβRI on EEs by directly binding with circEIF3I. To our knowledge, this is the first report of circRNA-induced TGF-β pathway activation through circRNA-assisted delivery of SMAD3 to EEs. Moreover, given the abundant biological function of circRNAs, simply degrading circEIF3I using targeted agents might cause unexpected adverse effects, while the recognition of circEIF3I-specific binding sites in bridging both SMAD3 and AP2A1 potentially remedies these shortcomings. It should be noted that there are some limitations in our experiments; many proteins showed a clear preference for the RNA secondary structure, flanking nucleotide composition and multipartite sites [48]. However, the oligonucleotide probes used in RNA-EMSA do not guarantee the integrity of the secondary structure or flank sequences, nor the multipartite motifs of the entire circEIF3I. Therefore, the validation of key base sites showed certain uncertainty, and further in-depth and sophisticated studies are required to precisely locate the key nucleotides that bind to proteins.

It is conventionally believed that genes controlling important biological functions, processes, and structural elements are conserved throughout evolution [49]. Our study demonstrated that circEIF3I/circEif3i promotes human/mouse pancreatic cancer metastasis by regulating the TGF-β signalling pathway, which verifies that this circRNA is evolutionarily conserved [50].

## Conclusion

**Fig. 9:**
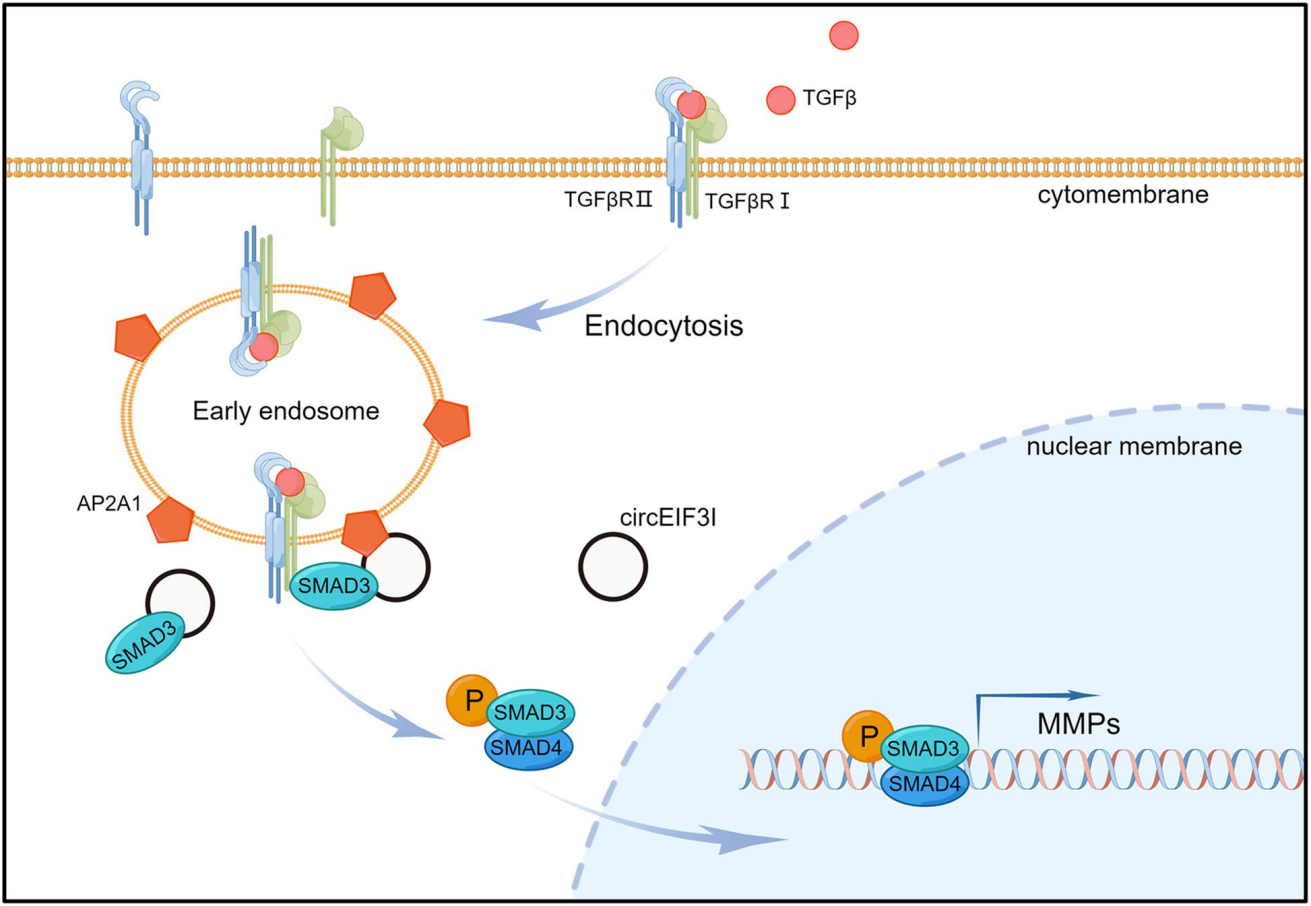
Proposed model of circEIF3I functions in malignant progression of PDAC

In conclusion, our study demonstrated that circEIF3I was highly correlated with the TNM stage and poor prognosis in pancreatic cancer. circEIF3I functions as a molecular scaffold that binds with MH2 domain of SMAD3 and AP2A1 to form a ternary complex that facilitates SMAD3 recruitment to TGFβRI on early endosomes and SMAD3 phosphorylation Fig. 9. Moreover, circEif3i, the homologous circRNA in mice, promotes tumour metastasis by regulating TGF-β signalling. Together, these data show that circEIF3I may function as both a potential prognostic biomarker and therapeutic target in PDAC.

### Supplementary Information

**Additional file 1: Fig. S1.** qRT-PCR analysis of 10 circRNAs in 30 pairs of human PDAC tissues and adjacent normal tissues. Data are shown as the means ± SD; **p < 0.01; ns, not significant.

**Additional file 2: Fig. S2.** (A) Relative expression of EIF3I mRNA in PANC-1 and BxPC-3 cells after circEIF3I siRNA transfection. (B and C) Relative expression of circEIF3I (B) and EIF3I mRNA (C) in PANC-1 and BxPC-3 cells after sh-circEIF3I transfection. (D, E) Transwell assay demonstrated the effect of circEIF3I knockdown (KD) on PDAC cells migration (D) and invasion (E) ability via sh-circEIF3I (Scale bar =100μm). (F and L) Representative images of the metastases (white arrows) of orthotopic xenograft model established using cells with knockdown (F) or overexpression (L) of circEIF3I, and HE staining of intestinal and mesentery metastatic nodules (Scale bar = 200μm), and visible metastatic nodes were calculated (right panel). (G) Representative images of liver metastasis model established using cells transfected with sh-circEIF3I-1, and HE staining of liver metastatic nodules with original magnification (Scale bar = 200μm). The number of macroscopic metastases were calculated (right panel). (H and M) Representative images of orthotopic xenograft model established using cells with circEIF3I KD (H) or overexpression (M), and tumours volume were evaluated (right panel). (I) Relative expression of circEIF3I in pancreatic cancer cells after circEIF3I transfection. (J and K) Transwell assay demonstrated the effect of circEIF3I overexpression on PDAC cells migration (J) and invasion (K) ability (Scale bar =100μm). Data are shown as the mean ± SD of three replicates; *P < 0.05; **P < 0.01; ***P < 0.001; ns, not significant.

**Additional file 3: Fig. S3.** (A and B) The circEIF3I-induced migration and invasion ability were restrained by treating with marimastat (10μM) (Scale bar =100μm). Data are shown as the means ± SD; ***P < 0.001; ns, not significant.

**Additional file 4: Fig. S4.** (A) The number of putative microRNA binding sites in circEIF3I through CircInteractome. (B) qRT-PCR analyses of relative expression of hsa-miR-149-5p, hsa-miR-361-3p, hsa-miR-615-5p and hsa-miR-616-3p in PANC-1 and BxPC-3 cells after circEIF3I KD. (C) Relative expression of EIF3I protein in PANC-1 and BxPC-3 cells after circEIF3I KD. (D) Relative expression of circEIF3I in cells transfected with MS2-circEIF3I plasmid. (E) Sanger sequencing of the junction site in MS2-circEIF3I. (F) Quantification of RNAs by qRT-PCR after MS2-GST fusion protein capture. (G) Proteins precipitated by RNA pull-down assay were detected by silver staining. (H) Schematic illustrating cyclization of circEIF3I in vitro (left). circEIF3I synthesized in vitro was validated through RNAse R digestion experiments (right). (I) Relative expression of circEIF3I and EIF3I mRNA captured by specific circEIF3I probe and negative control probe. (J) Representative images of immunohistochemistry (IHC) analysis to detect the expression of p-SMAD3 in xenograft tumours (Scale bar = 50μm). (K) Western blot showed the expression of MMP2, MMP9 (10ng/ml PMA) and MMP14 in PANC-1 and BxPC-3 cells transfected with sh-ctrl or sh-circEIF3I-2, with or without the treatment of SIS3 (15μM). Data are shown as the means ± SD; **p < 0.01; ***P < 0.001; ns, not significant.

**Additional file 5: Fig. S5.** (A) Western blot showed the expression of TGFβR II and TGFβR I in PANC-1 and BxPC-3 cells transfected with sh-ctrl or sh-circEIF3I-2. (B and C) Western blot showed the p-SMAD3 expression (5ng/ml TGF-β, 15min) in cells transfected with vector or circEIF3I, with or without the endocytosis blocked by potassium depletion or Dyngo-4a (30μM). (D) Representative IF images identified the colocalization of SMAD3 and EEA1 in PANC-1 cells transfected with vector or circEIF3I (5ng/ml TGF-β, 15min). The red indicated SMAD3, the green indicated the EEA1, the blue (DAPI) indicated the nucleus (Scale bar = 10μm). (E)The fluorescence intensity of regions of interest (ROI) was quantified along with the indicated white dashed using ImageJ software.

**Additional file 6: Fig. S6.** (A) RNA pull-down and western blot assays showed circEIF3I cannot bind TGFβR I directly. (B) The bindings between circEIF3I and ANXA2, PACSIN2 or TRIP10 are non-specific. (C) Representative IF images identified the enrichment of AP2A1 on early endosomes (EEs) in BxPC-3 cells. The red indicated AP2A1, the green indicated the EEA1 (marker of EEs), the blue (DAPI) indicated the nucleus (Scale bar = 10μm). (D and E) Graphical representation of three-dimensional structures of the docking models of circEIF3I with SMAD3 (D) and AP2A1 (E) respectively. (F) Graphical representation of three-dimensional structures of the docking models of circEIF3I with both SMAD3 and AP2A1. (G and H) circEIF3I mutations (blue labelling) that selectively block its binding with SMAD3 (G) or AP2A1 (H). (I) RNA pull-down and western blot identified SMAD3 and AP2A1 precipitated by circEIF3I probe in circEIF3I KD cells, with the ectopic expression of circEIF3I-WT, circ-mut-SMAD3 and circ-mut-AP2A1 respectively. (J) The binding of SMAD3 and TGFβR I in BxPC-3 transfected with sh-ctrl or sh-circEIF3I-2, with the ectopic expression of circEIF3I-WT, circ-mut-SMAD3 and circ-mut-AP2A1 respectively in circEIF3I KD groups (5ng/ml TGF-β, 15min). (K and L) Rescue experiments of Transwell assay in cells transfected with sh-ctrl or sh-circEIF3I-2, with the ectopic expression of circEIF3I-WT, circ-mut-SMAD3 and circ-mut-AP2A1 respectively in circEIF3I KD groups. Data are shown as the mean ± SD; **P < 0.01; ***P < 0.001; ns, not significant.

**Additional file 7: Fig. S7.** (A) The p-SMAD3 expression (5ng/ml TGF-β, 15min) in cells transfected with sh-ctrl or sh-circEIF3I-2, with the ectopic expression of circEIF3I-WT (with or without si-AP2A1), circ-mut-SMAD3 and circ-mut-AP2A1 respectively in circEIF3I KD groups. (B) The MMP2, MMP9 (10 ng/ml PMA) and MMP14 expression in cells transfected with sh-ctrl or sh-circEIF3I-2, with the ectopic expression of circEIF3I-WT (with or without SIS3), circ-mut-SMAD3 and circ-mut-AP2A1 respectively in circEIF3I KD groups (5ng/ml TGF-β, 24h). (C) Schematic diagram of RNA-EMSA probes. (D) RNA-EMSA determined the specific binding between AP2A1 and biotin-labelled Probe2 and Probe3 of circEIF3I, with AP2A1 antibody incubation (super shift band). (E) SMAD3 binding motif and AP2A1 binding motif were identified in Probe3 and Probe2, respectively. (F) The amino acid sequence of HA-MH2-Mut. (G) RIP assay demonstrated the HA-MH2-WT and HA-MH2-Mut binding capacity with circEIF3I. (H) RNA pull-down and western blot identified precipitated HA-MH2-WT and HA-MH2-Mut by circEIF3I probe. Data are shown as the mean ± SD; **P < 0.01.

**Additional file 8: Fig. S8.** Representative images of orthotopic models from two groups (left panel) and tumours volume were evaluated (right panel). ns, not significant.

**Additional file 9: Supplementary Table S1.** Clinical correlation between circEIF3I expression and clinical pathological characteristics in PDAC patients.

**Additional file 10: Supplementary Table S2.** The target sequences of siRNAs used in transfection.

**Additional file 11: Supplementary Table S3.** MS2-TRAP assay mass spectrometry results.

**Additional file 12: Supplementary Table S4.** The interacting atoms of the two nodes involved (residues/ligands) with relative distances less than 3.5Å.

**Additional file 13: Supplementary Table S5.** The identified motifs of SMAD3 and AP2A1 using RIP-seq.

**Additional file 14: Supplementary Table S6.** Alignment of circEif3i (mmu_circ_0001266) and circEIF3I (hsa_circ_0011385).

**Additional file 15: Supplementary Table S7.** Sequences of primers used in qRT-PCR.

**Additional file 16: Supplementary Table S8.** The list of antibodies used in this study.

**Additional file 17: Supplementary Methods.** The methods and materials used in this research.

## Data Availability

The datasets used and/or analysed during the current study are available from the corresponding author on reasonable request.

## Abbreviations

PDAC: Pancreatic ductal adenocarcinoma
circRNAs: Circular RNAs
miRNA: MicroRNA
GF-β: Transforming growth factor-β
EMT: Epithelial-to-mesenchymal transition
MMP: Matrix metalloprotease
SMAD: Mothers against decapentaplegic homolog
TGFβRI: TGF-β receptor I
EEs: Early endosomes
ISH: In situ hybridization
FISH: Fluorescence in situ hybridization
siRNAs: Small-interfering RNAs
KD: Knockdown
IHC: Immunohistochemistry
ORF: Open reading frame
IRES: Internal ribosome entry site
MS2-TRAP: MS2-tagged RNA affinity purification
MS: Mass spectrometry
RIP: RNA immunoprecipitation
IF: Immunofluorescence
H-score: Histochemistry score
TGFβRII: TGF-β receptor II
co-IP: Co-immunoprecipitation
ceRNA: Competing endogenous RNAs
