# Supplementary figures and images for: circEIF3I facilitates the recruitment of SMAD3 to early endosomes to promote TGF-β signalling pathway-mediated activation of MMPs in pancreatic cancer
Source: Mol Cancer. 2023 Sep 9;22:152. doi: 10.1186/s12943-023-01847-2 (PMC10492306; doi:10.1186/s12943-023-01847-2)

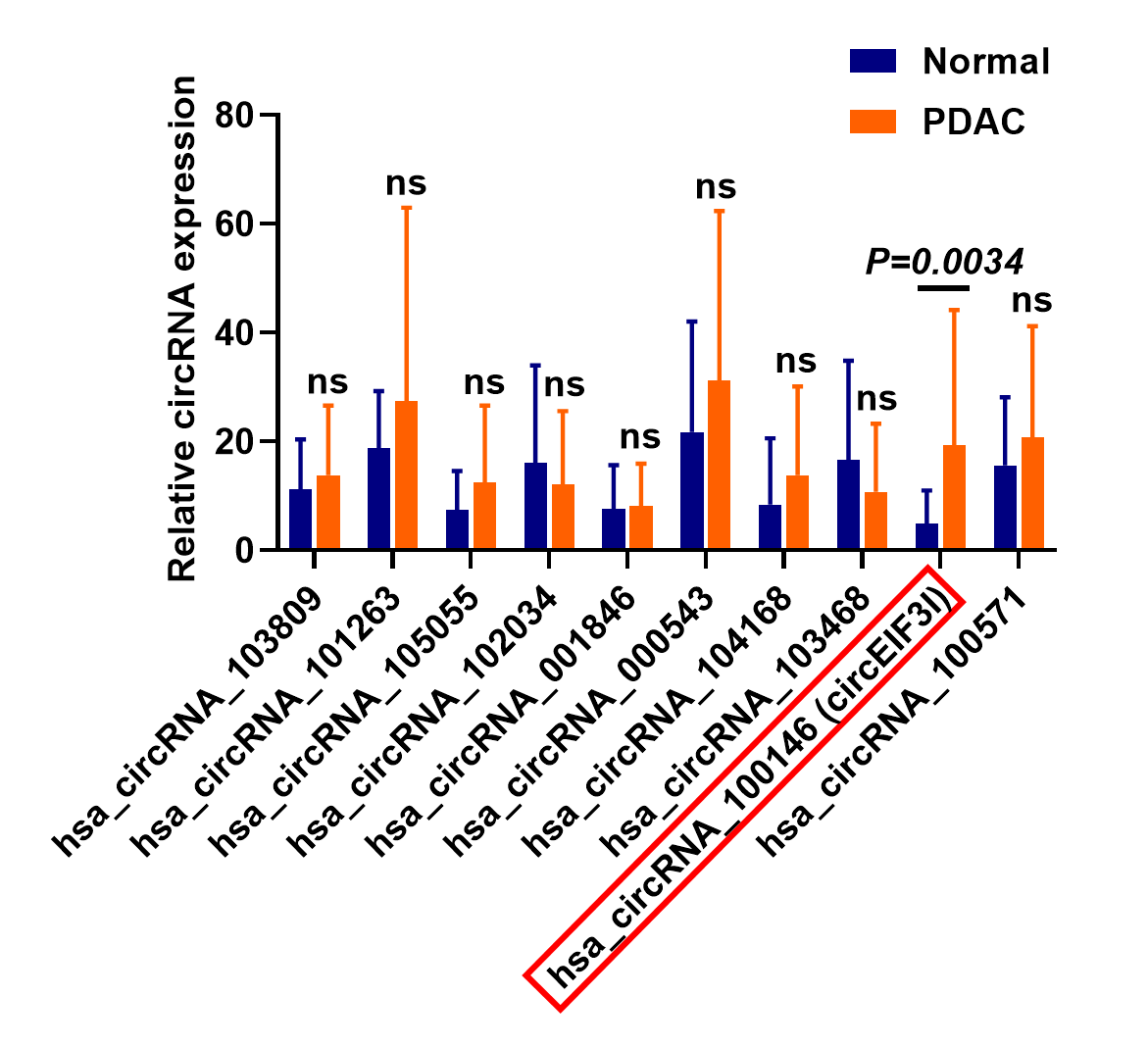

Supplement: Supplementary file 1 — Additional file 1: Fig. S1. qRT-PCR analysis of 10 circRNAs in 30 pairs of human PDAC tissues and adjacent normal tissues. Data are shown as the means ± SD; **p < 0.01; ns, not significant. [file 12943_2023_1847_MOESM1_ESM.tif]

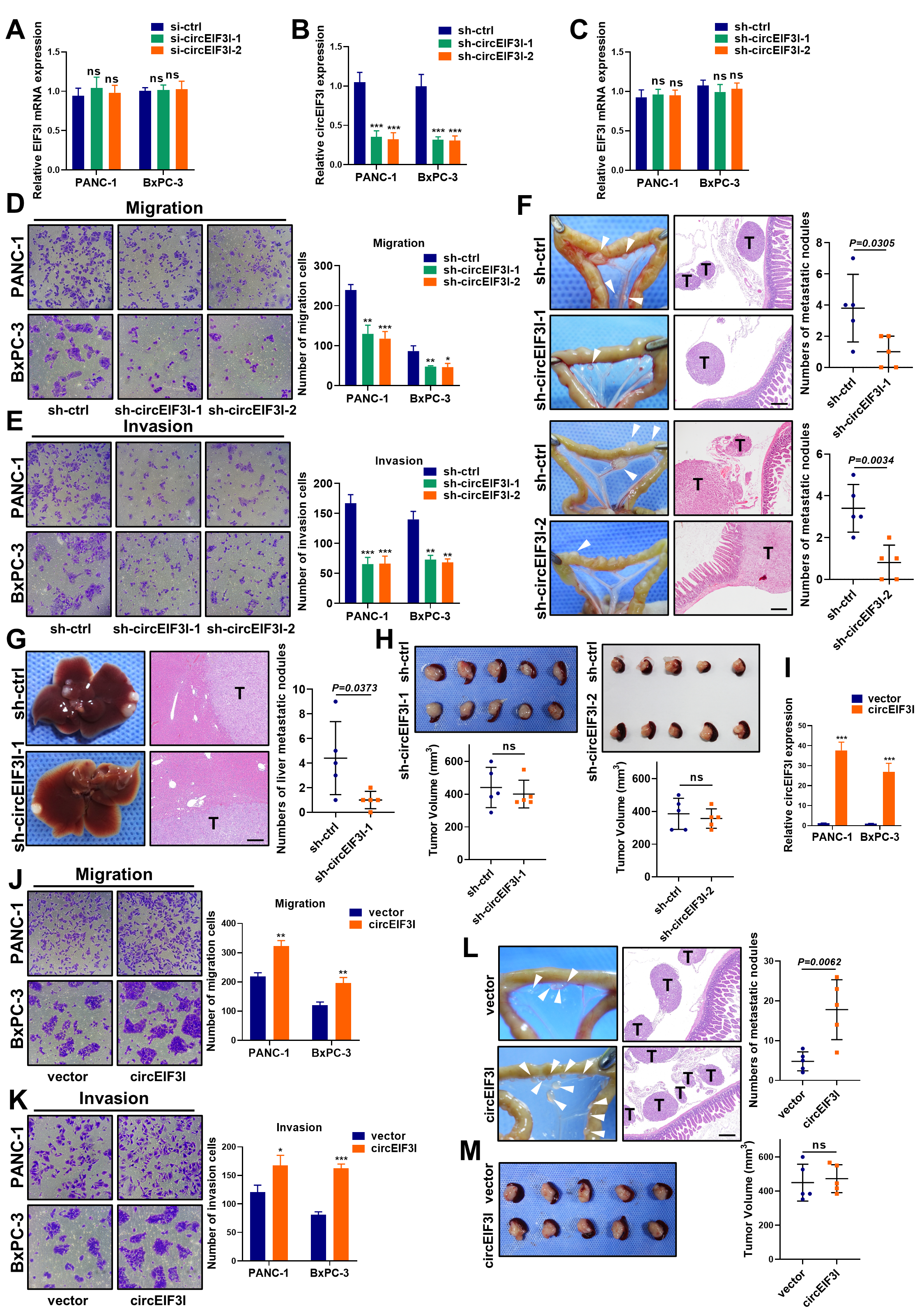

Supplement: Supplementary file 2 — Additional file 2: Fig. S2. (A) Relative expression of EIF3I mRNA in PANC-1 and BxPC-3 cells after circEIF3I siRNA transfection. (B and C) Relative expression of circEIF3I (B) and EIF3I mRNA (C) in PANC-1 and BxPC-3 cells after sh-circEIF3I transfection. (D, E) Transwell assay demonstrated the effect of circEIF3I knockdown (KD) on PDAC cells migration (D) and invasion (E) ability via sh-circEIF3I (Scale bar =100μm). (F and L) Representative images of the metastases (white arrows) of orthotopic xenograft model established using cells with knockdown (F) or overexpression (L) of circEIF3I, and HE staining of intestinal and mesentery metastatic nodules (Scale bar = 200μm), and visible metastatic nodes were calculated (right panel). (G) Representative images of liver metastasis model established using cells transfected with sh-circEIF3I-1, and HE staining of liver metastatic nodules with original magnification (Scale bar = 200μm). The number of macroscopic metastases were calculated (right panel). (H and M) Representative images of orthotopic xenograft model established using cells with circEIF3I KD (H) or overexpression (M), and tumours volume were evaluated (right panel). (I) Relative expression of circEIF3I in pancreatic cancer cells after circEIF3I transfection. (J and K) Transwell assay demonstrated the effect of circEIF3I overexpression on PDAC cells migration (J) and invasion (K) ability (Scale bar =100μm). Data are shown as the mean ± SD of three replicates; *P < 0.05; **P < 0.01; ***P < 0.001; ns, not significant. [file 12943_2023_1847_MOESM2_ESM.tif]

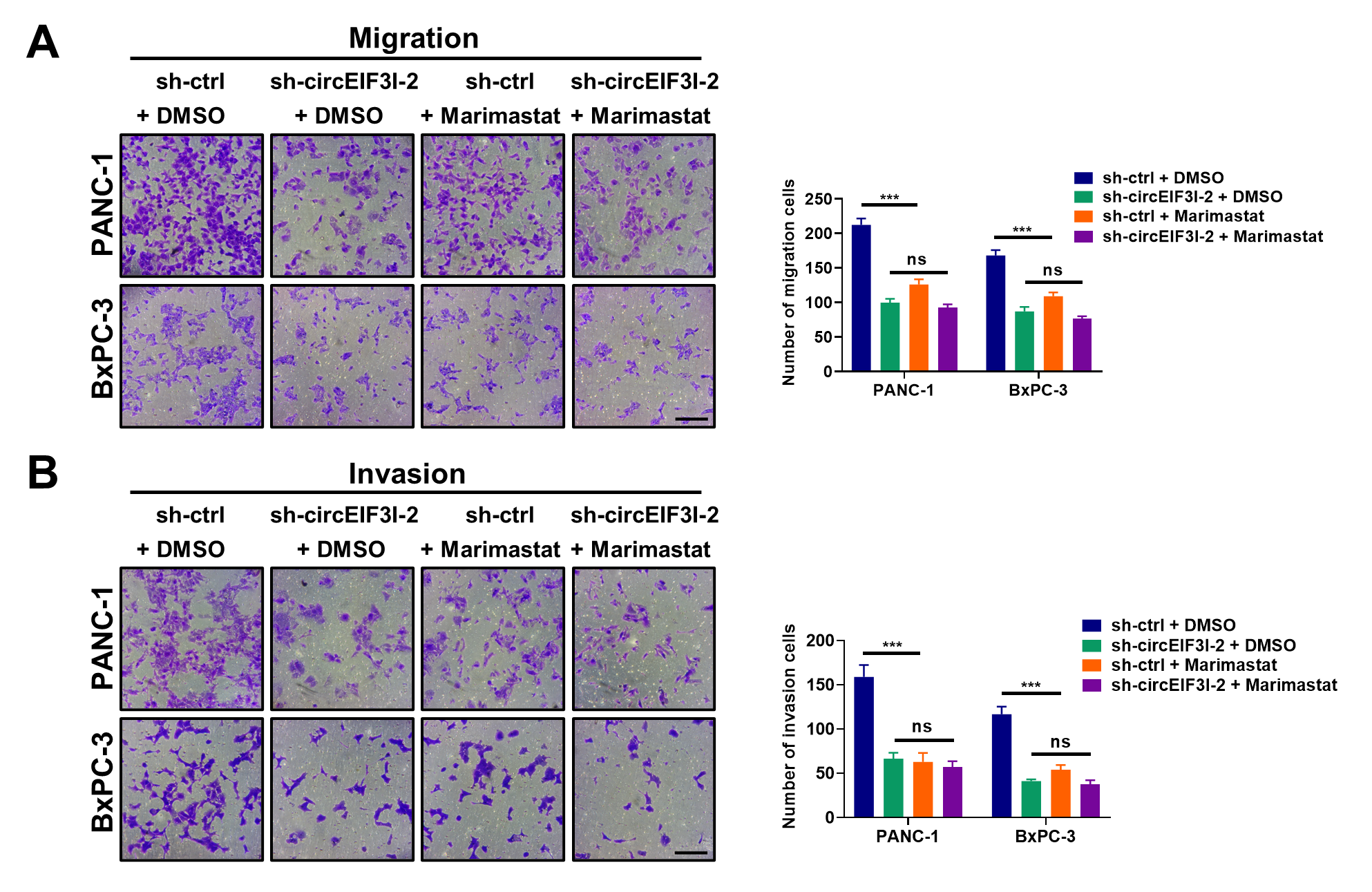

Supplement: Supplementary file 3 — Additional file 3: Fig. S3. (A and B) The circEIF3I-induced migration and invasion ability were restrained by treating with marimastat (10μM) (Scale bar =100μm). Data are shown as the means ± SD; ***P < 0.001; ns, not significant. [file 12943_2023_1847_MOESM3_ESM.tif]

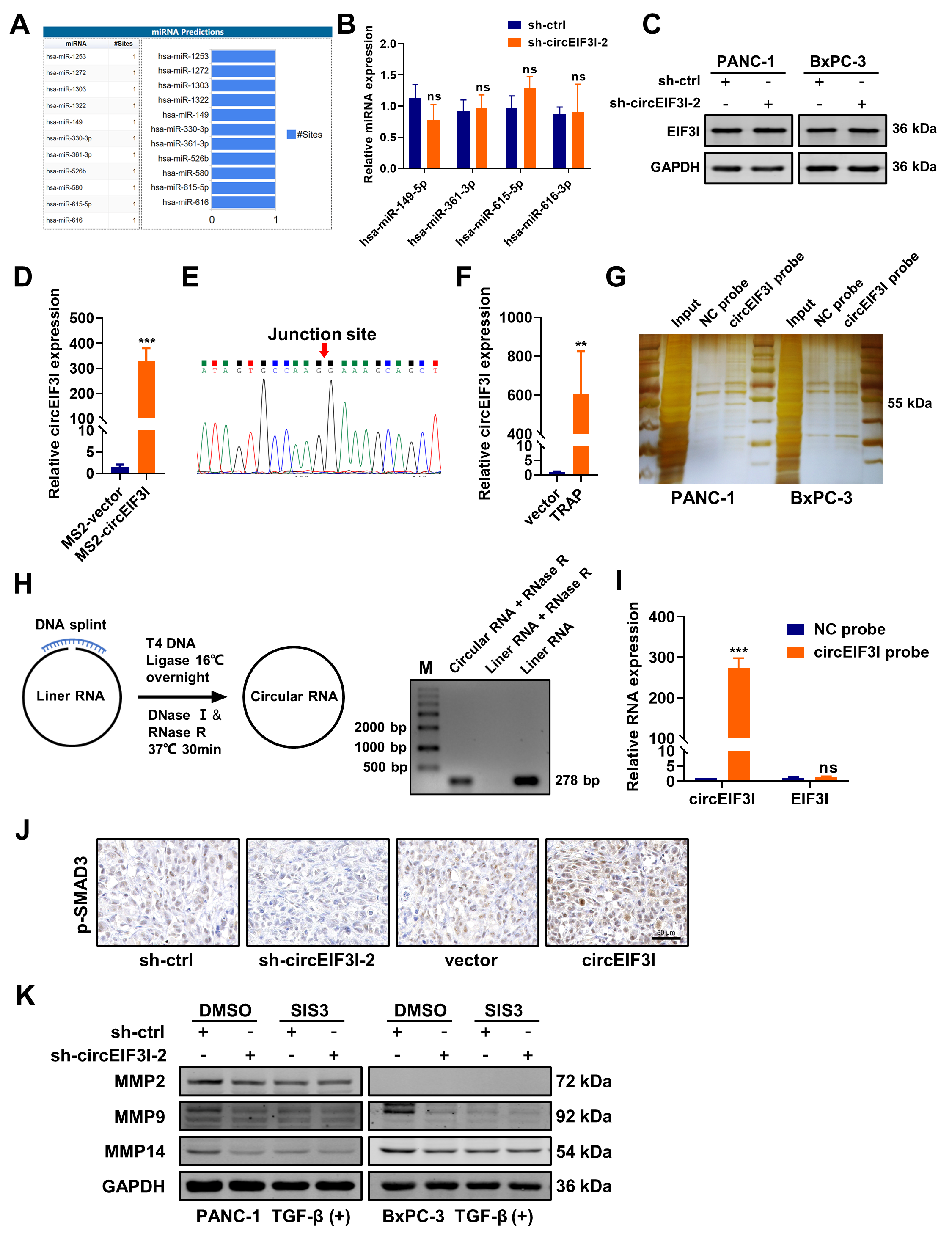

Supplement: Supplementary file 4 — Additional file 4: Fig. S4. (A) The number of putative microRNA binding sites in circEIF3I through CircInteractome. (B) qRT-PCR analyses of relative expression of hsa-miR-149-5p, hsa-miR-361-3p, hsa-miR-615-5p and hsa-miR-616-3p in PANC-1 and BxPC-3 cells after circEIF3I KD. (C) Relative expression of EIF3I protein in PANC-1 and BxPC-3 cells after circEIF3I KD. (D) Relative expression of circEIF3I in cells transfected with MS2-circEIF3I plasmid. (E) Sanger sequencing of the junction site in MS2-circEIF3I. (F) Quantification of RNAs by qRT-PCR after MS2-GST fusion protein capture. (G) Proteins precipitated by RNA pull-down assay were detected by silver staining. (H) Schematic illustrating cyclization of circEIF3I in vitro (left). circEIF3I synthesized in vitro was validated through RNAse R digestion experiments (right). (I) Relative expression of circEIF3I and EIF3I mRNA captured by specific circEIF3I probe and negative control probe. (J) Representative images of immunohistochemistry (IHC) analysis to detect the expression of p-SMAD3 in xenograft tumours (Scale bar = 50μm). (K) Western blot showed the expression of MMP2, MMP9 (10ng/ml PMA) and MMP14 in PANC-1 and BxPC-3 cells transfected with sh-ctrl or sh-circEIF3I-2, with or without the treatment of SIS3 (15μM). Data are shown as the means ± SD; **p < 0.01; ***P < 0.001; ns, not significant. [file 12943_2023_1847_MOESM4_ESM.tif]

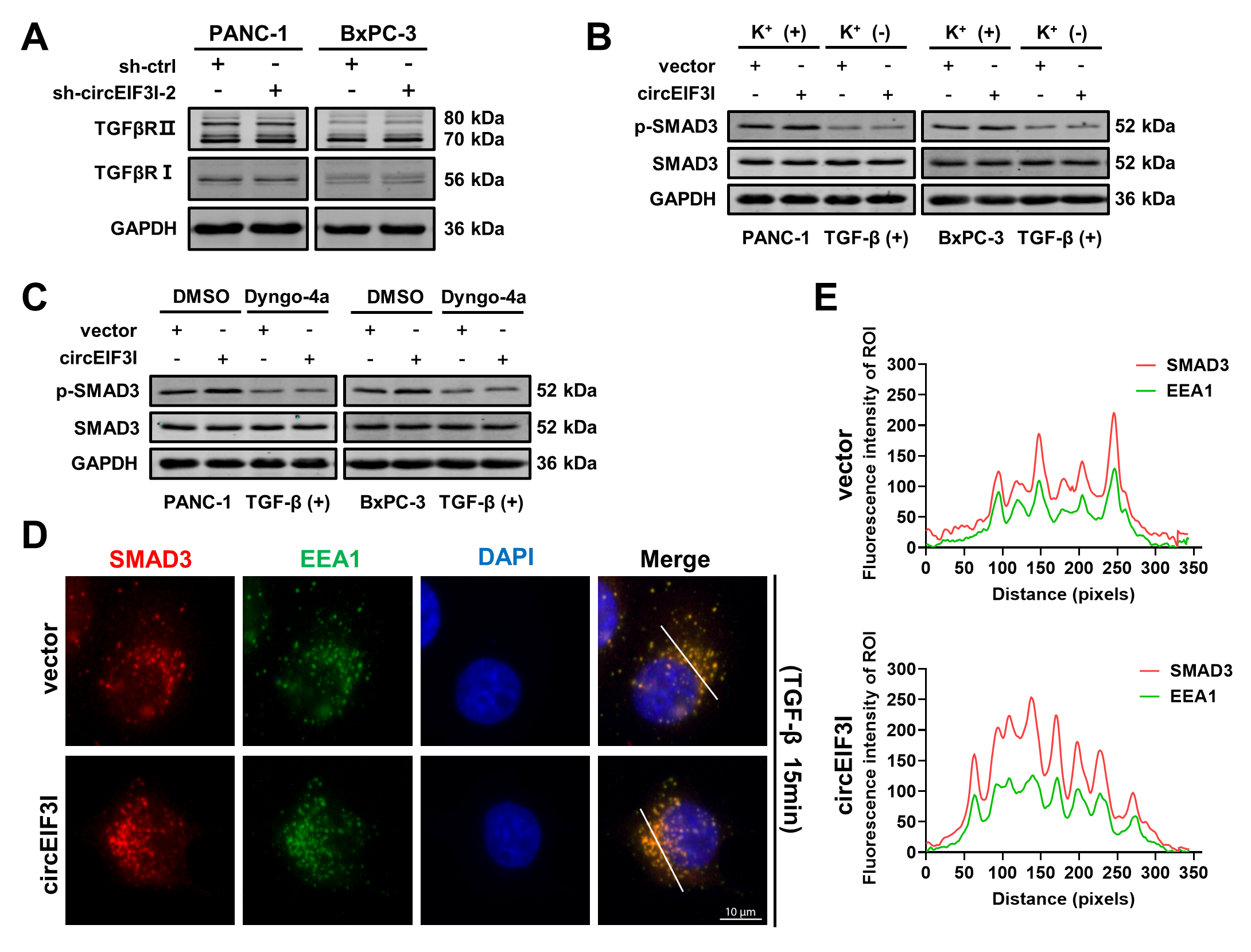

Supplement: Supplementary file 5 — Additional file 5: Fig. S5. (A) Western blot showed the expression of TGFβR II and TGFβR I in PANC-1 and BxPC-3 cells transfected with sh-ctrl or sh-circEIF3I-2. (B and C) Western blot showed the p-SMAD3 expression (5ng/ml TGF-β, 15min) in cells transfected with vector or circEIF3I, with or without the endocytosis blocked by potassium depletion or Dyngo-4a (30μM). (D) Representative IF images identified the colocalization of SMAD3 and EEA1 in PANC-1 cells transfected with vector or circEIF3I (5ng/ml TGF-β, 15min). The red indicated SMAD3, the green indicated the EEA1, the blue (DAPI) indicated the nucleus (Scale bar = 10μm). (E)The fluorescence intensity of regions of interest (ROI) was quantified along with the indicated white dashed using ImageJ software. [file 12943_2023_1847_MOESM5_ESM.tif]

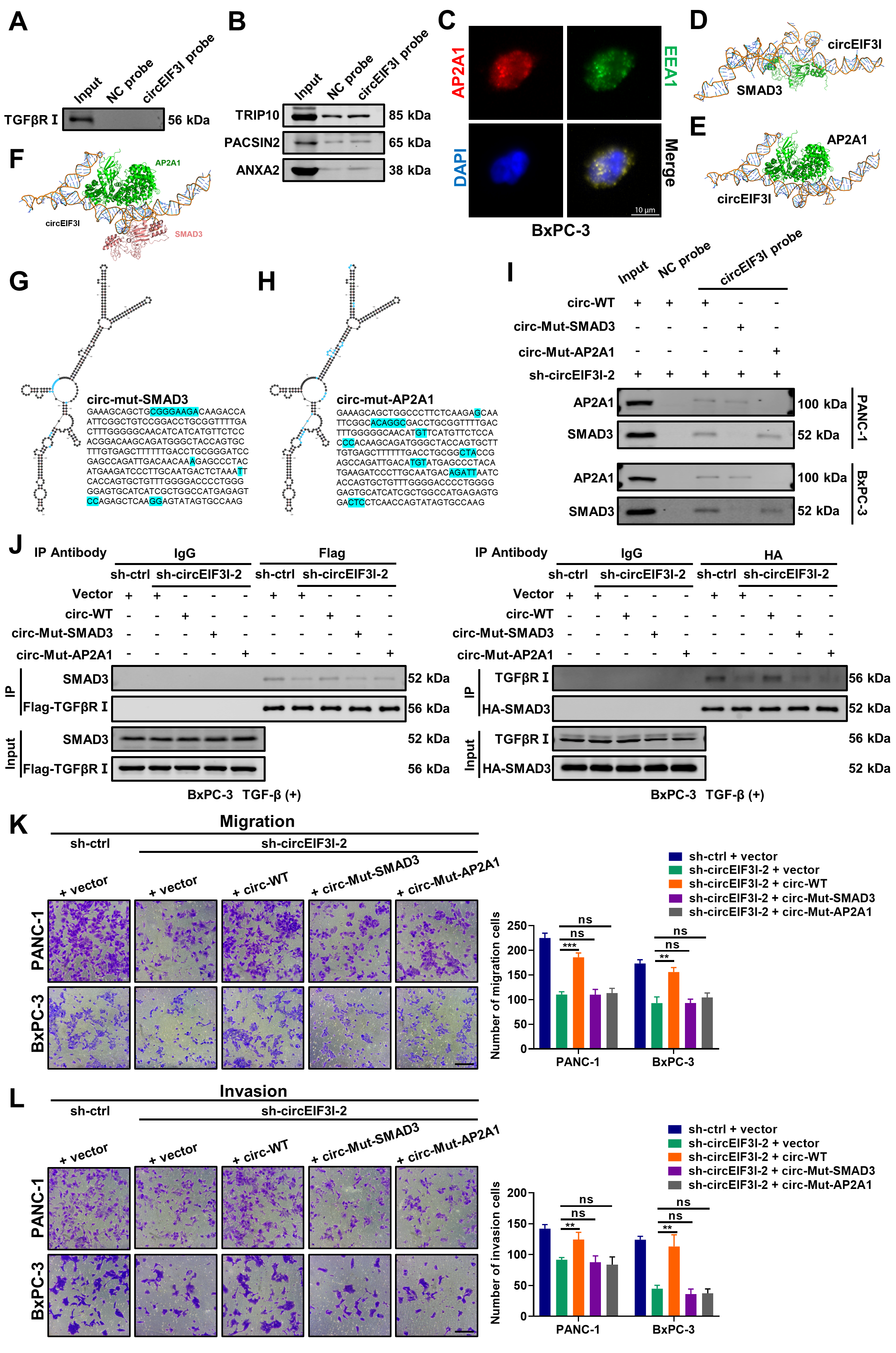

Supplement: Supplementary file 6 — Additional file 6: Fig. S6. (A) RNA pull-down and western blot assays showed circEIF3I cannot bind TGFβR I directly. (B) The bindings between circEIF3I and ANXA2, PACSIN2 or TRIP10 are non-specific. (C) Representative IF images identified the enrichment of AP2A1 on early endosomes (EEs) in BxPC-3 cells. The red indicated AP2A1, the green indicated the EEA1 (marker of EEs), the blue (DAPI) indicated the nucleus (Scale bar = 10μm). (D and E) Graphical representation of three-dimensional structures of the docking models of circEIF3I with SMAD3 (D) and AP2A1 (E) respectively. (F) Graphical representation of three-dimensional structures of the docking models of circEIF3I with both SMAD3 and AP2A1. (G and H) circEIF3I mutations (blue labelling) that selectively block its binding with SMAD3 (G) or AP2A1 (H). (I) RNA pull-down and western blot identified SMAD3 and AP2A1 precipitated by circEIF3I probe in circEIF3I KD cells, with the ectopic expression of circEIF3I-WT, circ-mut-SMAD3 and circ-mut-AP2A1 respectively. (J) The binding of SMAD3 and TGFβR I in BxPC-3 transfected with sh-ctrl or sh-circEIF3I-2, with the ectopic expression of circEIF3I-WT, circ-mut-SMAD3 and circ-mut-AP2A1 respectively in circEIF3I KD groups (5ng/ml TGF-β, 15min). (K and L) Rescue experiments of Transwell assay in cells transfected with sh-ctrl or sh-circEIF3I-2, with the ectopic expression of circEIF3I-WT, circ-mut-SMAD3 and circ-mut-AP2A1 respectively in circEIF3I KD groups. Data are shown as the mean ± SD; **P < 0.01; ***P < 0.001; ns, not significant. [file 12943_2023_1847_MOESM6_ESM.tif]

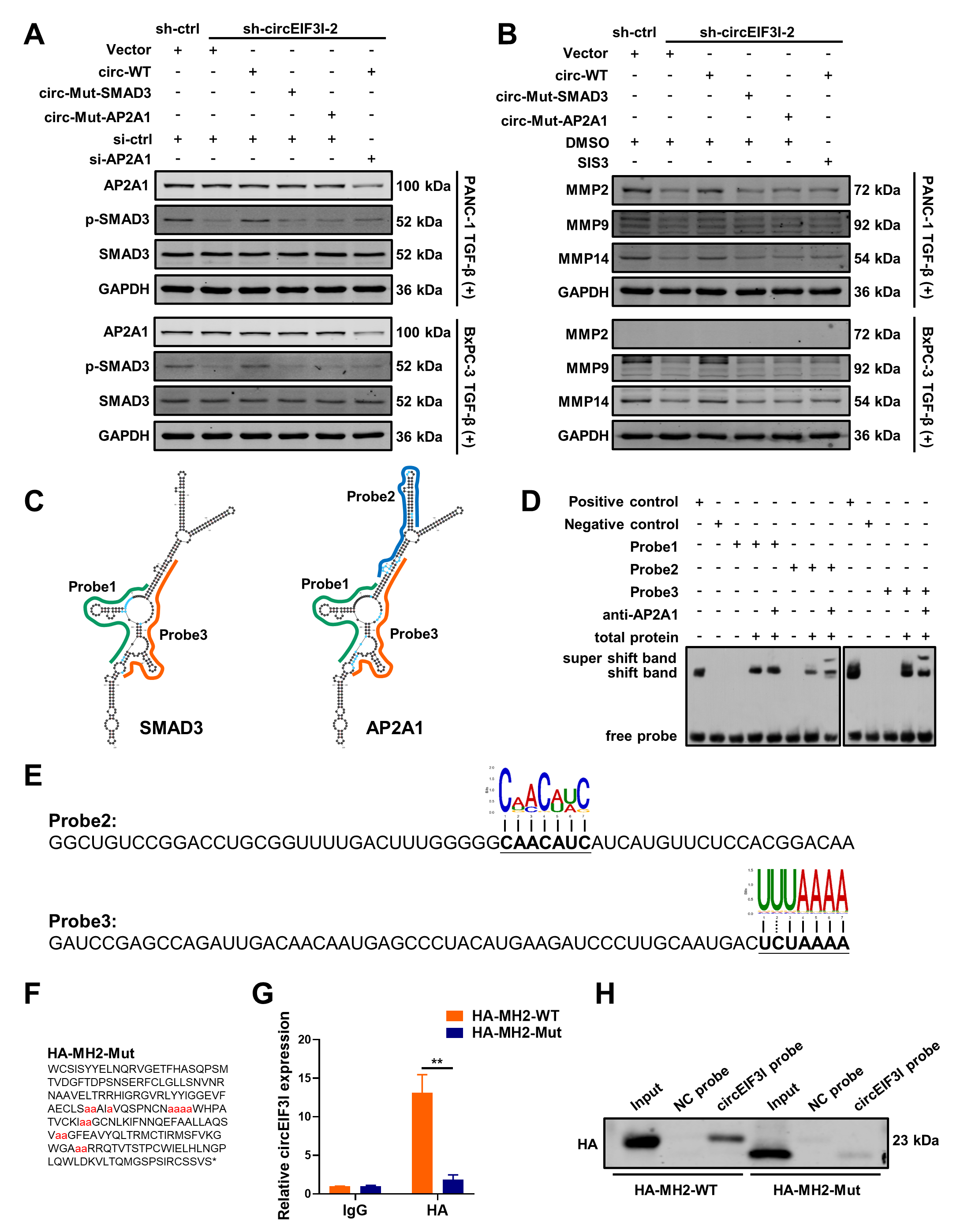

Supplement: Supplementary file 7 — Additional file 7: Fig. S7. (A) The p-SMAD3 expression (5ng/ml TGF-β, 15min) in cells transfected with sh-ctrl or sh-circEIF3I-2, with the ectopic expression of circEIF3I-WT (with or without si-AP2A1), circ-mut-SMAD3 and circ-mut-AP2A1 respectively in circEIF3I KD groups. (B) The MMP2, MMP9 (10 ng/ml PMA) and MMP14 expression in cells transfected with sh-ctrl or sh-circEIF3I-2, with the ectopic expression of circEIF3I-WT (with or without SIS3), circ-mut-SMAD3 and circ-mut-AP2A1 respectively in circEIF3I KD groups (5ng/ml TGF-β, 24h). (C) Schematic diagram of RNA-EMSA probes. (D) RNA-EMSA determined the specific binding between AP2A1 and biotin-labelled Probe2 and Probe3 of circEIF3I, with AP2A1 antibody incubation (super shift band). (E) SMAD3 binding motif and AP2A1 binding motif were identified in Probe3 and Probe2, respectively. (F) The amino acid sequence of HA-MH2-Mut. (G) RIP assay demonstrated the HA-MH2-WT and HA-MH2-Mut binding capacity with circEIF3I. (H) RNA pull-down and western blot identified precipitated HA-MH2-WT and HA-MH2-Mut by circEIF3I probe. Data are shown as the mean ± SD; **P < 0.01. [file 12943_2023_1847_MOESM7_ESM.tif]

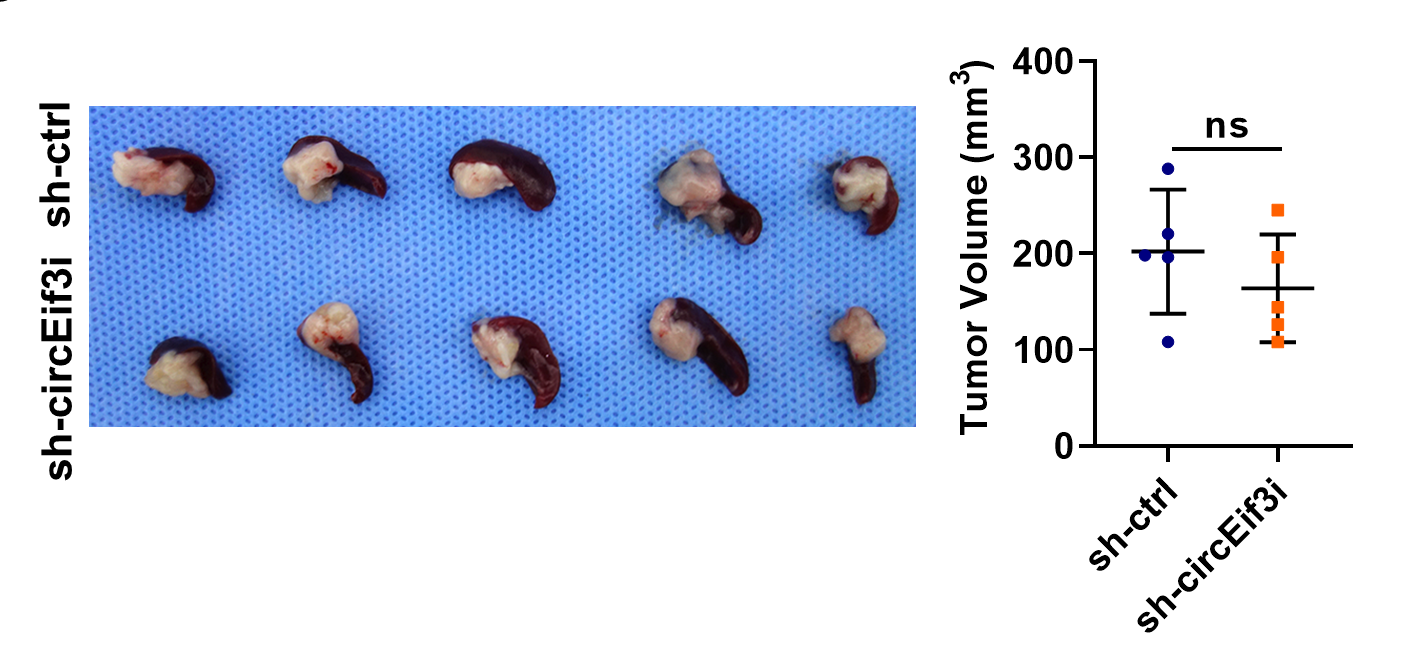

Supplement: Supplementary file 8 — Additional file 8: Fig. S8. Representative images of orthotopic models from two groups (left panel) and tumours volume were evaluated (right panel). ns, not significant. [file 12943_2023_1847_MOESM8_ESM.tif]
