# Supplementary material for: circEIF3I facilitates the recruitment of SMAD3 to early endosomes to promote TGF-β signalling pathway-mediated activation of MMPs in pancreatic cancer
Source: Mol Cancer. 2023 Sep 9;22:152. doi: 10.1186/s12943-023-01847-2 (PMC10492306; doi:10.1186/s12943-023-01847-2)
Supplement: Supplementary file 9 — Additional file 9: Supplementary Table S1. Clinical correlation between circEIF3I expression and clinical pathological characteristics in PDAC patients. [file 12943_2023_1847_MOESM9_ESM.docx]

**Table S1. Clinical correlation between circEIF3I expression and clinical pathological characteristics in PDAC patients.**

| **Clinical characteristics** | **Total** | **circEIF3I expression** | | ***P* value** |
| --- | --- | --- | --- | --- |
|  |  | **Low** | **High** |  |
| Age (years) |  |  |  |  |
| ＜60 | 38 | 22 | 16 | 0.263 |
| ≥60 | 42 | 18 | 24 |  |
| Gender |  |  |  |  |
| Male | 49 | 13 | 22 | 0.071 |
| Female | 31 | 27 | 18 |  |
| TNM stage |  |  |  |  |
| I+IIa | 48 | 31 | 17 | 0.003* |
| IIb+III | 32 | 9 | 23 |  |
| Nodal metastasis |  |  |  | 0.003* |
| Yes | 32 | 9 | 23 |  |
| No | 48 | 31 | 17 |  |
