# Supplementary material for: circEIF3I facilitates the recruitment of SMAD3 to early endosomes to promote TGF-β signalling pathway-mediated activation of MMPs in pancreatic cancer
Source: Mol Cancer. 2023 Sep 9;22:152. doi: 10.1186/s12943-023-01847-2 (PMC10492306; doi:10.1186/s12943-023-01847-2)
Supplement: Supplementary file 10 — Additional file 10: Supplementary Table S2. The target sequences of siRNAs used in transfection. [file 12943_2023_1847_MOESM10_ESM.docx]

**Table S2. The target sequences of siRNAs used in transfection.**

| Product Number | Product Name | Target Sequence |
| --- | --- | --- |
| siN0000001-1-5 | siR-Ribo™  Negative Control | N/A |
| siG161020040409 | si-hsa_circ_0011385_1 | CAGTATAGTGCCAAGGAAA |
| siG161020040426 | si-hsa_circ_0011385_2 | AGTATAGTGCCAAGGAAAG |
| stB0005508B | genOFFTM st-h-AP2A1_002 | GGTACCGTGTGCTACAGAT |
