# Supplementary material for: circEIF3I facilitates the recruitment of SMAD3 to early endosomes to promote TGF-β signalling pathway-mediated activation of MMPs in pancreatic cancer
Source: Mol Cancer. 2023 Sep 9;22:152. doi: 10.1186/s12943-023-01847-2 (PMC10492306; doi:10.1186/s12943-023-01847-2)
Supplement: Supplementary file 11 — Additional file 11: Supplementary Table S3. MS2-TRAP assay mass spectrometry results. [file 12943_2023_1847_MOESM11_ESM.docx]

**Table S3. MS2-TRAP assay mass spectrometry results.**

| **prot_acc** | **prot_desc** | **prot_score** | **prot_mass** | **prot_matches** | **prot_matches_sig** | **prot_sequences** | **prot_sequences_sig** | **prot_cover** | **prot_pi** | **emPAI** |
| --- | --- | --- | --- | --- | --- | --- | --- | --- | --- | --- |
| A0A024R5Z7 | **ANXA2** | 1177 | 38808 | 31 | 31 | 15 | 15 | 43.1 | 7.57 | 3.73 |
| P35908 | **KRT2** | 1078 | 65678 | 30 | 30 | 21 | 21 | 37.2 | 8.07 | 2.23 |
| P13645 | **KRT10** | 890 | 59020 | 33 | 33 | 17 | 17 | 23.5 | 5.13 | 2.12 |
| A0A024R4G1 | **LRRC47** | 827 | 64004 | 27 | 27 | 13 | 13 | 21.6 | 8.55 | 1.34 |
| Q53XC0 | **EIF2S1** | 804 | 36374 | 21 | 21 | 14 | 14 | 42.5 | 5.02 | 2.69 |
| V9HWC0 | **HEL70** | 755 | 67892 | 28 | 28 | 19 | 19 | 27.9 | 6.08 | 2.11 |
| H6VRF8 | **KRT1** | 713 | 66184 | 20 | 20 | 13 | 13 | 19.9 | 8.15 | 0.97 |
| A0A0J9YVP6 | **PUF60** | 674 | 57533 | 22 | 22 | 13 | 13 | 27.9 | 5.25 | 1.44 |
| A0A024R3T8 | **PARP1** | 663 | 113811 | 22 | 22 | 16 | 16 | 20.4 | 8.99 | 0.67 |
| I3L4C2 | **BAIAP2** | 632 | 61571 | 24 | 24 | 17 | 17 | 29.7 | 9.01 | 1.55 |
| A8MX94 | **GSTP1** | 563 | 19696 | 14 | 14 | 6 | 6 | 37.9 | 5.67 | 2.02 |
| Q9UQB8 | **BAIAP2** | 562 | 61115 | 22 | 22 | 16 | 16 | 29.5 | 8.99 | 1.44 |
| P02533 | **KRT14** | 525 | 51872 | 17 | 17 | 10 | 10 | 22.5 | 5.09 | 1.23 |
| Q6IPS9 | **EEF1A1** | 516 | 50451 | 20 | 20 | 11 | 11 | 22.9 | 9.1 | 1.58 |
| P08779 | **KRT16** | 490 | 51578 | 16 | 16 | 8 | 8 | 17.3 | 4.99 | 1.1 |
| P41091 | **EIF2S3** | 474 | 51647 | 17 | 17 | 11 | 11 | 25 | 8.66 | 1.53 |
| Q9UHR4 | **BAIAP2L1** | 470 | 57189 | 12 | 12 | 10 | 10 | 24.9 | 8.82 | 0.75 |
| Q6FGJ9 | **GSTM3** | 460 | 26998 | 13 | 13 | 6 | 6 | 21.3 | 5.37 | 1.26 |
| V9HWB4 | **HEL-S-89n** | 437 | 72402 | 15 | 15 | 14 | 14 | 26.6 | 5.07 | 0.86 |
| V9HWC6 | **HEL-S-39** | 414 | 22785 | 19 | 19 | 12 | 12 | 43.3 | 9.33 | 4.2 |
| A0A024RD80 | **HSP90AB1** | 409 | 83554 | 10 | 10 | 7 | 7 | 11.5 | 4.97 | 0.36 |
| A0A1W2PPZ5 | **TCEA1** | 403 | 34370 | 11 | 11 | 7 | 7 | 21.9 | 8.64 | 1.09 |
| O14776 | **TCERG1** | 381 | 124051 | 15 | 15 | 11 | 11 | 8.8 | 8.71 | 0.4 |
| J9R021 | **eIF3a** | 376 | 166780 | 15 | 15 | 12 | 12 | 9.5 | 6.38 | 0.34 |
| P35527 | **KRT9** | 353 | 62255 | 14 | 14 | 9 | 9 | 17.5 | 5.14 | 0.67 |
| A0A075B6Z2 | **TRAJ56** | 333 | 2220 | 44 | 44 | 1 | 1 | 38.1 | 10.29 | 1.81 |
| C9JZR2 | **CTNND1** | 303 | 105354 | 9 | 9 | 7 | 7 | 8.7 | 6.41 | 0.28 |
| Q96BS4 | **FBL** | 296 | 28546 | 10 | 10 | 6 | 6 | 25.8 | 9.45 | 1.7 |
| P49756 | **RBM25** | 296 | 100467 | 8 | 8 | 5 | 5 | 7.5 | 6.06 | 0.29 |
| Q9Y295 | **DRG1** | 294 | 40802 | 8 | 8 | 6 | 6 | 18.3 | 9 | 0.6 |
| Q8TBX8 | **PIP4K2C** | 293 | 47441 | 9 | 9 | 6 | 6 | 15.2 | 6.36 | 0.5 |
| A0A024R1D9 | **NF2** | 286 | 59287 | 11 | 11 | 8 | 8 | 17.8 | 6.75 | 0.72 |
| Q01970 | **PLCB3** | 284 | 139511 | 7 | 7 | 6 | 6 | 5 | 5.68 | 0.18 |
| A0A1U9X609 | **ABCF1** | 262 | 96323 | 7 | 7 | 6 | 6 | 8.6 | 6.4 | 0.22 |
| Q9Y230 | **RUVBL2** | 254 | 51296 | 7 | 7 | 6 | 6 | 12.7 | 5.49 | 0.55 |
| P04259 | **KRT6B** | 252 | 60315 | 10 | 10 | 6 | 6 | 10.6 | 8.09 | 0.45 |
| A0A140VK11 | **CLPB** | 244 | 79193 | 9 | 9 | 6 | 6 | 10.3 | 9.13 | 0.33 |
| Q15642 | **TRIP10** | 232 | 68538 | 7 | 7 | 6 | 6 | 15.8 | 5.55 | 0.39 |
| A0A0C4DG89 | **DDX46** | 231 | 117902 | 4 | 4 | 3 | 3 | 4.2 | 9.33 | 0.09 |
| Q2VPJ6 | **HSP90AA1** | 230 | 68614 | 5 | 5 | 3 | 3 | 6 | 5.11 | 0.15 |
| Q8TEA8 | **DTD1** | 202 | 23580 | 3 | 3 | 2 | 2 | 14.8 | 8.35 | 0.3 |
| A0A2R8Y602 | **CLPB** | 201 | 43552 | 8 | 8 | 5 | 5 | 15.2 | 6.49 | 0.55 |
| A0A2R8Y793 | **ACTB** | 200 | 34405 | 7 | 7 | 6 | 6 | 28.5 | 5.02 | 0.9 |
| G3V1V0 | **MYL6** | 196 | 18311 | 7 | 7 | 4 | 4 | 26.7 | 4.68 | 0.97 |
| A0A2R8Y5S7 | **RDX** | 190 | 69407 | 7 | 7 | 6 | 6 | 10.5 | 5.96 | 0.32 |
| A0A087X1X7 | **EEF1D** | 180 | 69696 | 6 | 6 | 3 | 3 | 5.9 | 6.76 | 0.2 |
| P62306 | **SNRPF** | 180 | 9776 | 4 | 4 | 2 | 2 | 15.1 | 4.7 | 0.84 |
| B5BU01 | **EIF2S2** | 176 | 38648 | 5 | 5 | 5 | 5 | 18 | 5.72 | 0.51 |
| A8KA82 | **DNAJC3** | 167 | 58000 | 5 | 5 | 5 | 5 | 10.9 | 5.83 | 0.32 |
| Q96A33 | **CCDC47** | 166 | 56123 | 5 | 5 | 4 | 4 | 9.5 | 4.76 | 0.33 |
| Q5TZZ9 | **ANXA1** | 164 | 38918 | 5 | 5 | 5 | 5 | 16.2 | 6.57 | 0.5 |
| H0YDX7 | **HBS1L** | 160 | 61030 | 4 | 4 | 4 | 4 | 11.7 | 8.26 | 0.23 |
| A0A024RC65 | **hCG_1991735** | 157 | 189761 | 5 | 5 | 4 | 4 | 2.8 | 6.08 | 0.07 |
| Q9NVM6 | **DNAJC17** | 157 | 34780 | 5 | 5 | 5 | 5 | 16.8 | 8.61 | 0.58 |
| Q9GZL7 | **WDR12** | 157 | 48191 | 2 | 2 | 2 | 2 | 9 | 5.57 | 0.14 |
| Q9BRX9 | **WDR83** | 154 | 35119 | 6 | 6 | 3 | 3 | 8.9 | 5.36 | 0.57 |
| P13647 | **KRT5** | 153 | 62568 | 6 | 6 | 4 | 4 | 6.8 | 7.59 | 0.29 |
| A0A024RAS3 | **hCG_27698** | 153 | 50900 | 4 | 4 | 3 | 3 | 9.2 | 9.18 | 0.28 |
| F8WJN3 | **CPSF6** | 152 | 52409 | 4 | 4 | 3 | 3 | 7.7 | 6 | 0.2 |
| Q14554 | **PDIA5** | 150 | 60297 | 9 | 9 | 7 | 7 | 13.5 | 8.08 | 0.53 |
| Q9H2W6 | **MRPL46** | 148 | 31799 | 7 | 7 | 4 | 4 | 12.9 | 6.56 | 0.64 |
| A0A0S2Z4V5 | **PQBP1** | 146 | 30511 | 3 | 3 | 2 | 2 | 11.3 | 5.92 | 0.36 |
| A0A024R821 | **EIF3S9** | 142 | 92823 | 4 | 4 | 3 | 3 | 4.2 | 4.89 | 0.11 |
| A0A0D9SGE8 | **PHF6** | 140 | 42475 | 4 | 4 | 3 | 3 | 7.4 | 8.97 | 0.35 |
| F5H702 | **MRPL48** | 140 | 12819 | 4 | 4 | 3 | 3 | 31.9 | 5.18 | 1.59 |
| A0A0S2Z4R1 | **YARS** | 138 | 59448 | 7 | 7 | 6 | 6 | 10.2 | 6.61 | 0.38 |
| A0A024RDU9 | **GTF2F2** | 137 | 28420 | 8 | 8 | 4 | 4 | 12.4 | 9.24 | 0.74 |
| A0A1W2PQ51 | **DDX17** | 136 | 81091 | 4 | 4 | 4 | 4 | 6.4 | 8.63 | 0.17 |
| A0A024QZE9 | **FLJ20643** | 136 | 32582 | 4 | 4 | 4 | 4 | 16.2 | 5 | 0.47 |
| Q53SS8 | **PCBP1** | 134 | 37987 | 4 | 4 | 3 | 3 | 11.2 | 6.66 | 0.29 |
| Q08J23 | **NSUN2** | 132 | 87214 | 4 | 4 | 3 | 3 | 3.9 | 6.33 | 0.16 |
| A0A087WTA5 | **EIF2B4** | 130 | 57973 | 3 | 3 | 2 | 2 | 5 | 9.45 | 0.12 |
| H0Y8D9 | **U2SURP** | 130 | 49948 | 3 | 3 | 2 | 2 | 8.9 | 9.41 | 0.21 |
| A0A0G2JQ41 | **ABR** | 129 | 89372 | 3 | 3 | 3 | 3 | 5.3 | 7.12 | 0.11 |
| A0A024RAY2 | **KRT18** | 117 | 48029 | 4 | 4 | 2 | 2 | 4.7 | 5.34 | 0.14 |
| F8WAE5 | **EIF2A** | 116 | 64979 | 3 | 3 | 2 | 2 | 4.8 | 8.96 | 0.16 |
| Q6FHX6 | **FEN1** | 115 | 42908 | 5 | 5 | 2 | 2 | 5.5 | 8.8 | 0.25 |
| H7BZJ3 | **PDIA3** | 114 | 13739 | 4 | 4 | 4 | 4 | 32.5 | 6.78 | 1.43 |
| V9HW26 | **HEL-S-123m** | 113 | 59828 | 3 | 3 | 3 | 3 | 6.9 | 9.16 | 0.17 |
| B0QY89 | **EIF3L** | 113 | 71085 | 1 | 1 | 1 | 1 | 2.6 | 6.21 | 0.05 |
| B3KRK2 | **DDX41** | 112 | 56058 | 5 | 5 | 5 | 5 | 11.3 | 8.45 | 0.33 |
| A0A024RBR3 | **DENR** | 111 | 22477 | 1 | 1 | 1 | 1 | 9.1 | 5.21 | 0.15 |
| P40222 | **TXLNA** | 110 | 62195 | 3 | 3 | 3 | 3 | 12.6 | 6.15 | 0.17 |
| A0A024R4F1 | **ENO1** | 108 | 47481 | 2 | 2 | 2 | 2 | 6.9 | 7.01 | 0.14 |
| A0A0S2Z491 | **NPM1** | 108 | 32726 | 1 | 1 | 1 | 1 | 4.4 | 4.64 | 0.1 |
| A0A024R904 | **CACYBP** | 106 | 26308 | 2 | 2 | 1 | 1 | 4.8 | 8.28 | 0.27 |
| B8Y0L3 | **ASPH** | 105 | 48041 | 3 | 3 | 3 | 3 | 7.9 | 4.6 | 0.22 |
| Q9BRP8 | **PYM1** | 104 | 22642 | 1 | 1 | 1 | 1 | 8.8 | 9.45 | 0.15 |
| A0A0D9SF53 | **DDX3X** | 103 | 82110 | 4 | 4 | 4 | 4 | 5.3 | 8.29 | 0.17 |
| H0YKR8 | **DCAF11** | 103 | 15720 | 1 | 1 | 1 | 1 | 12.7 | 4.93 | 0.22 |
| A0A2R8Y5A0 | **CSNK2A1** | 102 | 40806 | 4 | 4 | 3 | 3 | 9 | 7.79 | 0.37 |
| A0A024R1N1 | **MYH9** | 101 | 227646 | 4 | 4 | 4 | 4 | 2.4 | 5.5 | 0.06 |
| J3QLI9 | **SNRPD1** | 98 | 8388 | 3 | 3 | 2 | 2 | 17.3 | 11.83 | 1.84 |
| A0A1W2PP34 | **HNRNPU** | 95 | 61812 | 1 | 1 | 1 | 1 | 2.7 | 9.3 | 0.05 |
| Q8IUF8 | **RIOX2** | 92 | 53109 | 2 | 2 | 2 | 2 | 5.6 | 6.23 | 0.13 |
| A0A087X0X3 | **HNRNPM** | 91 | 77746 | 5 | 5 | 5 | 5 | 6 | 8.89 | 0.18 |
| F8VUG2 | **KRT8** | 91 | 30888 | 4 | 4 | 3 | 3 | 12.1 | 5.19 | 0.51 |
| M0QXU7 | **TIMM44** | 91 | 31255 | 2 | 2 | 2 | 2 | 8.8 | 9.5 | 0.22 |
| A0A0S2Z4Z9 | **NONO** | 90 | 54311 | 3 | 3 | 3 | 3 | 7 | 9.01 | 0.19 |
| A0A024QZ70 | **TAOK1** | 88 | 116454 | 5 | 5 | 4 | 4 | 4.4 | 7.3 | 0.15 |
| J3KTA4 | **DDX5** | 86 | 69557 | 3 | 3 | 3 | 3 | 5.5 | 9.01 | 0.15 |
| A0A024R681 | **NUMB** | 86 | 66485 | 3 | 3 | 3 | 3 | 5.8 | 8.91 | 0.16 |
| A0A024R8R0 | **SAP30BP** | 85 | 33964 | 2 | 2 | 2 | 2 | 9.1 | 4.74 | 0.2 |
| Q6PJ77 | **BTF3L4** | 84 | 16461 | 3 | 3 | 2 | 2 | 24.8 | 5.41 | 0.45 |
| A0MNN4 | **SMU1** | 84 | 58134 | 1 | 1 | 1 | 1 | 3.5 | 6.74 | 0.06 |
| Q6P1N2 | **MTIF2** | 83 | 52002 | 1 | 1 | 1 | 1 | 3.7 | 7.12 | 0.06 |
| A0A024R3W7 | **EEF1B2** | 82 | 24919 | 1 | 1 | 1 | 1 | 6.7 | 4.5 | 0.13 |
| H0YDT6 | **EIF3F** | 82 | 10801 | 1 | 1 | 1 | 1 | 17 | 5.48 | 0.32 |
| B7ZLC9 | **GEMIN5** | 81 | 170664 | 1 | 1 | 1 | 1 | 0.9 | 6.17 | 0.02 |
| Q4KMP7 | **TBC1D10B** | 78 | 87658 | 3 | 3 | 2 | 2 | 5.1 | 9.27 | 0.12 |
| A0A024R5Z3 | **SMAD3** | 78 | 48905 | 2 | 2 | 1 | 1 | 3.1 | 6.73 | 0.07 |
| V9HWE1 | **HEL113** | 77 | 53676 | 2 | 2 | 2 | 2 | 5.6 | 5.06 | 0.13 |
| A0A087X0W9 | **OTUD6B** | 77 | 37588 | 1 | 1 | 1 | 1 | 4 | 6.31 | 0.09 |
| A0A024R6Q1 | **EIF5** | 76 | 49648 | 2 | 2 | 2 | 2 | 4.2 | 5.41 | 0.14 |
| P31689 | **DNAJA1** | 71 | 45581 | 3 | 3 | 3 | 3 | 8.3 | 6.65 | 0.23 |
| M0QY97 | **ZC3H4** | 71 | 96048 | 1 | 1 | 1 | 1 | 2.1 | 6.81 | 0.03 |
| P0DN76 | **U2AF1L5** | 70 | 28368 | 1 | 1 | 1 | 1 | 5.4 | 9.09 | 0.12 |
| A0A024R029 | **ABT1** | 69 | 31117 | 3 | 3 | 3 | 3 | 11.8 | 9.87 | 0.36 |
| A0A0S2Z5F3 | **TBL2** | 69 | 50265 | 2 | 2 | 2 | 2 | 5.6 | 9.48 | 0.14 |
| A0A384MDW7 | **ECHS1** | 69 | 31835 | 1 | 1 | 1 | 1 | 4.1 | 8.34 | 0.1 |
| B4DVS0 | **SNRPB** | 67 | 24300 | 2 | 2 | 2 | 2 | 9.4 | 10.76 | 0.29 |
| Q9NQ50 | **MRPL40** | 65 | 24475 | 3 | 3 | 2 | 2 | 7.3 | 9.62 | 0.47 |
| Q9Y5B9 | **SUPT16H** | 65 | 120409 | 2 | 2 | 2 | 2 | 2.3 | 5.5 | 0.06 |
| Q5U0F4 | **EIF3S2** | 65 | 36878 | 2 | 2 | 2 | 2 | 6.5 | 5.38 | 0.19 |
| O95782 | **AP2A1** | 64 | 108561 | 2 | 2 | 2 | 2 | 2.1 | 6.63 | 0.06 |
| A0A087WZK9 | **EIF3H** | 64 | 39736 | 1 | 1 | 1 | 1 | 5.7 | 5.96 | 0.08 |
| Q9NV56 | **MRGBP** | 64 | 22574 | 1 | 1 | 1 | 1 | 6.4 | 5.57 | 0.15 |
| S4R347 | **FNBP1L** | 63 | 70938 | 2 | 2 | 2 | 2 | 4.3 | 6.2 | 0.09 |
| E9PB61 | **ALYREF** | 63 | 27541 | 2 | 2 | 2 | 2 | 11 | 11.05 | 0.26 |
| P41227 | **NAA10** | 63 | 26613 | 2 | 2 | 2 | 2 | 10.2 | 5.41 | 0.27 |
| H0Y6J6 | **RBM17** | 63 | 25268 | 1 | 1 | 1 | 1 | 7.1 | 8.64 | 0.13 |
| B1AHB0 | **MCM5** | 62 | 83031 | 3 | 3 | 2 | 2 | 3.1 | 8.64 | 0.12 |
| P20290 | **BTF3** | 62 | 22211 | 3 | 3 | 3 | 3 | 22.3 | 9.41 | 0.52 |
| A0A0U1RQH7 | **RBM39** | 62 | 26853 | 2 | 2 | 2 | 2 | 11.4 | 7.68 | 0.26 |
| K7EJT5 | **RPL22** | 62 | 5080 | 1 | 1 | 1 | 1 | 27.7 | 9.39 | 0.72 |
| D3DVA5 | **ARHGEF2** | 61 | 116954 | 4 | 4 | 4 | 4 | 3.3 | 7.08 | 0.12 |
| A0A024R8U5 | **SFRS2** | 61 | 25461 | 1 | 1 | 1 | 1 | 7.7 | 11.86 | 0.13 |
| A0A024R6K1 | **CCNK** | 60 | 64598 | 1 | 1 | 1 | 1 | 2.8 | 8.6 | 0.05 |
| P57772 | **EEFSEC** | 59 | 65890 | 1 | 1 | 1 | 1 | 1.7 | 8.61 | 0.05 |
| Q1M183 | **HMGA2** | 59 | 16485 | 1 | 1 | 1 | 1 | 15.6 | 11.51 | 0.2 |
| Q5THR1 | **DHX35** | 58 | 76626 | 4 | 4 | 4 | 4 | 4.4 | 8.89 | 0.18 |
| F5H282 | **TCP1** | 58 | 36718 | 4 | 4 | 3 | 3 | 9 | 6.56 | 0.3 |
| A0A024R4X1 | **PACSIN2** | 58 | 50275 | 1 | 1 | 1 | 1 | 2.1 | 6.04 | 0.07 |
| A0A0B4J1W3 | **NAA15** | 57 | 102391 | 2 | 2 | 2 | 2 | 2.9 | 7.23 | 0.06 |
| A0A024R6S1 | **DNAJA2** | 57 | 46344 | 2 | 2 | 2 | 2 | 4.4 | 6.06 | 0.15 |
| A0A2R8Y3X5 | **OPA1** | 57 | 99527 | 1 | 1 | 1 | 1 | 1.8 | 5.78 | 0.03 |
| J3KPP4 | **LUC7L3** | 56 | 58698 | 1 | 1 | 1 | 1 | 2.2 | 9.93 | 0.06 |
| A0A075B724 | **TUBB8** | 56 | 13104 | 1 | 1 | 1 | 1 | 12.6 | 5.22 | 0.26 |
| Q1W6G4 | **LUC7L** | 55 | 38781 | 2 | 2 | 2 | 2 | 6.5 | 10.06 | 0.18 |
| A0A024R983 | **TROVE2** | 54 | 61372 | 1 | 1 | 1 | 1 | 2.8 | 8.27 | 0.05 |
| Q0VDF9 | **HSPA14** | 54 | 55444 | 1 | 1 | 1 | 1 | 1.6 | 5.41 | 0.06 |
| Q0VAS5 | **HIST1H4H** | 53 | 11364 | 2 | 2 | 2 | 2 | 19.4 | 11.04 | 0.7 |
| Q14320 | **FAM50A** | 52 | 40216 | 2 | 2 | 1 | 1 | 4.7 | 6.39 | 0.17 |
| H7C4H2 | **SRPRB** | 52 | 17716 | 1 | 1 | 1 | 1 | 11.9 | 7.27 | 0.19 |
| A0A0G2JMZ6 | **KIF5C** | 51 | 99019 | 1 | 1 | 1 | 1 | 1.5 | 5.82 | 0.03 |
| A0A090N8Y2 | **ERP70** | 51 | 73229 | 1 | 1 | 1 | 1 | 1.9 | 4.96 | 0.04 |
| S4R3J5 | **DNAJC8** | 51 | 13083 | 1 | 1 | 1 | 1 | 10.4 | 10.21 | 0.26 |
| Q8ND56 | **LSM14A** | 50 | 50727 | 3 | 3 | 2 | 2 | 3.9 | 9.55 | 0.13 |
| A0A161SXE1 | **EIF3E** | 50 | 20449 | 1 | 1 | 1 | 1 | 7 | 5.01 | 0.16 |
| H7C333 | **NIPSNAP2** | 50 | 13826 | 1 | 1 | 1 | 1 | 7.9 | 5.93 | 0.25 |
| A0A087X2G1 | **DDX1** | 49 | 74898 | 2 | 2 | 1 | 1 | 1.7 | 7.81 | 0.04 |
| A0A0C4DGG1 | **PACSIN3** | 49 | 46751 | 1 | 1 | 1 | 1 | 2.7 | 5.82 | 0.07 |
| Q5SY38 | **EIF2D** | 49 | 29481 | 1 | 1 | 1 | 1 | 4.4 | 5.54 | 0.11 |
| A0A024R017 | **HIST1H2AC** | 48 | 14097 | 4 | 4 | 3 | 3 | 26.9 | 11.05 | 0.91 |
| P48426 | **PIP4K2A** | 48 | 46424 | 1 | 1 | 1 | 1 | 2.7 | 6.5 | 0.07 |
| A0A087WWY6 | **ILK** | 48 | 12617 | 1 | 1 | 1 | 1 | 9 | 5.73 | 0.27 |
| B0QYA4 | **EIF3D** | 47 | 12047 | 1 | 1 | 1 | 1 | 13.3 | 9.5 | 0.28 |
| H7C5M9 | **PDCD10** | 46 | 14220 | 2 | 2 | 1 | 1 | 8.2 | 5.54 | 0.24 |
| B0YJ73 | **RSU1** | 46 | 25830 | 1 | 1 | 1 | 1 | 2.6 | 6.71 | 0.13 |
| A0A0A0MRQ5 | **PRDX1** | 46 | 10727 | 1 | 1 | 1 | 1 | 11.3 | 8.79 | 0.32 |
| A0A0A0MRP6 | **SMARCA1** | 45 | 123471 | 1 | 1 | 1 | 1 | 0.9 | 8.64 | 0.03 |
| M0R080 | **DNAJB1** | 45 | 20655 | 1 | 1 | 1 | 1 | 5.5 | 8.74 | 0.16 |
| P62318 | **SNRPD3** | 45 | 14021 | 1 | 1 | 1 | 1 | 7.9 | 10.33 | 0.24 |
| F5H013 | **SNRPG** | 45 | 7705 | 1 | 1 | 1 | 1 | 17.6 | 6.54 | 0.46 |
| Q8IVM0 | **CCDC50** | 44 | 35914 | 1 | 1 | 1 | 1 | 3.9 | 6.2 | 0.09 |
| A0A3Q8AIR8 | **PRMT1** | 43 | 32305 | 1 | 1 | 1 | 1 | 3.9 | 4.96 | 0.1 |
| A0A2R8Y611 | **AP3D1** | 42 | 107041 | 1 | 1 | 1 | 1 | 1.4 | 6.35 | 0.03 |
| A0A087X020 | **SBDS** | 42 | 29131 | 1 | 1 | 1 | 1 | 3.2 | 8.91 | 0.11 |
| A0A024RDL1 | **CCT6A** | 41 | 58444 | 2 | 2 | 2 | 2 | 3 | 6.23 | 0.12 |
| A0A024R2L1 | **WDR48** | 41 | 77018 | 1 | 1 | 1 | 1 | 1.5 | 6.59 | 0.04 |
| Q6IBR0 | **RPN1** | 41 | 68678 | 1 | 1 | 1 | 1 | 2.5 | 5.96 | 0.05 |
| E5KSU5 | **TFAM** | 40 | 29306 | 1 | 1 | 1 | 1 | 4.1 | 9.74 | 0.11 |
| A0A024R6W2 | **NUDT21** | 40 | 26268 | 1 | 1 | 1 | 1 | 3.5 | 8.85 | 0.13 |
| A0A0B4J2C3 | **TPT1** | 40 | 22787 | 1 | 1 | 1 | 1 | 7.1 | 5.11 | 0.15 |
| D3DWL9 | **CPSF1** | 39 | 153087 | 1 | 1 | 1 | 1 | 1.2 | 5.92 | 0.02 |
| X6RAJ1 | **AKAP17A** | 39 | 51987 | 1 | 1 | 1 | 1 | 1.6 | 9.45 | 0.06 |
| E5KTM5 | **TFB1M** | 39 | 39860 | 1 | 1 | 1 | 1 | 2.3 | 9.35 | 0.08 |
| B2R491 | **RPS4X** | 39 | 29807 | 1 | 1 | 1 | 1 | 2.7 | 10.16 | 0.11 |
| E5RH41 | **GTF2E2** | 39 | 22126 | 1 | 1 | 1 | 1 | 4 | 9.65 | 0.15 |
| A0A024R0J9 | **HNRPUL1** | 38 | 85311 | 1 | 1 | 1 | 1 | 1.3 | 8.94 | 0.04 |
| B0YIW5 | **ARCN1** | 38 | 57630 | 1 | 1 | 1 | 1 | 2.3 | 5.89 | 0.06 |
| A0A024R0H7 | **WDR77** | 38 | 37442 | 1 | 1 | 1 | 1 | 2 | 5.03 | 0.09 |
| Q5RLJ0 | **CLE** | 38 | 28066 | 1 | 1 | 1 | 1 | 3.3 | 6 | 0.12 |
| H7C1K0 | **SRRT** | 38 | 5606 | 1 | 1 | 1 | 1 | 23.4 | 10.81 | 0.64 |
| A0A024R4E5 | **HDLBP** | 37 | 141979 | 1 | 1 | 1 | 1 | 0.6 | 6.43 | 0.02 |
| A0A024R5Z9 | **PKM2** | 37 | 58538 | 1 | 1 | 1 | 1 | 1.3 | 7.6 | 0.06 |
| Q6IAM0 | **EIF3G** | 37 | 35864 | 1 | 1 | 1 | 1 | 2.8 | 5.87 | 0.09 |
| A0A0S2Z508 | **ARHGAP26** | 36 | 78527 | 1 | 1 | 1 | 1 | 1.5 | 6.81 | 0.04 |
| A0A024QZR5 | **AP3M1** | 36 | 47251 | 1 | 1 | 1 | 1 | 2.4 | 6.47 | 0.07 |
| J3QRS3 | **MYL12A** | 36 | 20501 | 1 | 1 | 1 | 1 | 5.6 | 4.62 | 0.16 |
| A0A0A0MSK5 | **TOR1AIP1** | 35 | 52545 | 1 | 1 | 1 | 1 | 3 | 6.59 | 0.06 |
| H0YJC1 | **FRMD6** | 35 | 23094 | 1 | 1 | 1 | 1 | 10.2 | 8.73 | 0.15 |
| A4FU77 | **SNRNP200** | 34 | 217585 | 1 | 1 | 1 | 1 | 0.5 | 6.05 | 0.01 |
| Q8TDB6 | **DTX3L** | 34 | 84585 | 1 | 1 | 1 | 1 | 0.8 | 8.31 | 0.04 |
| A0A024R6K3 | **SETD3** | 34 | 67557 | 1 | 1 | 1 | 1 | 2.5 | 5.72 | 0.05 |
| Q3B726 | **TWISTNB** | 34 | 37922 | 1 | 1 | 1 | 1 | 3.8 | 6.53 | 0.09 |
| H0Y580 | **EIF2B3** | 34 | 25394 | 1 | 1 | 1 | 1 | 4.1 | 8.36 | 0.13 |
| H0YA84 | **BRD8** | 33 | 21173 | 1 | 1 | 1 | 1 | 4.1 | 4.11 | 0.16 |
| Q5VT82 | **PCDH9** | 32 | 114097 | 1 | 1 | 1 | 1 | 1.7 | 5.23 | 0.03 |
| A0A024RBB7 | **NAP1L1** | 32 | 45631 | 1 | 1 | 1 | 1 | 2.6 | 4.36 | 0.07 |
| B4DVD7 | **EIF3K** | 32 | 24771 | 1 | 1 | 1 | 1 | 5.2 | 4.76 | 0.14 |
| H7C4Y9 | **RAB3GAP2** | 32 | 19490 | 1 | 1 | 1 | 1 | 5.2 | 5.39 | 0.17 |
| G3V1A4 | **CFL1** | 32 | 17029 | 1 | 1 | 1 | 1 | 7.4 | 8.54 | 0.2 |
| A0A0A0MT16 | **ABCA13** | 31 | 580525 | 1 | 1 | 1 | 1 | 0.2 | 5.99 | 0.01 |
| A0A0A0MTT8 | **GDF15** | 31 | 12467 | 1 | 1 | 1 | 1 | 6.2 | 6.92 | 0.28 |
| L0R5A1 | **CSF2RB** | 31 | 11752 | 1 | 1 | 1 | 1 | 7.4 | 11.3 | 0.29 |
| H0YG37 | **REXO2** | 30 | 9838 | 1 | 1 | 1 | 1 | 7.8 | 4.78 | 0.35 |
| O94804 | **STK10** | 29 | 112749 | 1 | 1 | 1 | 1 | 1.1 | 6.52 | 0.03 |
| A0A024RBI5 | **CORO1C** | 29 | 53899 | 1 | 1 | 1 | 1 | 2.3 | 6.65 | 0.06 |
| H3BTE3 | **AMDHD2** | 29 | 28975 | 1 | 1 | 1 | 1 | 2.2 | 6.15 | 0.11 |
| H0YJ77 | **PRMT5** | 29 | 16259 | 1 | 1 | 1 | 1 | 6.5 | 4.98 | 0.21 |
| H0YLI4 | **MESD** | 25 | 8866 | 1 | 1 | 1 | 1 | 11.3 | 6.54 | 0.39 |
| Q96MT7 | **CFAP44** | 24 | 214985 | 1 | 1 | 1 | 1 | 0.6 | 5.28 | 0.02 |
| A0A024QZZ7 | **HIST1H2BD** | 24 | 13928 | 1 | 1 | 1 | 1 | 7.1 | 10.31 | 0.25 |
