# Supplementary material for: circEIF3I facilitates the recruitment of SMAD3 to early endosomes to promote TGF-β signalling pathway-mediated activation of MMPs in pancreatic cancer
Source: Mol Cancer. 2023 Sep 9;22:152. doi: 10.1186/s12943-023-01847-2 (PMC10492306; doi:10.1186/s12943-023-01847-2)
Supplement: Supplementary file 12 — Additional file 12: Supplementary Table S4. The interacting atoms of the two nodes involved (residues/ligands) with relative distances less than 3.5Å. [file 12943_2023_1847_MOESM12_ESM.docx]

**Table S4. The interacting atoms of the two nodes involved (residues/ligands) with relative distances less than 3.5Å.**

**circEIF3I-SMAD3**

| **circEIF3I** | **Interaction** | **SMAD3** | **Distance** | **Atom1** | **Atom2** |
| --- | --- | --- | --- | --- | --- |
| A:14:_:C | IAC:LIG_SC | A:92:_:TRP | **0.867** | O2' | HH2 |
| A:252:_:G | IAC:LIG_SC | A:325:_:GLY | **1.273** | O2' | HA1 |
| A:15:_:C | IAC:LIG_SC | A:93:_:ARG | **1.95** | C1' | HH21 |
| A:16:_:U | IAC:LIG_MC | A:310:_:ASP | **1.955** | O2' | O |
| A:17:_:U | IAC:LIG_SC | A:311:_:SER | **1.961** | O4' | HG |
| A:15:_:C | IAC:LIG_SC | A:92:_:TRP | **1.988** | C5' | HZ2 |
| A:195:_:A | IAC:LIG_SC | A:357:_:ASN | **2.054** | C5' | HD22 |
| A:253:_:G | IAC:LIG_MC | A:324:_:TYR | **2.092** | C5' | O |
| A:264:_:C | IAC:SC_LIG | A:23:_:GLN | **2.223** | C4' | HG3 |
| A:13:_:C | IAC:LIG_MC | A:17:_:GLY | **2.354** | O2' | O |
| A:19:_:U | IAC:LIG_SC | A:383:_:GLU | **2.47** | O2' | HB3 |
| A:18:_:C | IAC:LIG_SC | A:384:_:TYR | **2.488** | O2' | HE1 |
| A:13:_:C | IAC:LIG_SC | A:20:_:LYS | **2.521** | O4' | HD2 |
| A:158:_:U | IAC:LIG_MC | A:199:_:SER | **2.528** | O2' | O |
| A:16:_:U | IAC:LIG_SC | A:311:_:SER | **2.66** | O2' | HG |
| A:252:_:G | IAC:LIG_MC | A:323:_:ARG | **2.783** | O3' | O |
| A:14:_:C | IAC:LIG_SC | A:18:_:TRP | **2.827** | C5' | HA |
| A:15:_:C | IAC:LIG_MC | A:193:_:ASP | **2.952** | N4 | O |
| A:252:_:G | IAC:LIG_MC | A:324:_:TYR | **2.979** | O2' | O |
| A:263:_:C | IAC:SC_LIG | A:23:_:GLN | **3.035** | O2' | HE21 |
| A:17:_:U | IAC:LIG_SC | A:335:_:PRO | **3.076** | O2 | HB3 |
| A:253:_:G | IAC:LIG_MC | A:323:_:ARG | **3.135** | OP1 | O |
| A:17:_:U | IAC:LIG_MC | A:310:_:ASP | **3.165** | O4' | O |
| A:12:_:G | IAC:LIG_SC | A:20:_:LYS | **3.192** | O2' | HE3 |
| A:15:_:C | IAC:LIG_SC | A:58:_:GLN | **3.234** | C5' | OE1 |
| A:15:_:C | IAC:LIG_SC | A:194:_:HIS | **3.242** | O2 | ND1 |
| A:19:_:U | IAC:LIG_SC | A:384:_:TYR | **3.25** | O4' | HE1 |
| A:18:_:C | IAC:LIG_SC | A:314:_:PHE | **3.384** | C1' | HZ |
| A:253:_:G | IAC:LIG_SC | A:325:_:GLY | **3.393** | O4' | HA1 |
| A:16:_:U | IAC:LIG_SC | A:58:_:GLN | **3.426** | OP1 | HE22 |
| A:17:_:U | IAC:LIG_SC | A:196:_:MET | **3.437** | O4 | HB2 |
| A:158:_:U | IAC:LIG_SC | A:201:_:ASP | **3.439** | O2' | H |
| A:17:_:U | VDW:LIG_SC | A:336:_:PRO | **3.442** | C2 | CD |
| A:195:_:A | IAC:LIG_SC | A:358:_:GLN | **3.448** | OP1 | OE1 |
| A:252:_:G | IAC:LIG_MC | A:322:_:GLN | **3.485** | C4' | O |

**circEIF3I-AP2A1**

| **circEIF3I** | **Interaction** | **AP2A1** | **Distance** | **Atom1** | **Atom2** |
| --- | --- | --- | --- | --- | --- |
| A:192:_:A | IAC:LIG_SC | A:298:_:LYS | **1.376** | O3' | HZ3 |
| A:155:_:C | IAC:LIG_SC | A:411:_:ARG | **1.725** | OP2 | HH22 |
| A:37:_:U | IAC:LIG_SC | A:900:_:ASP | **1.787** | O2' | HA |
| A:256:_:A | IAC:LIG_SC | A:375:_:ASN | **1.792** | O3' | HD21 |
| A:193:_:A | IAC:LIG_SC | A:298:_:LYS | **2.036** | OP1 | HZ1 |
| A:37:_:U | IAC:LIG_SC | A:901:_:ALA | **2.094** | O4' | HB1 |
| A:257:_:G | IAC:LIG_SC | A:375:_:ASN | **2.164** | C5' | HD22 |
| A:189:_:U | IAC:LIG_SC | A:336:_:GLN | **2.239** | C4' | HG2 |
| A:191:_:U | IAC:LIG_SC | A:295:_:PRO | **2.296** | OP1 | HD3 |
| A:137:_:U | IAC:LIG_SC | A:950:_:GLN | **2.315** | O4 | HE22 |
| A:36:_:G | IAC:LIG_SC | A:923:_:PRO | **2.393** | OP1 | HG2 |
| A:154:_:A | IAC:LIG_SC | A:411:_:ARG | **2.44** | O3' | HE |
| A:36:_:G | IAC:LIG_SC | A:924:_:GLU | **2.584** | OP1 | HG2 |
| A:255:_:G | VDW:LIG_SC | A:339:_:GLN | **2.676** | C2 | OE1 |
| A:136:_:A | IAC:LIG_SC | A:922:_:ASN | **2.738** | N3 | HB2 |
| A:192:_:A | IAC:LIG_SC | A:295:_:PRO | **2.741** | OP1 | HG2 |
| A:40:_:G | IAC:LIG_SC | A:572:_:ALA | **2.873** | OP2 | HB3 |
| A:137:_:U | VDW:LIG_SC | A:950:_:GLN | **2.904** | C5 | NE2 |
| A:25:_:C | IAC:LIG_SC | A:418:_:TYR | **2.977** | OP1 | OH |
| A:190:_:C | IAC:LIG_SC | A:336:_:GLN | **2.984** | OP1 | OE1 |
| A:135:_:G | IAC:LIG_SC | A:948:_:ASN | **3.021** | OP1 | HD22 |
| A:37:_:U | IAC:LIG_MC | A:899:_:MET | **3.022** | O2' | O |
| A:87:_:G | IAC:LIG_SC | A:894:_:LYS | **3.156** | O4' | HZ1 |
| A:72:_:A | VDW:LIG_SC | A:735:_:PRO | **3.177** | C8 | CD |
| A:256:_:A | IAC:LIG_SC | A:339:_:GLN | **3.181** | O2' | HE22 |
| A:37:_:U | IAC:LIG_SC | A:924:_:GLU | **3.186** | OP1 | OE1 |
| A:39:_:C | IAC:LIG_SC | A:572:_:ALA | **3.21** | O3' | HB1 |
| A:86:_:G | IAC:LIG_SC | A:894:_:LYS | **3.222** | O2' | HZ1 |
| A:35:_:U | IAC:LIG_SC | A:923:_:PRO | **3.248** | O3' | HB2 |
| A:38:_:C | IAC:LIG_MC | A:899:_:MET | **3.324** | OP2 | O |
| A:154:_:A | IAC:LIG_SC | A:415:_:THR | **3.375** | O2' | HG21 |
| A:71:_:C | VDW:LIG_SC | A:732:_:ASP | **3.442** | C2 | CG |
| A:156:_:A | IAC:LIG_SC | A:379:_:THR | **3.49** | OP1 | HG23 |
| A:71:_:C | IAC:LIG_MC | A:731:_:GLU | **3.5** | O2' | O |
