# Supplementary material for: circEIF3I facilitates the recruitment of SMAD3 to early endosomes to promote TGF-β signalling pathway-mediated activation of MMPs in pancreatic cancer
Source: Mol Cancer. 2023 Sep 9;22:152. doi: 10.1186/s12943-023-01847-2 (PMC10492306; doi:10.1186/s12943-023-01847-2)
Supplement: Supplementary file 13 — Additional file 13: Supplementary Table S5. The identified motifs of SMAD3 and AP2A1 using RIP-seq. [file 12943_2023_1847_MOESM13_ESM.docx]

**Table S5. The identified motifs of SMAD3 and AP2A1 using RIP-seq.**

**SMAD3 motifs**

| MOTIF  INDEX | MOTIF  SOURCE | MOTIF_ID | ALT_ID | CONSENSUS | WIDTH | SITES | E-VALUE | E-VALUE SOURCE |
| --- | --- | --- | --- | --- | --- | --- | --- | --- |
| 1 | STREME | 1-AAGGUAAG | STREME-1 | AAGGUAAG | 8 | 6805 | 1.90E-37 | STREME |
| 2 | MEME | UUUNUUUU | MEME-1 | UUUNUUUU | 8 | 8015 | 1.40E-16 | MEME |
| 3 | STREME | 2-UUUSUUUU | STREME-2 | UUUSUUUU | 8 | 4754 | 2.00E-13 | STREME |
| 4 | STREME | 3-GAUGAAGA | STREME-3 | GAUGAAGA | 8 | 3651 | 7.20E-12 | STREME |
| 5 | STREME | 4-CAUCAUCA | STREME-4 | CAUCAUCA | 8 | 4163 | 1.20E-10 | STREME |
| 6 | STREME | 5-GGUAAGU | STREME-5 | GGUAAGU | 7 | 4226 | 3.50E-10 | STREME |
| 7 | STREME | 6-GAAGAAG | STREME-6 | GAAGAAG | 7 | 5795 | 8.50E-09 | STREME |
| 8 | STREME | 7-GCRGYGGC | STREME-7 | GCRGYGGC | 8 | 3080 | 1.10E-08 | STREME |
| 9 | STREME | 8-GCUGCUG | STREME-8 | GCUGCUG | 7 | 3635 | 1.20E-08 | STREME |
| 10 | STREME | 9-UAAUCCCA | STREME-9 | UAAUCCCA | 8 | 310 | 1.20E-06 | STREME |
| 11 | STREME | 10-CCGYCUCC | STREME-10 | 10-CCGYCUCC | 8 | 3026 | 1.60E-06 | STREME |
| 12 | STREME | 11-AAAUUAGC | STREME-11 | 11-AAAUUAGC | 8 | 213 | 6.50E-06 | STREME |
| 13 | STREME | 12-UAAAAAUA | STREME-12 | 12-UAAAAAUA | 8 | 1436 | 1.10E-05 | STREME |
| 14 | STREME | 13-ACUUUGGG | STREME-13 | 13-ACUUUGGG | 8 | 322 | 1.20E-05 | STREME |
| 15 | STREME | 14-AGGCCGAG | STREME-14 | 14-AGGCCGAG | 8 | 1405 | 2.30E-05 | STREME |
| 16 | STREME | 15-GGASSUGG | STREME-15 | 15-GGASSUGG | 8 | 5360 | 3.20E-05 | STREME |
| 17 | STREME | 16-AGAAGCUG | STREME-16 | 16-AGAAGCUG | 8 | 2984 | 3.30E-05 | STREME |
| 18 | STREME | 17-ACCCCAA | STREME-17 | 17-ACCCCAA | 7 | 862 | 4.40E-05 | STREME |
| 19 | STREME | 18-CAGCCUGG | STREME-18 | 18-CAGCCUGG | 8 | 728 | 1.10E-04 | STREME |
| 20 | STREME | 19-UGUUGCC | STREME-19 | 19-UGUUGCC | 7 | 865 | 2.20E-04 | STREME |
| 21 | STREME | 20-UUGUYUUU | STREME-20 | 20-UUGUYUUU | 8 | 1875 | 3.00E-04 | STREME |
| 22 | STREME | 21-UGAUGAC | STREME-21 | 21-UGAUGAC | 7 | 5589 | 3.40E-04 | STREME |
| 23 | STREME | 22-UGGGAUUA | STREME-22 | 22-UGGGAUUA | 8 | 289 | 4.80E-04 | STREME |
| 24 | STREME | 23-CUGCAG | STREME-23 | 23-CUGCAG | 6 | 4156 | 5.00E-04 | STREME |
| 25 | STREME | 24-CAACRUGG | STREME-24 | 24-CAACRUGG | 8 | 371 | 7.10E-04 | STREME |
| 26 | STREME | 25-GWUCGAG | STREME-25 | 25-GWUCGAG | 7 | 839 | 9.90E-04 | STREME |
| 27 | STREME | 26-GGGGUGGG | STREME-26 | 26-GGGGUGGG | 8 | 1210 | 1.40E-03 | STREME |
| 28 | STREME | 27-UCAGCCUC | STREME-27 | 27-UCAGCCUC | 8 | 579 | 2.00E-03 | STREME |
| 29 | STREME | 28-CAACAAC | STREME-28 | 28-CAACAAC | 7 | 2019 | 2.00E-03 | STREME |
| 30 | STREME | 29-UCGAACUC | STREME-29 | 29-UCGAACUC | 8 | 88 | 2.10E-03 | STREME |
| 31 | STREME | 30-UUUAAAA | STREME-30 | 30-UUUAAAA | 7 | 1703 | 2.40E-03 | STREME |
| 32 | STREME | 31-GCUGGAGU | STREME-31 | 31-GCUGGAGU | 8 | 332 | 3.20E-03 | STREME |
| 33 | STREME | 32-UCUCGGCU | STREME-32 | 32-UCUCGGCU | 8 | 138 | 4.00E-03 | STREME |
| 34 | STREME | 33-AAAUA | STREME-33 | 33-AAAUA | 5 | 5931 | 4.50E-03 | STREME |
| 35 | STREME | 34-UUAUUU | STREME-34 | 34-UUAUUU | 6 | 2884 | 4.60E-03 | STREME |
| 36 | STREME | 35-AGGUAA | STREME-35 | 35-AGGUAA | 6 | 374 | 4.80E-03 | STREME |
| 37 | STREME | 36-UAUUUUUA | STREME-36 | 36-UAUUUUUA | 8 | 1942 | 5.00E-03 | STREME |
| 38 | STREME | 37-UGGGGA | STREME-37 | 37-UGGGGA | 6 | 2554 | 5.90E-03 | STREME |
| 39 | STREME | 38-CUUCCUC | STREME-38 | 38-CUUCCUC | 7 | 3134 | 7.00E-03 | STREME |
| 40 | STREME | 39-GACUACAG | STREME-39 | 39-GACUACAG | 8 | 173 | 7.60E-03 | STREME |
| 41 | STREME | 40-CCCACCCC | STREME-40 | 40-CCCACCCC | 8 | 1645 | 7.60E-03 | STREME |
| 42 | STREME | 41-UCACGCCU | STREME-41 | 41-UCACGCCU | 8 | 148 | 9.20E-03 | STREME |
| 43 | STREME | 42-GUCAUUG | STREME-42 | 42-GUCAUUG | 7 | 5103 | 9.30E-03 | STREME |
| 44 | STREME | 43-CCCAGCCC | STREME-43 | 43-CCCAGCCC | 8 | 1631 | 1.00E-02 | STREME |
| 45 | STREME | 44-UAGAGACG | STREME-44 | 44-UAGAGACG | 8 | 114 | 1.10E-02 | STREME |
| 46 | STREME | 45-AAGCGAUU | STREME-45 | 45-AAGCGAUU | 8 | 91 | 1.20E-02 | STREME |
| 47 | STREME | 46-ACURCAA | STREME-46 | 46-ACURCAA | 7 | 1070 | 1.20E-02 | STREME |
| 48 | STREME | 47-GCCACCAC | STREME-47 | 47-GCCACCAC | 8 | 258 | 1.70E-02 | STREME |
| 49 | STREME | 48-AUCGCUUG | STREME-48 | 48-AUCGCUUG | 8 | 93 | 2.10E-02 | STREME |
| 50 | STREME | 49-CACCGUG | STREME-49 | 49-CACCGUG | 7 | 428 | 2.10E-02 | STREME |
| 51 | STREME | 50-CCCAGCUA | STREME-50 | 50-CCCAGCUA | 8 | 314 | 2.60E-02 | STREME |
| 52 | STREME | 51-ACUCGGG | STREME-51 | 51-ACUCGGG | 7 | 254 | 3.00E-02 | STREME |
| 53 | STREME | 52-UUGCAGU | STREME-52 | 52-UUGCAGU | 7 | 628 | 3.10E-02 | STREME |
| 54 | STREME | 53-UCUCUAC | STREME-53 | 53-UCUCUAC | 7 | 405 | 3.50E-02 | STREME |
| 55 | STREME | 54-GCCACYGC | STREME-54 | 54-GCCACYGC | 8 | 507 | 3.50E-02 | STREME |
| 56 | STREME | 55-UCUUCCU | STREME-55 | 55-UCUUCCU | 7 | 5242 | 3.60E-02 | STREME |
| 57 | STREME | 56-GCCGAGAU | STREME-56 | 56-GCCGAGAU | 8 | 105 | 3.70E-02 | STREME |
| 58 | STREME | 57-UGGAGAA | STREME-57 | 57-UGGAGAA | 7 | 833 | 4.00E-02 | STREME |

**AP2A1 motifs**

| MOTIF  INDEX | MOTIF  SOURCE | MOTIF_ID | ALT_ID | CONSENSUS | WIDTH | SITES | E-VALUE | E-VALUE  SOURCE |
| --- | --- | --- | --- | --- | --- | --- | --- | --- |
| 1 | STREME | 1-AGGUAAGU | STREME-1 | AGGUAAGU | 8 | 5403 | 1.60E-23 | STREME |
| 2 | STREME | 2-UUUVUUUU | STREME-2 | UUUVUUUU | 8 | 5860 | 6.50E-14 | STREME |
| 3 | STREME | 3-GAUGAUGA | STREME-3 | GAUGAUGA | 8 | 4599 | 1.70E-09 | STREME |
| 4 | STREME | 4-AGDAGCUG | STREME-4 | AGDAGCUG | 8 | 2476 | 4.40E-07 | STREME |
| 5 | MEME | UUUKWUUU | MEME-1 | UUUKWUUU | 8 | 7895 | 1.30E-06 | MEME |
| 6 | STREME | 5-CAGCUACU | STREME-5 | CAGCUACU | 8 | 2878 | 7.20E-06 | STREME |
| 7 | STREME | 6-CUGCAG | STREME-6 | CUGCAG | 6 | 4349 | 1.70E-05 | STREME |
| 8 | STREME | 7-SGWGGUGG | STREME-7 | SGWGGUGG | 8 | 1360 | 3.50E-05 | STREME |
| 9 | STREME | 8-CCRCCUCC | STREME-8 | CCRCCUCC | 8 | 2929 | 6.50E-05 | STREME |
| 10 | STREME | 9-GAAGAUGG | STREME-9 | GAAGAUGG | 8 | 3157 | 1.20E-04 | STREME |
| 11 | STREME | 10-AGGUA | STREME-10 | 10-AGGUA | 5 | 3194 | 1.30E-04 | STREME |
| 12 | STREME | 11-CCCAGCCC | STREME-11 | 11-CCCAGCCC | 8 | 2652 | 1.60E-04 | STREME |
| 13 | STREME | 12-UUAGUAGA | STREME-12 | 12-UUAGUAGA | 8 | 183 | 2.00E-04 | STREME |
| 14 | STREME | 13-CAACWWC | STREME-13 | 13-CAACWWC | 7 | 3837 | 2.00E-04 | STREME |
| 15 | STREME | 14-GCGGCGGC | STREME-14 | 14-GCGGCGGC | 8 | 927 | 2.20E-04 | STREME |
| 16 | STREME | 15-AAAUACAA | STREME-15 | 15-AAAUACAA | 8 | 3034 | 2.40E-04 | STREME |
| 17 | STREME | 16-UAAUCCCA | STREME-16 | 16-UAAUCCCA | 8 | 233 | 2.50E-04 | STREME |
| 18 | STREME | 17-UUUUAAA | STREME-17 | 17-UUUUAAA | 7 | 1821 | 3.60E-04 | STREME |
| 19 | STREME | 18-GURAGUR | STREME-18 | 18-GURAGUR | 7 | 1573 | 3.80E-04 | STREME |
| 20 | STREME | 19-GCACUUUG | STREME-19 | 19-GCACUUUG | 8 | 285 | 4.50E-04 | STREME |
| 21 | STREME | 20-AAAUAUU | STREME-20 | 20-AAAUAUU | 7 | 2398 | 4.80E-04 | STREME |
| 22 | STREME | 21-GUCAYC | STREME-21 | 21-GUCAYC | 6 | 2489 | 6.30E-04 | STREME |
| 23 | STREME | 22-GCAGUGGC | STREME-22 | 22-GCAGUGGC | 8 | 1695 | 7.10E-04 | STREME |
| 24 | STREME | 23-GGSCGDGG | STREME-23 | 23-GGSCGDGG | 8 | 3522 | 8.20E-04 | STREME |
| 25 | STREME | 24-GAUGA | STREME-24 | 24-GAUGA | 5 | 4831 | 1.00E-03 | STREME |
| 26 | STREME | 25-CAUCUC | STREME-25 | 25-CAUCUC | 6 | 1680 | 1.30E-03 | STREME |
| 27 | STREME | 26-GACUUUGA | STREME-26 | 26-GACUUUGA | 8 | 3600 | 1.40E-03 | STREME |
| 28 | STREME | 27-GCUGCUG | STREME-27 | 27-GCUGCUG | 7 | 2404 | 1.80E-03 | STREME |
| 29 | STREME | 28-GAAGAA | STREME-28 | 28-GAAGAA | 6 | 2660 | 2.40E-03 | STREME |
| 30 | STREME | 29-AGGUGAG | STREME-29 | 29-AGGUGAG | 7 | 371 | 4.90E-03 | STREME |
| 31 | STREME | 30-UGAGGUCA | STREME-30 | 30-UGAGGUCA | 8 | 240 | 5.40E-03 | STREME |
| 32 | STREME | 31-CUUCUC | STREME-31 | 31-CUUCUC | 6 | 3096 | 5.90E-03 | STREME |
| 33 | STREME | 32-CUCACUGC | STREME-32 | 32-CUCACUGC | 8 | 294 | 7.00E-03 | STREME |
| 34 | STREME | 33-GCAGCAGC | STREME-33 | 33-GCAGCAGC | 8 | 750 | 8.30E-03 | STREME |
| 35 | STREME | 34-CAAGAAG | STREME-34 | 34-CAAGAAG | 7 | 907 | 9.30E-03 | STREME |
| 36 | STREME | 35-GGAGAAG | STREME-35 | 35-GGAGAAG | 7 | 1630 | 1.00E-02 | STREME |
| 37 | STREME | 36-UUAUUU | STREME-36 | 36-UUAUUU | 6 | 2471 | 1.20E-02 | STREME |
| 38 | STREME | 37-CAUCGWC | STREME-37 | 37-CAUCGWC | 7 | 3095 | 1.40E-02 | STREME |
| 39 | STREME | 38-UCAGCCUC | STREME-38 | 38-UCAGCCUC | 8 | 543 | 1.50E-02 | STREME |
| 40 | STREME | 39-CAGCCUGG | STREME-39 | 39-CAGCCUGG | 8 | 1604 | 1.80E-02 | STREME |
| 41 | STREME | 40-UCGGGGA | STREME-40 | 40-UCGGGGA | 7 | 2980 | 1.80E-02 | STREME |
| 42 | STREME | 41-UCUUCCU | STREME-41 | 41-UCUUCCU | 7 | 6299 | 1.80E-02 | STREME |
| 43 | STREME | 42-GAAUGUGG | STREME-42 | 42-GAAUGUGG | 8 | 326 | 2.30E-02 | STREME |
| 44 | STREME | 43-CAAGKAC | STREME-43 | 43-CAAGKAC | 7 | 2621 | 2.40E-02 | STREME |
| 45 | STREME | 44-GGAUUACA | STREME-44 | 44-GGAUUACA | 8 | 254 | 2.60E-02 | STREME |
| 46 | STREME | 45-CCGGGA | STREME-45 | 45-CCGGGA | 6 | 1460 | 3.20E-02 | STREME |
| 47 | STREME | 46-CUCUACUA | STREME-46 | 46-CUCUACUA | 8 | 234 | 3.90E-02 | STREME |
| 48 | STREME | 47-AGGUAA | STREME-47 | 47-AGGUAA | 6 | 1095 | 3.90E-02 | STREME |
| 49 | STREME | 48-CACUGCAC | STREME-48 | 48-CACUGCAC | 8 | 238 | 4.70E-02 | STREME |
