# Supplementary material for: circEIF3I facilitates the recruitment of SMAD3 to early endosomes to promote TGF-β signalling pathway-mediated activation of MMPs in pancreatic cancer
Source: Mol Cancer. 2023 Sep 9;22:152. doi: 10.1186/s12943-023-01847-2 (PMC10492306; doi:10.1186/s12943-023-01847-2)
Supplement: Supplementary file 14 — Additional file 14: Supplementary Table S6. Alignment of circEif3i (mmu_circ_0001266) and circEIF3I (hsa_circ_0011385). [file 12943_2023_1847_MOESM14_ESM.docx]

**Table S6. Alignment of circEif3i (mmu_circ_0001266) and circEIF3I (hsa_circ_0011385)**.

Identity = 89.56% (249/278)

mmu_circ_0001266 GGAAGCAGCTGGCCCTACTCAAGACCAACTCAGCTGTCCGGACCTGCGGCTTTGACTTTG 60

hsa_circ_0011385 GAAAGCAGCTGGCCCTTCTCAAGACCAATTCGGCTGTCCGGACCTGCGGTTTTGACTTTG 60

* ************** *********** ** ***************** **********

mmu_circ_0001266 GGGGCAACATCATCATGTTCTCCACAGACAAGCAGATGGGGTATCAGTGCTTCGTGAGCT 120

hsa_circ_0011385 GGGGCAACATCATCATGTTCTCCACGGACAAGCAGATGGGCTACCAGTGCTTTGTGAGCT 120

************************* ************** ** ******** *******

mmu_circ_0001266 TCTTTGATCTGCGGGATCCAAGCCAGATCGACAGCAACGAGCCCTACATGAAGATCCCCT 180

hsa_circ_0011385 TTTTTGACCTGCGGGATCCGAGCCAGATTGACAACAATGAGCCCTACATGAAGATCCCTT 180

* ***** *********** ******** **** *** ******************** *

mmu_circ_0001266 GTAATGATTCCAAGATCACCAGTGCCGTCTGGGGCCCCCTCGGGGAGTGCGTCATCGCAG 240

hsa_circ_0011385 GCAATGACTCTAAAATCACCAGTGCTGTTTGGGGACCCCTGGGGGAGTGCATCATCGCTG 240

* ***** ** ** *********** ** ***** ***** ********* ******* *

mmu_circ_0001266 GCCACGAGAGCGGAGAGCTCAACCAGTATAGCGCCAAG 278

hsa_circ_0011385 GCCATGAGAGTGGAGAGCTCAACCAGTATAGTGCCAAG 278

**** ***** ******************** ******
