# Supplementary material for: circEIF3I facilitates the recruitment of SMAD3 to early endosomes to promote TGF-β signalling pathway-mediated activation of MMPs in pancreatic cancer
Source: Mol Cancer. 2023 Sep 9;22:152. doi: 10.1186/s12943-023-01847-2 (PMC10492306; doi:10.1186/s12943-023-01847-2)
Supplement: Supplementary file 15 — Additional file 15: Supplementary Table S7. Sequences of primers used in qRT-PCR. [file 12943_2023_1847_MOESM15_ESM.docx]

**Table S7. Sequences of primers used in qRT-PCR**

| Primer Name: | Sequences (5’ to 3’) |
| --- | --- |
| hsa_circRNA_103809-Forward | ACCTCTGTCAGCGAGTTCCAAC |
| hsa_circRNA_103809-Reverse | AATTTTCTGAACTGCCTGTAACTCC |
| hsa_circRNA_101263-Forward | CACCTCACAGTGGCTGAGTCAC |
| hsa_circRNA_101263-Reverse | CAGGCTGCAATCTCGATAATCC |
| hsa_circRNA_105055-Forward | CTGGCTCAATATCCATGTCTTCC |
| hsa_circRNA_105055-Reverse | GAAGACTTGAAGTCGCTGGAAGAC |
| hsa_circRNA_102034-Forward | AACAGACAAAGACAGCAGGTTCC |
| hsa_circRNA_102034-Reverse | TTCATCACTCTGTTCTGCTTCTGAG |
| hsa_circRNA_001846-Forward | ACTCCACTCCCATGTCCCTTG |
| hsa_circRNA_001846-Reverse | CCCCACTGATGAGCTTCCCTC |
| hsa_circRNA_000543-Forward | AAGTCAGATCAGTTATGGGACAATAG |
| hsa_circRNA_000543-Reverse | AGAAAAAGCTTGTTCACATGCC |
| hsa_circRNA_104168-Forward | GAAAGGAGTCCATTATCGCTGG |
| hsa_circRNA_104168-Reverse | CATCCCATGCTTTCATTACCTGT |
| hsa_circRNA_103468-Forward | CCAGATCACTGATAGCACCATGC |
| hsa_circRNA_103468-Reverse | CAGTCATGTCTTTTGTTAGGCAAGG |
| hsa_circRNA_100146-Forward | TCAACCAGTATAGTGCCAAGGAAAG |
| hsa_circRNA_100146-Reverse | TGTCCGTGGAGAACATGATGATG |
| hsa_circRNA_100571-Forward | TGGACTTCACCTCGTGTTCTGAC |
| hsa_circRNA_100571-Reverse | CGTACTGATAGGCGATCTCATGC |
| hsa-circEIF3I-Forward  (divergent primers) | TCAACCAGTATAGTGCCAAGGAAAG |
| hsa-circEIF3I-Reverse  (divergent primers) | TGTCCGTGGAGAACATGATGATG |
| hsa- EIF3I-Forward  (convergent primers) | ACCTGCGGTTTTGACTTTGGG |
| hsa- EIF3I -Reverse  (convergent primers) | GCAAGGGATCTTCATGTAGGGC |
| hsa-EIF3I-Forward | ACCAAATTCTTGGCGGCAACTCC |
| hsa-EIF3I-Reverse | GTTTCGAGGTCACTTCCGGATTATGG |
| hsa-GAPDH-Forward | CCTCTGACTTCAACAGCGACAC |
| hsa-GAPDH-Reverse | TGGTCCAGGGGTCTTACTCC |
| hsa-MMP1-Forward | ATGAAGCAGCCCAGATGTGGAG |
| hsa-MMP1-Reverse | TGGTCCACATCTGCTCTTGGCA |
| hsa-MMP2-Forward | GAGGACTACGACCGCGACAA |
| hsa-MMP2-Reverse | GCACACCACATCTTTCCGTCACT |
| hsa-MMP9-Forward | TACTGTGCCTTTGAGTCCG |
| hsa-MMP9-Reverse | TTGTCGGCGATAAGGAAG |
| hsa-MMP14-Forward | CCTTGGACTGTCAGGAATGAGG |
| hsa-MMP14-Reverse | TTCTCCGTGTCCATCCACTGGT |
| hsa-MMP19-Forward | GCAGTAGTGAACTGGATGCCATG |
| hsa-MMP19-Reverse | CAAAGGGCAGACACTCGGAACA |
| mmu_circ_Eif3i-Forward  (divergent primers) | ACCAGTATAGCGCCAAGGGAAG |
| mmu_circ_Eif3i-Reverse  (divergent primers) | CGAAGCACTGATACCCCATCTG |
| mmu-Eif3i-Forward  (convergent primers) | ACCTGCGGCTTTGACTTTG |
| mmu-Eif3i-Reverse  (convergent primers) | TCATGTAGGGCTCGTTGCTG |
| mmu-Eif3i-Forward | AACAGAGCGTCCTGTCAACTCG |
| mmu-Eif3i-Reverse | CTTGCCAATCCTGGTGGAGGTT |
| mmu-Gapdh-Forward | CATCACTGCCACCCAGAAGACTG |
| mmu-Gapdh-Reverse | ATGCCAGTGAGCTTCCCGTTCAG |
