# Supplementary material for: circEIF3I facilitates the recruitment of SMAD3 to early endosomes to promote TGF-β signalling pathway-mediated activation of MMPs in pancreatic cancer
Source: Mol Cancer. 2023 Sep 9;22:152. doi: 10.1186/s12943-023-01847-2 (PMC10492306; doi:10.1186/s12943-023-01847-2)
Supplement: Supplementary file 16 — Additional file 16: Supplementary Table S8. The list of antibodies used in this study. [file 12943_2023_1847_MOESM16_ESM.docx]

**Table S8. The list of antibodies used in this study.**

| **Antibodies** | **SOURCE** | **IDENTIFIER** |
| --- | --- | --- |
| MMP2 (WB 1:1000; IHC 1:100) | Proteintech Group | Cat#10373-2-AP |
| MMP9 (WB 1:1000; IHC 1:100) | Proteintech Group | Cat#10375-2-AP |
| MMP14 (WB 1:1000; IHC 1:100) | Abcam | Cat#ab51074 |
| GAPDH (WB 1:5000) | Proteintech Group | Cat#10494-1-AP |
| EIF3I (WB 1:1000) | Proteintech Group | Cat#11287-1-AP |
| SMAD3 (WB 1:1000; IP 1:100) | Cell Signaling Technology | Cat##9523 |
| p-SMAD3 (WB 1:1000) | Cell Signaling Technology | Cat##9520 |
| p-SMAD3 (IHC 1:100) | Boster Biological Technology | Cat#P00059 |
| HA-Tag (WB 1:1000) | Cell Signaling Technology | Cat##3724 |
| FLAG-Tag (WB 1:1000) | Cell Signaling Technology | Cat##14793 |
| SMAD3 (IF 1:50) | Santa Cruz Biotechnology | Cat#sc-101154 |
| TGFBR1 (WB 1:500) | Absin | Cat#abs131446 |
| TGFBR1 (IF 1:100) | Invitrogen | Cat#PA5-32631 |
| TGFBR2 (WB 1:2000) | Proteintech Group | Cat#66636-1-Ig |
| EEA1 (IF 1:100) | Cell Signaling Technology | Cat#C45B10 |
| AP2A1 (WB 1:1000; IF 1:100; IP 1:50) | Invitrogen | Cat#MA3-061 |
| TRIP10 (WB 1:2000) | Proteintech Group | Cat#10798-1-AP |
| PACSIN2 (WB 1:1000) | Proteintech Group | Cat#10518-2-AP |
| ANXA2 (WB 1:10000) | Abcam | Cat#ab178677 |
| Rabbit IgG（IP Assay-dependent） | Beyotime | Cat#A7016 |
| Mouse IgG（IP Assay-dependent） | Beyotime | Cat#A7028 |
