# Supplementary material for: circEIF3I facilitates the recruitment of SMAD3 to early endosomes to promote TGF-β signalling pathway-mediated activation of MMPs in pancreatic cancer
Source: Mol Cancer. 2023 Sep 9;22:152. doi: 10.1186/s12943-023-01847-2 (PMC10492306; doi:10.1186/s12943-023-01847-2)
Supplement: Supplementary file 17 — Additional file 17: Supplementary Methods. The methods and materials used in this research. [file 12943_2023_1847_MOESM17_ESM.docx]

**Methods**

**Patients and specimens**

PDAC and their paired adjacent normal tissue were collected from the Department of Pancreatic and Biliary Surgery (The First Affiliated Hospital of Harbin Medical University, Harbin, Heilongjiang, China) during 2014 - 2018. Samples were frozen in liquid nitrogen immediately after surgical resection for further RNA extraction, or fixed in 4% paraformaldehyde and embedded in paraffin for tissue microarray (TMA) construction. This study was approved by the Ethics Committee of the First Affiliated Hospital of Harbin Medical University.

**Cell lines and reagents**

The human pancreatic cancer cell lines PANC-1 and BxPC-3 were obtained from the American type culture collection. HEK-293T cells were purchased from the Chinese Academy of Sciences Cell Bank. Panc02 cells were purchased from Hunan Fenghui Biotechnology Co., Ltd. PANC-1, 293T, and Panc02 cells were routinely cultured in Dulbecco’s modified Eagle’s medium (Gibco, USA), and BxPC-3 cell line was grown in RPMI 1640 medium (HyClone, USA) supplemented with 10% fetal bovine serum (Gibco, USA), penicillin (100 U/ml) and streptomycin (100 μg/ml). All cells were cultured at 37 °C humidified atmosphere containing 5% CO_2_. The cells were treating with PMA (Solarbio), Marimastat (Selleck), TGF-β1 (Human/Mouse, GenScript), SIS3 (MedChemExpress) and Dyngo-4a (Selleck) according to the needs of particular experiments.

**RNA extraction, quantitative real-time PCR analyses and RNase R treatment**

Total RNA was isolated using AxyPrep Multisource Total RNA Miniprep Kit (Axygen Biosciences, USA) according to the manufacturer’s instructions. The RNA was reverse-transcribed into cDNA using the ReverTraAce qPCR RT Kit (FSQ-101, Toyobo Co. Ltd.) according to the manufacturer’s instructions. Quantitative real-time polymerase chain reaction (qRT-PCR) was performed on a 7500 FAST Real-Time PCR System (Applied Biosystems, USA) with the FastStart Universal SYBR Green Master (Roche Diagnostics Deutschland GmbH, GER). For treatment with RNase R, 3 μg RNA was incubated with 10U RNase R (Epicentre, USA) at 37°C for 10 mins, and then 70°C for 10 mins to inactivate the RNase R. GAPDH served as the endogenous references for circRNA and mRNA. The primer sequences are described in Supplementary Table S7. For miRNA quantification: Bulge-loop^TM^ miRNA qRT-PCR Primer Sets specific for miR-149-5p, miR-361-3p, miR-615-5p and miR-616-3p are designed by RiboBio (Guangzhou, China), U6 as the internal control.

**RNA fluorescence in situ hybridization** **(FISH)**

RNA FISH was performed according to details previously reported[1], with some modifications. Briefly, cells grown on glass culture slides were fixed with 4% formaldehyde for 30 mins at room temperature, followed by permeabilization with 0.5% Triton X-100 and dehydration with graded ethanol. After incubation in prehybridization buffer (Sangon Biotech, Shanghai) for 30 mins at 4°C, the cells were incubated in hybridization buffer (1 μL circEIF3I probes with 40 μL prehybridization buffer) for 5 min at 73°C, and then 37°C overnight. Next, wash the cells gradually in 4× saline sodium citrate (SSC), 2× SSC and 1× SSC at 42°C. Nuclei were counterstained with 4, 6–diamidino–2–phenylindole (DAPI) (Beyotime). Images were captured using Zeiss LSM 800 confocal microscope system (Carl Zeiss Jena, Oberkochen, Germany). FAM-labelled circEIF3I probes (5’ – AGG GCC AGC TGC TTT CCT TGG CAC TAT ACT GG - 3’) were synthesized by BersinBio (Guangzhou, China).

**In situ hybridization (ISH) analysis**

A digoxigenin-labelled circEIF3I oligonucleotide probe (5’ -GAG AAG GGC CAG CTG CTT TCC TTG GCA CTA TAC TGG TTG A-3’) and an ISH kit (MK10769-h) obtained from Boster (Wuhan, China) were used to detect the probe signals in PDAC tissues according to the manufacturer’s instructions. In brief, slides were deparaffinized in xylene and ethanol and incubated with Pepsin for 20 min at room temperature; after prehybridized at 40°C for 2 h, the tissue sections were hybridized overnight with circEIF3I probe at 40°C. Then the slides were washed with graded-diluted SSC at 37°C and incubated with antibody against digoxigenin for 1 h. The sections were then incubated with SABC-POD kit (Boster, Wuhan, China), and visualized via using diaminobenzidine (DAB) (Boster, Wuhan, China). In the end, the tissues sections were counterstained with hematoxylin, mounted and analyzed.

**Transwell assay**

The transwell assay was performed as described previously[2]. 4×10^4^ cells in 200 μL serum-free media were plated in the 8 μm pore size Falcon® inserts pre-covered with or without Matrigel (BD Biosciences, USA), and the lower chamber was filled with 600 μL medium containing 10% FBS. After incubating for 24 h or 48 h at 37 °C, cells on the upper compartments were removed, whereas invasive cells on the bottom surface of the filter were fixed in methanol and then stained with 0.1% crystal violet solution for 20 min respectively. Cells numbers in five randomly selected fields (20×) was counted, and all assays were performed in triplicate.

**Wound healing assay**

Transfected cells were seeded into 6-well plates, after treating with mitomycin C (10 μg/mL) for 2 h, a wound was created with a 200 μL pipette tip on the monolayer cell. Next, cells were incubated in serum-free medium (for PANC-1) or 1% serum medium (for BxPC-3) for 24 h. Images were acquired at 0 h and 24 h via Olympus microscope (10×). The percentage of wound healing was measured by ImageJ software.

**Orthotopic and metastatic mice models**

All animal studies were conducted under a protocol approved by the Institutional Review Board of the First Affiliated Hospital of Harbin Medical University. Male 6-week-old BALB/c nude mice were purchased from the Beijing Vital River Laboratory Animal Technology and maintained in SPF environment. Anesthesia was induced by preoperative intraperitoneal administration of 1.25% 2,2,2-Tribromoethanol (0.2 ml/ 10 g body weight). For orthotopic pancreatic cancer model, exponentially growing cells were trypsinized and resuspended in 50% Matrigel to a volume of 10μl per 3 × 10^5^ cells. A left subcostal incision in line with the left ear was made to expose the pancreas, followed by injecting 10 μL of the mixture (equal to 3 x 10^5^ cells) into the pancreas gently via a 33G needle. Then, gently returned pancreas to the abdomen and closed the abdominal wall. The mice were euthanized after 5 weeks of implantation, the size of the intrapancreatic tumors was measured and visible metastatic lesions was counted.

Liver metastasis model was established via a previously reported method [3], with some modifications. Briefly, a midline incision was performed to expose the portal vein, and then a total of 2 × 10^6^ (for PANC-1) or 5 × 10^5^ (for Panc02) cells in 100 μL PBS were injected slowly into the portal vein using 30G needle. Next, using cotton swabs for compression at the injection site to avoid hemorrhage. The abdominal wall was closed by a suture, and the mice were allowed to recover on a warming pad. The mice were euthanized in 4 weeks (for Panc02) or 8 weeks (for PANC-1) later, their liver metastases were enumerated.

**Western blotting**

The protocol was described previously[2]. Briefly, total proteins were extracted by radio-immunoprecipitation assay (RIPA) buffer (Beyotime, Beijing, China) supplemented with protease inhibitor cocktail and phosphatase inhibitor. Protein concentration was measured using BCA Protein Assay Kit (Beyotime, Beijing, China). Protein samples (20–40 μg/sample) were separated by using SDS-containing polyacrylamide gels and transferred onto nitrocellulose transfer (NC) membranes. After blocking with 5% non-fat milk for 2 h, the membranes were incubated with primary antibodies at 4 °C overnight. Afterwards, the membranes were incubated with fluorescent secondary antibodies (LI-COR, NE, USA) for 1 h at room temperature (RT) and were visualized via Odyssey Imager Dual-color infrared imaging System (LI-COR, NE, USA). Additionally, GAPDH served as the internal references, the level of protein expression was calibrated to the band density of GAPDH. The antibodies used are listed in Supplementary Table S8.

**Zymography experiments**

The zymography experiments were performed using a previously reported method[4]. Cells were treated with serum-free medium contained PMA (10 ng/ml) for 24 h at 37 °C. The conditioned media (CM) were collected by centrifugation and then measured total protein concentration. The CM samples containing same quantity proteins were mixed with non-reducing SDS-PAGE sample buffer (Biosharp, BL511B) and loaded onto 10% polyacrylamide gel containing gelatin (1 mg/ml) by electrophoresis at 150 V for 60 min at 4 °C. The gels were washed in 0.25% Triton X-100 for 60 min at room temperature (RT) to remove the SDS, and incubated with incubation buffer (50 mM Tris-HCl, pH 7.5, 150 mM NaCl, 5 mM CaCl_2_, 1 μM ZnCl_2_ and 0.02% Brij-35) for 16 h at 37 °C. Gels were stained with 0.25% Coomassie blue R-250 (Beyotime, ST1123-5g) and destained with 7% acetic acid appropriately. Gelatinolytic activities appeared as clear bands of digested gelatin against a blue background of stained gelatin.

**Immunohistochemistry (IHC)**

IHC were performed according to details previously reported[5, 6]. Briefly, paraffin-embedded tissue sections (5μm) were immunostained with anti-MMP2 (10373-2-AP, Proteintech), anti-MMP9 (10375-2-AP, Proteintech), anti-MMP14 (ab51074, Abcam) and anti-Phospho-SMAD3 (P00059, Boster). The number of positive cells was counted in five randomly selected microscopic fields (Nikon, Japan).

**MS2-TRAP (MS2-tagged RNA affinity purification) assay**

MS2-circEIF3I or MS2 plasmids were transfected into cells, together with MS2-GST plasmids. After 48 h of culture, the cells were harvested with lysis buffer containing 20 mM Tris-HCl at pH 7.5, 100 mM KCl, 5 mM MgCl_2_, 0.5% Nonidet P-40 (NP-40), 10 mM dithiothreitol (DTT), complete protease inhibitors cocktail (Roche) and RNase inhibitor (Invitrogen). Then, the supernatant was collected after centrifugation. The pre-cleared Mag-Beads for GST fusion protein purification (Sangon Biotech, Shanghai) were incubated with cell lysates at 4 °C for 3 h with agitation before washing 5 times in NT2 buffer (50 mM Tris-HCl at pH 7.5, 150 mM NaCl, 1 mM MgCl_2_ and 0.05% NP-40) and eluted with Protein Elution Buffer (50 mM Tris–HCl at pH 8.0, 10 mM GSH). The collected proteins were stored for subsequent mass spectrometry. The plasmids of MS2-circEIF3I, MS2 and MS2-GST were designed and synthesized by BersinBio (Guangzhou, China).

**RNA immunoprecipitation (RIP) assay**

The RIP experiments were performed using a previously reported methods[7], with some modifications. Briefly, cells were lysed in polysome lysis buffer (100 mM KCl, 5 mM MgCl_2_, 10 mM Hepes, pH 7.0, 0.5% NP-40, 1 mM DTT) supplemented with RNase OUT (Invitrogen) and complete protease inhibitor cocktail (Thermo Scientific). The lysates were incubated with SMAD3, AP2A1 or control immunoglobulin (IgG) antibody for 3 h up to overnight at 4 °C followed by incubation with protein A/G magnetic beads (Thermo Scientific) for 1 h at 4 °C on rotating wheel. After 5 washes with NT2 buffer, the complexes were incubated with 0.1% SDS and 1.2 mg/mL Proteinase K at 55 °C for 45 min. RNA was extracted from supernatant using phenol: chloroform: isoamyl alcohol (25:24:1), and then used for qRT-PCR analysis. The primers and antibodies used are listed in Supplementary Table S7 and Supplementary Table S8, respectively.

***In vitro* RNA cyclization**

Linear RNA was synthesized by *in vitro* transcription using T7 RNA Polymerase (Vazyme, DD4101-PC) following the manufacturer’s instructions. *In vitro* cyclization of linear RNA was performed according to a previously reported method[8] with some modifications. Briefly, splint ligation circRNA was generated by treatment of linear RNA and DNA splint (AGT TGG TCA TAT CAC GGT TCC TTT CGT CGA CCG GGA AGA G) with T4 DNA ligase 1 overnight (16 h) at 16℃. circRNA was isolated following DNase I and RNase R treatment at 37℃ for 30 min treatment.

**RNA pull-down assay**

CircRNA pull-down assay was performed according to a previously published protocol [9], with some modifications. 293T cells were transfected with circEIF3I-overexpression plasmids and cultured about 48 h. Total RNA extracted from transfected 293T cells or circEIF3I synthesised *in vitro* were incubated with 100 pmol biotin-labelled probes in Hybridization Cocktails (Sangon Biotech, Shanghai) at 73°C for 5 min. Then RNA was slowly cooled down to room temperature (RT) and 50 μL streptavidin magnetic beads was added and incubated at RT for 30 min with rotation. After washing 3 times with 20 mM Tris-HCl (pH 7.5), 200 μl 1× RNA-protein binding buffer with 200 μg total protein from PANC-1 and BxPC-3 was added to the tube containing streptavidin magnetic beads. After incubated 1 h at 4 °C with rotation, the beads were washed 3 times with washing buffer, and then the potential interacting proteins were evaluated for western blot analysis. Biotin-labelled oligonucleotide probes (GCT TTC CTT GGC ACT ATA CT-3’bio) targeting junction site of circEIF3I or negative control were synthesized by RIBOBIO (Guangzhou, China).

**Co-immunoprecipitation (co-IP)**

Cells cultured in 10 cm dish were lysed with 500 μL of Pierce™ IP lysis buffer (Thermo Scientific) for 10 min on ice. Then, the lysates were collected after centrifugation and pre-cleared with IgG antibody. The lysates were incubated with 5 μg antibodies or control IgG while agitating at 4°C overnight. Next, 25 μL protein A/G magnetic beads (Thermo Scientific) were added and then incubated 2 h at 4°C on a rotator. After 3 washes with lysis buffer, the beads containing target antigen were collected with a magnetic stand, and then used for western blotting as described above. The antibodies used are listed in Supplementary Table S8.

**Potassium depletion**

Potassium depletion assay was performed according to details previously reported[10]. Cells cultured in 6 well plates were treated with 1% serum medium for 18–24 h. Media were then switched to hypotonic media (50% medium, 50% H_2_0). After 10 min treatment in hypotonic buffer, the cells were either switched to isotonic media without potassium (10 mM Tris-HCl, pH 7.5, 150 mM NaCl) or isotonic media containing 10 mM KCl. Cells were depleted for 30 min, the following were treatments with TGFβ1(5ng/ml, GenScript) for 15min, and then lysed in RIPA buffer (Beyotime) for western blotting.

**RNA-protein interaction simulation and analysis**

The secondary structure of circRNA was predicted by Mfold. The best secondary structure has an initial ΔG of -78.50 kcal/mol, with -78.40, -77.80, -77.80, and -77.50 (kcal/mol) for the rest of top five models. Then we used RNA Composer to generate the 3D model of the circRNA by analyzing the secondary structure in dot-bracket format. As the structure of full-length SMAD and AP2A1 have yet to be elucidated, we built the 3D models of SMAD3 and AP2A1 by Alfafold2 (DeepMind). Next, HDOCK was used to carry out the in-silico molecular docking between circRNA and AP2A1 or SMAD3, respectively. After comprehensively considering factors such as shape complementarity, interfacial area size, atomic potential energy, and potential binding site information, we selected the complex model with the highest score, which can better represent the molecular interaction of circRNA, AP2A1 and SMAD3.

**Electrophoretic mobility shift assay (EMSA)**

Three 5'-biotinylated oligonucleotide (Probe1：5’- AGT GGA GAG CTC AAC CAG TAT AGT GCC AAG GAA AGC AGC TGG CCC TTC TCA AGA CCA ATT -3’; Probe2：5’- GGC TGT CCG GAC CTG CGG TTT TGA CTT TGG GGG CAA CAT CAT CAT GTT CTC CAC GGA CAA -3’; Probe3：5’- GAT CCG AGC CAG ATT GAC AAC AAT GAG CCC TAC ATG AAG ATC CCT TGC AAT GAC TCT AAA A -3’) was used as the probes. The probes were incubated with the protein extract from pancreatic cells at room temperature for 30 min. The entire reaction mixture was run on a non-denaturing 0.5×TBE 6% polyacrylamide gel for 1h at 80 V at 4°C and then transferred onto Biodyne® B nylon membranes (Pall Corporation). Signals were visualized with reagents included in the kit and ChemiDoc XRS (Bio-Rad Laboratories, UAS).

**Quantification and statistical analysis**

Statistical analysis was conducted with GraphPad Prism 8.0.2 (GraphPad Software, Inc) and SPSS 25.0 (International Business Machines Corporation). All experiments were performed in triplicate, and the data are shown as the mean ± standard deviation (SD). The Kaplan–Meier survival analysis, Student t test and Pearson correlation coefficient was used to determine the statistical significance. *P* < 0.05 represented a statistically difference.

**References**

1. Wang L, Bu P, Ai Y, Srinivasan T, Chen H, Xiang K, Lipkin S, Shen X: **A long non-coding RNA targets microRNA miR-34a to regulate colon cancer stem cell asymmetric division.** *eLife* 2016, **5**.

2. Hu J, Li L, Chen H, Zhang G, Liu H, Kong R, Chen H, Wang Y, Li Y, Tian F, et al: **MiR-361-3p regulates ERK1/2-induced EMT via DUSP2 mRNA degradation in pancreatic ductal adenocarcinoma.** *Cell Death Dis* 2018, **9:**807.

3. Limani P, Borgeaud N, Linecker M, Tschuor C, Kachaylo E, Schlegel A, Jang J-H, Ungethüm U, Montani M, Graf R, et al: **Selective portal vein injection for the design of syngeneic models of liver malignancy.** *American Journal of Physiology-Gastrointestinal and Liver Physiology* 2016, **310:**G682-G688.

4. Hung Y-C, Chen T-Y, Lee EJ, Chen W-L, Huang S-Y, Lee W-T, Lee M-Y, Chen H-Y, Wu T-S: **Melatonin decreases matrix metalloproteinase-9 activation and expression and attenuates reperfusion-induced hemorrhage following transient focal cerebral ischemia in rats.** *Journal of Pineal Research* 2008, **45:**459-467.

5. Li L, Chen H, Gao Y, Wang YW, Zhang GQ, Pan SH, Ji L, Kong R, Wang G, Jia YH, et al: **Long Noncoding RNA MALAT1 Promotes Aggressive Pancreatic Cancer Proliferation and Metastasis via the Stimulation of Autophagy.** *Mol Cancer Ther* 2016, **15:**2232-2243.

6. Ogawa K, Lin Q, Li L, Bai X, Chen X, Chen H, Kong R, Wang Y, Zhu H, He F, et al: **Aspartate β-hydroxylase promotes pancreatic ductal adenocarcinoma metastasis through activation of SRC signaling pathway.** *Journal of Hematology & Oncology* 2019, **12**.

7. Gagliardi M, Matarazzo MR: **RIP: RNA Immunoprecipitation.** *Methods Mol Biol* 2016, **1480:**73-86.

8. Chen YG, Kim MV, Chen X, Batista PJ, Aoyama S, Wilusz JE, Iwasaki A, Chang HY: **Sensing Self and Foreign Circular RNAs by Intron Identity.** *Mol Cell* 2017, **67:**228-238 e225.

9. Xie M, Yu T, Jing X, Ma L, Fan Y, Yang F, Ma P, Jiang H, Wu X, Shu Y, Xu T: **Exosomal circSHKBP1 promotes gastric cancer progression via regulating the miR-582-3p/HUR/VEGF axis and suppressing HSP90 degradation.** *Mol Cancer* 2020, **19:**112.

10. Runyan CE, Schnaper HW, Poncelet AC: **The role of internalization in transforming growth factor beta1-induced Smad2 association with Smad anchor for receptor activation (SARA) and Smad2-dependent signaling in human mesangial cells.** *J Biol Chem* 2005, **280:**8300-8308.
